# Supplementary material for: Shedding light on blue-green photosynthesis: A wavelength-dependent mathematical model of photosynthesis in Synechocystis sp. PCC 6803
Source: PLoS Comput Biol. 2024 Sep 12;20(9):e1012445. doi: 10.1371/journal.pcbi.1012445 (PMC11421815; doi:10.1371/journal.pcbi.1012445)
Supplement: S1 Appendix — Contains lists of all parameter values used in the model (Table A), all modeled reactions (Table B), initial conditions (Table C), parameters used to model light-adapted cells (Table D), and ranges of parameter variation during state transition model analysis (Table E). We also include explanations of the reaction kinetics and Gibbs energy calculations used, as well as further analyses of the model. (PDF) [file pcbi.1012445.s001.pdf]

Supplemental Materials: Shedding light on blue-green photosynthesis

Contents

S1 Model structure **1**
S1.1 A complete summary of the modelled reactions . . . 4
S1.2 Simplified irreversible mass action kinetics . . . 5
S1.3 Description of photosystems . . . 6
S1.4 Exemplary Gibbs free energy calculation . . . 7
S1.5 Calculating pigment association . . . 7
S1.6 Possible mechanisms of state transitions . . . 8
S1.7 Estimating pathway fluxes . . . 8
S1.8 Estimating fluorescence parameters and heat quenching . . . 9
S1.9 RuBisCO reactions . . . 9
S1.10 Flavodiiron proteins . . . 9
S1.11 The orange carotenoid protein . . . 10
S1.12 Cellular import of CO_2 . . . 10

S2 Model parametrization **10**
S2.1 Model parameters . . . 10
S2.2 Calculating model parameters from experimental data . . . 11
S2.3 Calculating the mean light in a light absorbing culture . . . 12
S2.4 Robustness analysis . . . 12

S3 Additional model validation **12**
S3.1 Dynamics of photoprotection . . . 12
S3.2 Internal PSII states . . . 12

S4 Additional analysis **12**
S4.1 Mutant analysis . . . 12
S4.2 Activation of OCP in blue light . . . 13
S4.3 Metabolic Control Analysis (MCA) . . . 13
S4.4 Analysis of state transition models under light variation . . . 13
S4.5 Overexpression analysis . . . 13
S4.6 Analysis of productivity by light-adapted cells . . . 13

S1 Model structure

Our model consists of a system of 17 coupled Ordinary Differential Equations (ODEs), 24 reaction rates, and 95 parameters, including measured midpoint potentials, compound concentrations, absorption spectra, and physical constants. For a list of the model parameters see Table A and for a full list of reactions Table B. We have been using integration methods from the modelbase package [1] to solve the system with the initial values summarised in Table C. Parameters necessary for additional analyses are given in Table D (light adaption of cells) and Table E (parameter variation of state transition models).

Table A: **The model parameters with descriptions and sources.** Parameters with source "derived" are calculated from other parameters. "manually fitted" parameters were set in an initial step by manually fitting simulations to data or expected behavior. We then performed a robustness analysis with these parameters and continued to use values of the parameter set minimizing the residuals to data used for fitting (see Methods for an explanation of the residual calculation). See Table C for concentration parameters. For the parameter calculations, see the GitLab file *calculate_parameters_restruct.py*.

Parameter	Value	Description	Source value	Source
		Cell specific		
V_cell	5.600e-15 [l cell^-1]	synechocystis cell volume	5.6e-15 [l cell^-1] synechocystis cell volume	[2]
n_chl	1.400e+07 [cell^-1]	total chlorophyll content	1.4e7 [cell^-1] total chlorophyll content	[3]
bHi	100.000 [unitless]	buffering constant of the thylakoid lumen	estimated, like Ebenhöh (2014)	estimated
bHo	1.111e+03 [unitless]	buffering constant of the cytoplasm, assumed to be 1/f_V_lumen times larger	bHi, f_V_lumen	estimated
f_V_lumen	9.000e-02 [unitless]	thylakoid lumen fraction of the total cell volume	0.09 [unitless] thylakoid lumen fraction of the total cell volume	[4]
V_cyt	5.096e-15 [l cell^-1]	volume of the cytoplasm	f_V_lumen, V_cell	derived
V_lumen	5.040e-16 [l cell^-1]	volume of the thylakoid lumen	f_V_lumen, V_cell	derived
cChl	4.151e-03 [mol l^-1]	total molar concentration of chlorophyll	n_chl, V_cell, NA	derived
chl_cyt	4.562e-03 [mol l^-1]	molar concentration of chlorophyll relative to the cytoplasmic volume	n_chl, V_cyt, NA	derived
chl_lumen	4.613e-02 [mol l^-1]	molar concentration of chlorophyll relative to the lumen volume	n_chl, V_lumen, NA	derived
cf_lumen	4.613e-05 [mol(Chl) ml^-1]	conversion factor for [mmol mol(Chl)^-1] -> [mol l^-1] for the thylakoid lumen	chl_lumen	derived
cf_cytoplasm	4.562e-06 [mol(Chl) ml^-1]	conversion factor for [mmol mol(Chl)^-1] -> [mol l^-1] for the cytoplasm	chl_cyt	derived
fCin	1032.38 [unitless]	ratio of intracellular to external CO2 partial pressure with activity of the CCM	the CCM-increased intracellular bicarbonate concentration can exceed the external by 100 - 1000 times, manually fitted to Fig 2H	[5], manually fitted
HPR	4.667 [unitless]	number of protons (14) passing through the ATP synthase per ATP (3) synthesized	14.0 protons per full rotation of ATP synthase	[6]
pigment_content	{chla: 1.000, beta_carotene: 0.176, allophycocyanin: 1.118 , phycocyanin: 6.765} [mg(Pigment) mg(Chla)^-1]	relative pigment concentrations in a synechocystis cell		[7]
PBS_free	9.000e-02 [unitless]	fraction of unbound PBS	measured PBS fluorescence at 77K	[7]
PBS_PS1	0.390 [unitless]	fraction of PBS bound to PSI	measured PBS fluorescence at 77K	[7]
PBS_PS2	0.510 [unitless]	fraction of PBS bound to PSII	measured PBS fluorescence at 77K	[7]
fluo_influence	{PS2: 1.087, PS1: 1.058, PBS: 1.265} [unitless]	factors multiplied to the calculated fluorescence (no effect at 1)	manually fitted to Fig 3A and 3B	manually fitted
lcf	0.485 [excitations photons^-1]	light conversion factor used for photosystem excitations, OCP activation, and PBS fluorescence	manually fitted to reproduce 15 electrons PSI^-1 s^-1 in Fig 2B	manually fitted
		Physical constants		
F	96.485 [C mmol^-1]	Faraday's constant	9.6485e4 [C mol^-1] Faraday's constant	[8]
R	8.300e-03 [J K^-1 mmol^-1]	ideal gas constant	8.3145 [J K^-1 mol^-1] ideal gas constant	[8]
T	298.150 [K]	temperature	25 [°C] temperature	experimental condition
NA	6.022e+23 [mol^-1]	avogadros number	6.0221e23 [mol^-1] avogadros number	[8]
M_chl	893.509 [g mol^-1]	molar mass of chlorophyll a	893.509 [g mol^-1] molar mass of chlorophyll a (C55H72MgN4O5)	[9]
M_CO2	44.010 [g mol^-1]	molar mass of CO2	44.01 [g mol^-1] molar mass of CO2 (CO2)	[9]
DeltaG0_ATP	30.600 [kJ mol^-1]	energy of ATP formation	30.6 [kJ mol^-1] energy of ATP formation	[10]
		Concentrations		
PSIItot	0.830 [mmol mol(Chl)^-1]	total concentration of photosystem II complexes	measured photosystems fluorescence at 77K	[7]
PSItot	3.270 [mmol mol(Chl)^-1]	total concentration of photosystem I complexes	measured photosystems fluorescence at 77K	[7]
Q_tot	13.000 [mmol mol(Chl)^-1]	total PHOTOACTIVE PQ concentration	13 [1000(Chl)^-1] total PHOTOACTIVE PQ concentration (Khorobrykh2020)	[11]
PC_tot	1.571 [mmol mol(Chl)^-1]	total concentration of plastocyanin (PC_ox + PC_red)	22000 [cell^-1] total concentration of plastocyanin (PC_ox + PC_red)	[12]
Fd_tot	3.597 [mmol mol(Chl)^-1]	total concentration of ferredoxin (Fd_ox + Fd_red)	1.1 [unitless] ratio of total ferredoxin (Fd_ox + Fd_red) to PSI	[2]
NADP_tot	26.805 [mmol mol(Chl)^-1]	total concentration of NADP species (NADP + NADPH)	30 [nmol mg(Chl)^-1] total concentration of NADP species (NADP + NADPH)	[13]
NAD_tot	11.169 [mmol mol(Chl)^-1]	total concentration of NAD species (NAD + NADH)	50 [mM OD^-1] total concentration of NAD species (NAD + NADH)	[14]
AP_tot	430.143 [mmol mol(Chl)^-1]	total concentration of adenosine species (ADP + ATP)	400 [pmol (10^8 cells)^-1] cellular content of ATP	[15]
Pi_mol	1.000e-02 [mmol mol(Chl)^-1]	molar concentration of phosphate	1.000e-02 [mmol mol(Chl)^-1] molar concentration of phosphate	[10]
S	35.000 [unitless]	salinity within a cell	35 [unitless] salinity of sea water since no cell estimations could be found	[16]
cuvette_Chlconc	0 [mmol(Chl) m^-3]	chlorophyll concentration in the measured sample, used for calculation of light attenuation	no light attenuation assumed if it wasn't measured in a particular experiment	estimated
		Standard electrode potentials		
E0_QA	-0.140 [V]	standard electrode potential of the reduction of PS2 plastoquinone A	-0.14 [V] midpoint potential of the reduction of PS2 plastoquinone A	[17]
E0_PQ	0.533 [V]	standard electrode potential of the reduction of free plastoquinone	0.12 [V] midpoint potential of the reduction of free plastoquinone	[17]
E0_PC	0.350 [V]	standard electrode potential of the reduction of free plastocyanin	0.35 [V] midpoint potential of the reduction of free plastocyanin	[17]
E0_P700	0.410 [V]	standard electrode potential of the reduction of the oxidized PS1 reaction center	0.48 [V] midpoint potential of the reduction of the oxidized PS1 reaction center	[17]
E0_FA	-0.580 [V]	standard electrode potential of the reduction of PS1 iron-sulfur cluster A	-0.58 [V] midpoint potential of the reduction of PS1 iron-sulfur cluster A	[17]
E0_Fd	-0.410 [V]	standard electrode potential of the reduction of free ferredoxin	-0.41 [V] midpoint potential of the reduction of free ferredoxin	[17]
E0_NADP	-0.113 [V]	standard electrode potential of the reduction of NADP to NADPH	-0.32 [V] midpoint potential of the reduction of NADP to NADPH	[18]
E0_succinate/fumarate	0.443 [V]	standard electrode potential of the reduction of fumarate to succinate	0.03 [V] midpoint potential of the reduction of fumarate to succinate	[18]

A Table **- continued**

Parameter	Value	Description	Source value	Source
		General reaction parameters		
kH0	5.000e+08 [s^-1]	rate constant of (unregulated) excitation quenching by heat	5e8 [s^-1] rate of (unregulated) excitation quenching by heat	[19]
kHst	1.000e+09 [s^-1]	rate constant of state transition regulated excitation quenching by heat	1e9 [s^-1] rate of qE regulated excitation quenching by heat	[19]
kF	6.250e+08 [s^-1]	rate constant of excitation quenching by fluorescence	6.25e8 [s^-1] rate of excitation quenching by fluorescence	[19]
k2	2.500e+09 [s^-1]	rate constant of excitation quenching by photochemistry	5e9 [s^-1] rate of excitation quenching by photochemistry, decreased by factor 0.5 in manual fit to achieve Fm/F0 ca. 1.6	[19, 20]
k_F1	1.014 [s^-1]	rate constant of excitation quenching via fluorescence	manually fitted to achieve low intensity PSI fluorescence	manually fitted
kPQred	250.000 [mol(Chl) mmol^-1 s^-1]	rate constant of PQ reduction via PS2	250 [mol(Chl) mmol^-1 s^-1] rate of PQ reduction via PS2	[10]
kPCox	2.500e+03 [mol(Chl) mmol^-1 s^-1]	rate constant of PC oxidation via PS1	2500 [mol(Chl) mmol^-1 s^-1] rate of PC oxidation via PS1	[10]
kFdred	2.500e+05 [mol(Chl) mmol^-1 s^-1]	rate constant of Fd reduction via PS1	2.5e5 [mol(Chl) mmol^-1 s^-1] rate of Fd reduction via PS1	[10]
k_Q	1.790e+03 [mol(Chl) mmol^-1 s^-1]	rate constant of PC reduction by the cytochrome b6f complex	1/10 [ms] reduction of half the total PC at the end of a high light pulse, increased by factor 10 in manual fit for full PQ oxidation in blue light	[21], manually fited
k_NDH	3.136 [mol(Chl) mmol^-1 s^-1]	rate constant of PQ reduction by NDH-2	20 [umol(electrons) mg(Chl)^-1 h^-1] rate of electron flux through NDH-2	[22]
k_SDH	0.430 [mol(Chl) mmol^-1 s^-1]	rate constant of PQ reduction by SDH	70 [umol(electrons) mg(Chl)^-1 h^-1] rate of electron flux through SDH	[22]
k_O2	12.926 [mol(Chl)^2 mmol^-2 s^-1]	rate constant of Fd oxidation by Flv 1/3	20 [umol(O2) mg(Chl)^-1 h^-1] rate of oxygen consumption by Flv 1/3 in dark-light transition	[23]
k_aa	1.073 [mol(Chl)^2 mmol^-2 s^-1]	rate constant of oxygen reduction via COX	4 [umol(O2) mg(Chl)^-1 h^-1] rate of oxygen reduction via COX, increased by factor 2 in manual fit to achieve a dark PQ reduction of ca. 20 %	[23], manually fitted
k_FN_fwd	3.730 [mol(Chl) mmol^-1 s^-1]	rate constant of NADP reduction by FNR	1/8 [ms] 8 ms time for reduction of half the NADP reduced during a saturating flash	[13]
k_FN_rev	53.364 [mol(Chl)^3 mmol^-3 s^-1]	rate constant of reverse flux through FNR in darkness	8.3 [umol(O2) mg(Chl)^-1 h^-1] rate of oxygen reduction in the dark, should roughly match the rate of electron flux trough FNR	[13]
kRespiration	3.506e-06 [mol(Chl)^3 mmol^-3 s^-1]	rate constant of 3PGA oxidation and fumarate reduction by glycolysis and the TCA cycle	8.3 [umol(O2) mg(Chl)^-1 h^-1] rate of oxygen reduction in the dark, reduced by factor 20 in manual fit to avoid over reduciton in darkness	[23]
kO2out	4.025e+03 [s^-1]	rate constant of oxygen diffusion out of the cell	62.05 [mmol mol(Chl)^-1 s^-1] O2 efflux out of the cell assuming an O2 membrane gradient of 0.064 uM	[24]
kCCM	4.025e+03 [s^-1]	rate constant of CO2 diffusion into the cell, assumed to be identical to kO2out	estimated from kO2out	estimated
k_pass	1.038e-02 [mmol mol(Chl)^-1 s^-1]	rate constant of protons leaking across the thylakoid membrane per delta pH	manually fitted to provide a low capacity flow dissipating the pH gradient	manually fitted
kATPsynth	10.509 [s^-1]	rate constant of ATP synthesis	manually fitted to supply the CBB	manually fitted
kATPconsumption	0.301 [s^-1]	rate constant of ATP consumption by processes other than the CBB	manually fitted to provide a low capacity flow consuming ATP	manually fitted
kNADHconsumption	10.774 [s^-1]	rate constant of NADH consumption by processes other than NDH	manually fitted to provide a reasonable NAD+/NADH ratio	manually fitted
k_ox1	0.211 [mol(Chl)^2 mmol^-2 s^-1]	rate constant of oxygen reduction via bd-type (Cyd) terminal oxidases	3 [umol(O2) mg(Chl)^-1 h^-1] approximate rate of oxygen reduction via bd-type (Cyd) terminal oxidases	[23]
KHillFdred	39.139 [mmol^nHillFdred mol(Chl)^-nHillFdred]	Flv binding constant of Fd_red in Hill kinetics, assuming half activity around 2.5 mmol mol(Chl)^-1	manually fitted to induce fast Flv activation at ca. 50 µmols m-2 s-1 white irradiance (Schuurmans, 2015)	manually fitted
nHillFdred	3.804 [unitless]	Hill constant of Fd_red binding to Flv, assuming strong cooperative binding (see Brown2019)	manually fitted to induce fast Flv activation at ca. 50 µmols m-2 s-1 white irradiance ([25])	manually fitted
vNQ_max	47.524 [mmol mol(Chl)^-1 s^-1]	maximal rate of NDH-1	manually fitted to achieve 30 % CET in Fig 2E	manually fitted to Fig 2E
KMNQ_Qox	1.225 [mmol mol(Chl)^-1]	Michaelis constant for Q_ox reduction by NDH-1	manually fitted to provide mostly constant NDH-1 flux into high light ([26])	manually fitted
KMNQ_Fdred	1.332 [mmol mol(Chl)^-1]	Michaelis constant for Fd_red oxidation by NDH-1	manually fitted to provide mostly constant NDH-1 flux into high light ([26])	manually fitted
		Regulation parameters		
kQuench	2.130e-03 [mmol^-1 mol(Chl)^-1 s^-1]	rate constant of PS2 quenching by Q_red	manually fitted to Fig 3A and 3B	manually fitted to Fig 3A and 3B
kUnquench	0.108 [s^-1]	rate constant of internal PS2 unquenching	manually fitted to Fig 3A and 3B	manually fitted to Fig 3A and 3B
KMUnquench	0.206 [mmol^ mol(Chl)^-1]	binding constant of PS2 unquenching inhibition by Q_red	manually fitted to Fig 3A and 3B	manually fitted to Fig 3A and 3B
kCBBactivation	3.685e-02 [s^-1]	rate constant of CBB activation by reduced ferredoxin	manually fitted to reproduce CBB activation within 2 mins in dark to light transition [27]	manually fitted
KMFdred	0.287 [mmol mol(Chl)^-1]	Michaelis constant of CBB activation by reduced ferredoxin	manually fitted to achieve CBB activation under at ca. 10 µmol m-2 s-1 light where respiration becomes repressed [25]	manually fitted
OCPmax	0.289 [unitless]	maximal fraction of PBS quenched by OCP	ca. 29% of PBS were quenched in WT with 220 µE m-2 s-1 blue green illumination, maximum assumed to be 10% higher, manually fitted to Fig 3A and 3B	[28], manually fitted
kOCPactivation	9.119e-05 [s^-1 (umol(Photons) m^-2 s^-1)^-1]	rate constant of OCP activation by absorbed light	manually fitted to Fig 3A and 3B	manually fitted
kOCPdeactivation	1.378e-03 [s^-1]	rate constant of constitutive OCP relaxation by thermal processes and FRP	manually fitted to Fig 3A and 3B	manually fitted
		CBB and PR parameters		
vCBB_max	51.301 [mmol mol(Chl)^-1 s^-1]	approximate maximal rate of the Calvin Benson Bassham cycle	0.2e-15 [g(CO2) cell^-1 s^-1] approximate maximal CO2 uptake rate	[29]
vOxy_max	16.059 [mmol mol(Chl)^-1 s^-1]	approximate RuBisCO oxygenation rate	1 [s^-1] approximate RuBisCO oxygenation rate	[30]
KMATP	24.088 [mmol mol(Chl)^-1]	Michaelis constant for ATP consumption in the CBB cycle	0.1 [mmol l^-1] order of magnitude of KM_ATP for phosphoribulokinase (Wadano1998) and phosphoglyceratekinase	[31, 32]
KMNADPH	18.066 [mmol mol(Chl)^-1]	Michaelis constant for NADPH consumption in the CBB cycle	0.075 [mmol l^-1] approximate KM_NADPH for GAP2 in Synechocystis	[33]
KMCO2	72.264 [mmol mol(Chl)^-1]	Michaelis constant for CO2 consumption by cyanobacterial RuBisCO	0.3 [mmol l^-1] order of magnitude for KM_CO2 of cyanobacterial RuBisCO	[30]
KICO2	72.264 [mmol l^-1]	inhibition constant of CO2 for O2 consumption by cyanobacterial RuBisCO, assumed equal to KMCO2	0.3 [mmol l^-1] order of magnitude for KM_CO2 of cyanobacterial RuBisCO	estimated
KMO2	240.880 [mmol mol(Chl)^-1]	Michaelis constant for O2 consumption by cyanobacterial RuBisCO	1 [mmol l^-1] order of magnitude for KM_O2 of cyanobacterial RuBisCO	[30]
KIO2	240.880 [mmol mol(Chl)^-1]	inhibition constant of O2 for CO2 consumption by cyanobacterial RuBisCO, assumed equal to KMO2	1 [mmol l^-1] order of magnitude for KM_O2 of cyanobacterial RuBisCO	estimated
KMPGA	0.108 [mmol mol(Chl)^-1]	Michaelis constant limiting oxygenation reactions for low 3PGA	manually fitted to not limit respiration or photorespiratioo	manually fitted
kPR	1.523e-06 [mol(Chl)^4 mmol^-4 s^-1]	rate constant of (2-phospho)glycolate recycling into 3PGA	4.80e-6 [umol min^-1 ug(Chl)^-1] estimated rate of photorespiration (glycolate appearance) under Air-like CO2 concentration	[34]

Table B: **The model reactions with stoichiometry.** The reactions are grouped according to electron transport pathways or similar functions. Parentheses group logically connected compounds, while square brackets mark compounds that are necessary for mass balance but are not part of the model.

Description	Reaction	Name in code
	Linear Electron Transport	
Light reaction of PSII	$(PQ + 2\,H_o^+)\,[+H_2O] \longleftrightarrow PQH_2 + (2\,H_i^+ + 0.5\,O_2)$	PS2
Cytochrome b_6f complex	$PQH_2 + 2\,PC_{ox} + 2\,H_o^+ \longleftrightarrow (PQ + 2\,H_i^+) + 2\,PC_{red} + 2\,H_i^+$	b6f
Light reaction of PSI	$PC_{red} + Fd_{ox} \longleftrightarrow PC_{ox} + Fd_{red}$	PS1
Ferredoxin-NADP$^+$ Reductase	$(NADP^+ + H_o^+) + 2\,Fd_{red} \longleftrightarrow NADPH + 2\,Fd_{ox}$	FNR
	Respiratory Electron Transport	
Respiration of sugar compounds with multiple pathways	$3PGA + 7.402\,e\text{-}02\,fumarate + (0.567\,ADP + [0.567\,P_i]) + 2.237\,NADP^+ + 2.689\,NAD^+ \longleftrightarrow$<br>$3\,CO_2 + 7.402\,e\text{-}02\,succinate + 0.567\,ATP + (2.237\,NADPH + 2.237\,H_o^+) + (2.689\,NADH + 2.689\,H_o^+)$	Respiration
Succinate dehydrogenase	$(PQ + 2\,H_o^+) + succinate \longleftrightarrow PQH_2 + (fumarate + 2\,H_o^+)$	SDH
NADH dehydrogenase-like complex type-2	$NADH + (PQ + 2\,H_o^+) \longleftrightarrow (NAD^+ + H_o^+) + PQH_2$	NDH
	Cyclic Electron Transport	
NADH dehydrogenase-like complex type-1	$2\,Fd_{red} + (PQ + 2\,H_o^+) + H_o^+ \longleftrightarrow 2\,Fd_{ox} + PQH_2 + H_i^+$	NQ
	Alternate Electron Transport	
bd-type terminal oxidase	$2\,PQH_2 + (O_2 + 4\,H_o^+) \longrightarrow (2\,PQ + 4\,H_i^+)\,[+2\,H_2O]$	bd
aa3-type terminal oxidase (active proton pump)	$4\,PC_{red} + (O_2 + 5\,H_o^+) \longrightarrow 4\,PC_{ox}\,[+2\,H_2O] + H_i^+$	aa
Flavodiiron protein dimer 1/3	$4\,Fd_{red} + (O_2 + 4\,H_o^+) \longrightarrow 4\,Fd_{ox}\,[+2\,H_2O]$	Flv
	ATP synthase and proton leak	
F0F1 ATPase	$(ADP + [P_i]) + HPR \cdot H_i^+ \longleftrightarrow ATP + HPR \cdot H_o^+$	ATPsynthase
Proton leakage across the thylakoid membrane	$H_i^+ \longrightarrow H_o^+$	Pass
	Calvin-Benson-Bassham cycle and Photorespiration	
RuBisCO carboxylation and Calvin-Benson-Bassham cycle	$(3\,CO_2 + 10\,H_o^+) + 8\,ATP + 5\,NADPH \longrightarrow 3PGA + 8\,ADP + (5\,NADP^+ + 5\,H_o^+)$	CBB
RuBisCO oxygenation (includes steps of the CBB)	$(2 \cdot 3PGA + 3\,O_2 + 10\,H_o^+) + 8\,ATP + 5\,NADPH \longrightarrow 3 \cdot PG + 8\,ADP + (5\,NADP^+ + 5\,H_o^+)$	Oxy
Photorespiratory salvage pathway (multiple mechanisms)	$2 \cdot PG + ATP + NADPH + 2\,NAD^+ \longleftrightarrow (CO_2 + 3PGA) + (ADP\,[+P_i]) + (NADP^+ + H_o^+) + 2\,NADH$	PRsalv
	Consuming reactions	
Cellular metabolic consumption of ATP	$ATP \longrightarrow ADP\,[+P_i]$	ATPconsumption
Cellular metabolic consumption of NADH	$NADH + (+2\,H_o^+\,[+C]) \longrightarrow (NAD^+ + H_o^+)\,[+CH_2]$	NADHconsumption
	Regulatory reactions	
Fd-dependent activation of the CBB	$CBB_{inactive} \longleftrightarrow CBB_{active}$	CBBactivation
PSII-internal quenching (mechanism BASED ON [35])	$PSII_{unquenched} \longrightarrow PSII_{quenched}$	PSIIquench
Relaxation of PSII-internal quenching	$PSII_{quenched} \longrightarrow PSII_{unquenched}$	PSIIunquench
Light-dependent activity of OCP	$OCP_{inactive} \longleftrightarrow OCP_{active}$	OCPactivation
	Gas exchange	
O_2 diffusion out of the cell	$O_2 \longleftrightarrow$	O2out
CCM driven CO_2 transport into the cell	$\longleftrightarrow CO_2$	CCM

Table C: **Initial conditions.** Initial values were set at the beginning of a simulation. For the calculations, see the GitLab file *calculate_parameters_restruct.py*.

Parameter	Value	Description	Source value	Source
PSII	1.232 [mmol mol(Chl)^-1]	initial concentration of unquenched PSII	estimated from cell being in state 2 in darkness	estimated
O2	55.402 [mmol mol(Chl)^-1]	concentration of oxygen in the cell	230 [umol l^-1] concentration of oxygen in air saturated water: 230 uM	[24]
PC_ox	0.157 [mmol mol(Chl)^-1]	initial concentration of oxidized plastocyanin (aerobic)	0.1 [unitless] fraction of oxidized plastocyanin (aerobic)	[36]
Fd_ox	3.324 [mmol mol(Chl)^-1]	initial concentration of oxidized ferredoxin (aerobic)	0.9 [unitless] fraction of oxidized ferredoxin (aerobic)	[36]
NADPH	20.104 [mmol mol(Chl)^-1]	initial concentration of NADPH	0.75 [unitless] fraction of reduced NADPH	[22]
NADH	3.574 [mmol mol(Chl)^-1]	initial concentration of NADH	0.32 [unitless] approximate fraction of reduced NADH	[14]
ATP	172.057 [mmol mol(Chl)^-1]	initial concentration of ATP	400 [pmol (10^8 cells)^-1] concentration of ATP	[15]
PG	0.894 [mmol mol(Chl)^-1]	initial concentration of (2-phospho) glycolate	concentration below 1e-6 [umol ug(Chl)^-1], the estimated detection limit of the method used by Huege (2011)	[34]
succinate	2.000 [mmol mol(Chl)^-1]	initial concentration of succinate	estimated	estimated
fumarate	2.000 [mmol mol(Chl)^-1]	initial concentration of fumarate	estimated	estimated
3PGA	2.000e+03 [mmol mol(Chl)^-1]	initial concentration of 3-phosphoglycerate (including all other sugars)	manually fitted to be non-limiting for RuBisCO oxygenation and respiration	manually fitted
CO2	3.103 [mmol mol(Chl)^-1]	concentration of CO_2 in the cell without activity of the CCM	322e-4 [mol l^-1 atm^-1] solubility of CO2 in 25 °C water with ~10 ‰ Cl^- ions	[37]
CBBa	0.000e+00 [unitless]	initial redox-regulated activity of the CBB	CBB should be inactive in the dark to avoid futile cycles	estimated
Hi	0.217 [mmol mol(Chl)^-1]	initial concentration of lumenal protons	pH 5 in 10^4 uE cm^-1 s^-1 light	[38]
Ho	6.932e-03 [mmol mol(Chl)^-1]	initial concentration of cytoplasmic protons	pH 7.5 in 10^4 uE cm^-1 s^-1 light	[38]
Q_ox	7.202 [mmol mol(Chl)^-1]	concentration of oxidized plastoquinone	0.446 [unitless] fraction of PHOTOACTIVE plastoquinone reduced in 40 umol m^-2 s^-1 light	[11]
OCP	0.000e+00 [unitless]	initial activity of OCP	OCP quenching is relaxed in the dark	estimated

S1.1 A complete summary of the modelled reactions

The model's main body consists of these four electron transport pathways:

- Linear Electron Transport (LET): Electrons originating from Photosystem II (PSII) and follow the redox potential from Plastoquinone (PQ) via Cytochrome b_6f complex (Cb_6f) to Plastocyanine (PC) to Photosystem I (PSI). In a second light reaction, electrons are excited again, transferred to Ferredoxin (Fd), and afterward to Nicotinamide adenine dinucleotide phosphate ($NADP^+$) by the Ferredoxin-$NADP^+$ Reductase (FNR).
- Respiratory Electron Transport (RET): Alternatively, electrons could originate from carbon compound respiration. These electrons can be transferred **a)** from the Tri-Carboxylic Acid cycle intermediate fumarate to PQ via succinate dehydrogenase; **b)** from reduced Nicotinamide adenine dinucleotide (NADH) to PQ via NADH dehydrogenase-like complex type-2; or **c)** backwards from reduced Nicotinamide adenine dinucleotide phosphate (NADPH) to Fd via FNR. These electrons typically leave the PETC through a terminal oxidase.
- Cyclic Electron Transport (CET): From Fd, the electrons can also travel back to PQ via the NAD(P)H Dehydrogenase-like complex 1 (NDH-1). The electrons, therefore, cycle around PSI.

Table D: **Parameters used for light-adapted strains.** The parameters were experimentally measured by Zavřel *et al.* (2024) [7] or Rodrigues *et al.* (2023) [39] or inferred from such measurements. Columns designate cells grown at a certain monochromatic light wavelength and intensity. The entries `chla`, `beta_carotene`, `allophycocyanin`, `phycocyanin` are part of the parameter `pigment_content`. `cuvette_Chlconc` was used for determining light attenuation. Default values are used in the case of empty cells. For parameter descriptions, see Table A.

	Zavrel (2024)								Rodrigues (2023)							Unit
Wavelength	435 nm	465 nm	495 nm	520 nm	555 nm	633 nm	663 nm	687 nm	405 nm	405 nm	450 nm	540 nm	540 nm	630 nm	630 nm	
Light intensity	25	25	25	25	25	25	25	25	50	100	50	50	100	50	100	µmol(photons) m^{-2} s^{-1}
PBS_PS2	0.597	0.5639	0.5992	0.568	0.53	0.5117	0.5167	0.615								unitless
PBS_PS1	0.3049	0.2909	0.2921	0.3071	0.3552	0.3891	0.4034	0.2773								unitless
PBS_free	0.09814	0.1452	0.1088	0.1249	0.1147	0.09921	0.07988	0.1076								unitless
PSIItot	1.818	1.221	1.221	1.093	0.8449	0.8314	0.936	2.031								mmol mol(Chl)$^{-1}$
PSItot	3.03	3.175	3.175	3.207	3.267	3.27	3.245	2.979								mmol mol(Chl)$^{-1}$
chla	1	1	1	1	1	1	1	1	1	1	1	1	1	1	1	mg(Pigment) mg(Chla)$^{-1}$
beta_carotene	0.1812	0.17	0.1723	0.1541	0.1418	0.1765	0.1729	0.1882	0.1437	0.1471	0.142	0.143	0.1412	0.1441	0.1442	mg(Pigment) mg(Chla)$^{-1}$
allophycocyanin	0.3906	0.025	0.1064	0.2838	0.4937	1.118	0.9792	0.7255	1.2295	1.6152	0.7983	0.8497	0.7942	0.8154	1.097	mg(Pigment) mg(Chla)$^{-1}$
phycocyanin	3.453	4.775	4.085	3.081	3.582	6.765	5.542	4.608	7.5436	9.449	5.3145	5.765	5.6851	5.4425	6.73	mg(Pigment) mg(Chla)$^{-1}$
cuvette_Chlconc	0.6797	0.09158	0.2174	0.9016	1.452	0.9766	0.9554	0.9257								mmol(Chl) m^{-1}

Table E: **State transition model parameters used in the model.** Each parameter is given with the lower and upper bound, where it was varied in the systematic perturbations.

Parameter	Default value	Lower bound	Upper bound	Unit
kUnquench	0.1	0.01	1	mmolˆ-1 mol(Chl) sˆ-1
kQuench	2e-3	2e-2	2e-4	mmolˆ-1 mol(Chl) sˆ-1
KMUnquench	0.2	0.01	0.3	mmol mol(Chl)ˆ-1
kspill	5e-3	5e-4	5e-2	mmolˆ-1 mol(Chl) sˆ-1
kunspill	5e-4	5e-5	5e-3	mmolˆ-1 mol(Chl) sˆ-1
spillmax	0.3	0.1	0.6	unitless
kPBS_toPS1	5e-3	5e-4	5e-2	mmolˆ-1 mol(Chl) sˆ-1
kPBS_toPS2	1e-3	1e-4	1e-2	mmolˆ-1 mol(Chl) sˆ-1
PBS_PS1min	0.25	0	0.5	mmol mol(Chl)ˆ-1
PBS_PS2min	0.35	0	0.5	mmol mol(Chl)ˆ-1
kPBS_detach	1e-4	1e-5	1e-3	mmolˆ-1 mol(Chl) sˆ-1
kPBS_attach	1e-3	1e-4	1e-2	mmolˆ-1 mol(Chl) sˆ-1
PBS_freemax	0.1	0.01	0.3	unitless

- Alternate Electron Transport (AEF): Lastly, electrons originating from PSII can also be transferred to O_2 by a terminal oxidase (bb-type, aa_3-type, or Flavodiiron protein dimer 1/3 (Flv)) without affecting the NADPH redox state.

Protons pumped during electron transport drive the Adenosine triphosphate (ATP) generation through ATP synthase (ATPS) or leak across the membrane. The Calvin-Benson-Bassham cycle (CBB)'s carbon fixation is the leading consumer of ATP and NADPH. Another sink is the consumption of Ribulose-1,5-bisphosphate Carboxylase-Oxygenase (RuBisCO) oxygenation side products through Photorespiration (PR) salvage. Additionally, consuming reactions for ATP and NADH simulate the remaining metabolic demand.

Both FNR and CBB are activated by reduced Fd while PQ induces PSII quenching. Lastly, O_2 diffuses across the cell membrane while the Carbon Concentrating Mechanism (CCM) actively imports CO_2. For a complete list of reactions, see Table B.

S1.2 Simplified irreversible mass action kinetics

Kinetic descriptions that don't follow Mass-Action (MA) or Eq (1) are given below. For simplifying the reversible MA law [40] we followed the approach of Noor *et al.* [41] by separating the kinetic and thermodynamic terms within the rate law. Exemplary for the forward direction ($\Delta_r G^{'} < 0$):

$$\mathrm{v} = \mathrm{k} \cdot \left(\prod c_{S_i}^{n_i} - \frac{\prod c_{P_j}^{m_j}}{K_{eq}} \right) = \overbrace{\mathrm{k} \cdot \prod c_{S_i}^{n_i}}^{\text{kinetic}} \cdot \overbrace{\left(1 - \frac{\prod c_{P_j}^{m_j} / \prod c_{S_i}^{n_i}}{K_{eq}} \right)}^{\text{thermodynamic ("}\gamma\text{")}} \tag{S1}$$

with $K_{eq} = \exp(-\Delta_r G^{'0}/\mathrm{RT})$ and $\Delta_r G^{'} = \Delta_r G^{'0} + RT \cdot \ln(\prod c_{P_j}^{m_j} / \prod c_{S_i}^{n_i})$ this can be written as

$$= \mathrm{k} \cdot \prod c_{S_i}^{n_i} \cdot \left(1 - \exp(\Delta_r G^{'}/\mathrm{RT}) \right) \tag{S2}$$

and simplifying only the kinetic term

$$v \approx \mathrm{k}^{+} \cdot \prod c_{S_i} \cdot \left(1 - \exp(\Delta_r G^{'}/\mathrm{RT}) \right) \tag{S3}$$

We did not alter the thermodynamic term to maintain v = 0 at the MA-derived equilibrium concentrations. We proceeded similarly for the reverse reaction ($\Delta_r G^{'} > 0$), factoring out $\prod c_{P_j}^{m_j}/K_{eq}$:

$$\mathrm{v} = \mathrm{k} \cdot \frac{\prod c_{P_j}^{m_j}}{K_{eq}} \cdot \left(\frac{K_{eq}}{\prod c_{P_j}^{m_j} / \prod c_{S_i}^{n_i}} - 1 \right) \tag{S4}$$

$$\approx \mathrm{k}^- \cdot \frac{\prod c_{P_j}}{K_{eq}} \cdot \left(\exp(-\Delta_r G^{'}/\mathrm{RT}) - 1 \right) \tag{S5}$$

By simplifying in S3 and S5 we approximate

$$k \cdot \prod c_{S_i}^{n_i} \approx k^+ \cdot \prod c_{S_i}^{n_i} / \prod c_{S_i}^{n_i - 1} \quad \text{and} \quad k \cdot \prod c_{P_j}^{m_j} \approx k^- \cdot \prod c_{P_j}^{m_j} / \prod c_{P_j}^{m_j - 1} \tag{S6}$$

which means for the relevant case of non-zero rates ($\prod c_{S_i}^{n_i} > 0$ and $\prod c_{P_j}^{m_j} > 0$)

$$k \approx \frac{\mathrm{k}^+}{\prod c_{S_i}^{n_i - 1}} \approx \frac{\mathrm{k}^-}{\prod c_{P_j}^{m_j - 1}} \tag{S7}$$

For any $n_i > 1$ or $m_j > 1$, the two denominators may not be equal and thus

$$\mathrm{k}^+ \neq \mathrm{k}^- \tag{S8}$$

S1.3 Description of photosystems

We model the photosystems using Quasi-Steady-State (QSS) equation systems similar to our previous models [19, 10] representing PSII as a four-state model and PSI as a three-state model (S1 Fig).

PSI was described similarly using a three-state QSS system. The open state Y_0 is excited to Y_1 (k: k_{L1}), which can donate an electron to Fd (k: k_{Fdred}). The resulting oxidized PSI (Y_2) is reduced by PC (k: k_{PCox}) to return to the open state. To represent PSI fluorescence, we have added a slow relaxation of Y_1 by fluorescence (k: k_{F1}). Because no estimate of k_{F1} was available in the literature, we varied its value during robustness analysis (S2 Fig). The resulting equation system

$$\frac{dY_0}{dt} = -(\frac{k_{PCox} PC_{ox}}{K_{eq,PCP700}} + k_{LI}) \cdot Y_0 + k_{F1} \cdot Y_1 + k_{PCox} PC_{red} \cdot Y_2 = 0 \tag{S9}$$

$$\frac{dY_1}{dt} = k_{LI} \cdot Y_0 - (k_{F1} + k_{Fdred} Fd_{ox}) \cdot Y_1 + \frac{k_{Fdred} Fd_{red}}{K_{eq,FAFd}} \cdot Y_2 = 0 \tag{S10}$$

$$Y_0 + Y_1 + Y_2 = PSI_{tot} \tag{S11}$$

is also symbolically solved at every integration step and used to determine the PSI reaction rate v_{PSI} as the net rate of excitations

$$v_{PSI} = k_{L1} \cdot Y_0 - k_{F1} \cdot Y_1 \tag{S12}$$

The four-state model of PSII consists of the open and closed reaction center states (B_0, B_2) as well as their respective excited states (B_1, B_3). The PSII excitation rate constant k_{LII} is calculated from E_{PSI} in Eq (2) (in µmol(photons) mg(chl)$^{-1}$) by multiplication with the molar mass of chlorophyll M_{Chl} and dividing by the PSII concentration:

$$k_{LII} = E_{PSI} \cdot M_{Chl} \cdot \frac{1}{c_{II}} \tag{S13}$$

The resulting excited states can be quenched by photochemistry (rate constant (k): k_2, only B_1), as heat (k: k_H) or fluorescence (k: k_F). PSII with closed reaction centers can also reversibly reduce PQ (k: k_{PQred}). The QSS assumption together with the conservation of PSII complexes results in the equation system:

$$\frac{dB_0}{dt} = -(k_{LII} + \frac{k_{PQred} PQ_{red}}{K_{eq,PQred}}) \cdot B_0 + (k_H + k_F) \cdot B_1 + k_{PQred} PQ_{ox} \cdot B_2 = 0 \tag{S14}$$

$$\frac{dB_1}{dt} = k_{LII} \cdot B_0 - (k_H + k_F + k_2) \cdot B_1 = 0 \tag{S15}$$

$$\frac{dB_3}{dt} = k_{LII} \cdot B_2 - (k_H + k_F) \cdot B_3 = 0 \tag{S16}$$

$$B_0 + B_1 + B_2 + B_3 = PSII_{tot} \tag{S17}$$

Note that state transitions affect k_H in the PSII quenching model (see Eq (S35)). We have simulated the fraction of PSII in the "open" states B_0 and B_1 under different light intensities (S3 Fig). We defined the PSII rate v_{PSII} as

$$v_{PSII} = 0.5 \cdot k_2 \cdot B_1 \tag{S18}$$

since two $B_1 \rightarrow B_2$ reactions have to occur for a full PQ reduction.

S1.4 Exemplary Gibbs free energy calculation

Here, we explain the calculation of Gibbs free energies for reaction kinetics on the example of FNR. First, we calculate the standard Gibbs free energy ($\Delta_r G^{'0}$) following Ebenhöh *et al.* [19]. We split the model reaction into redox pair reactions with electrons e^- and protons H^+:

$$\mathrm{Ox} + \mathrm{a\,e^-} + \mathrm{b\,H^+} \longleftrightarrow \mathrm{Red} \quad \text{with a, b} \in \mathbb{N} \tag{S19}$$

If the reaction doesn't involve free electrons, the sub reaction's $\Delta_r G^{'0}$ is calculated as [19]:

$$\Delta_r G^{'0} = -\mathrm{a} \cdot \mathrm{F} \cdot \mathrm{E_0} + \mathrm{b} \cdot ln(10) RT \cdot \mathrm{pH} \tag{S20}$$

With the Faraday constant F, the sub reaction's standard electrode potential E_0, the ideal gas constant R, and temperature T. The total $\Delta_r G^{'0}$ is then the stoichiometric sum of the sub reaction $\Delta_r G^{'0}$. We can decompose the FNR reaction (excluding the NADPH-associated proton)

$$2\,\mathrm{Fd_{red}} + \mathrm{NADP^+} + \mathrm{H^+} \longleftrightarrow 2\,\mathrm{Fd_{ox}} + \mathrm{NADPH} \tag{S21}$$

into the sub reactions

$$\mathrm{Fd_{ox}} + \mathrm{e^-} \longleftrightarrow \mathrm{Fd_{red}} \tag{S22}$$

$$\mathrm{NADP^+} + 2\,\mathrm{e^-} + \mathrm{H^+} \longleftrightarrow \mathrm{NADPH} \tag{S23}$$

with stoichiometry $(S21) = -2 \cdot (S22) + 1 \cdot (S23)$. We then calculate the $\Delta_r G^{'0}$ according to Eq (S20):

$$\Delta_r G^{'0}_{Fd} = -1 \cdot \mathrm{F} \cdot \mathrm{E_0} \tag{S24}$$

$$\Delta_r G^{'0}_{NADP} = -2 \cdot \mathrm{F} \cdot \mathrm{E_0} + 1 \cdot ln(10) RT \cdot \mathrm{pH} \tag{S25}$$

$$\Delta_r G^{'0}_{FNR} = -2 \cdot \Delta_r G^{'0}_{Fd} + 1 \cdot \Delta_r G^{'0}_{NADP} \tag{S26}$$

At each time step we now calculate Gibbs free energy $\Delta_r G^{'}$ using the reactant concentrations:

$$\Delta_r G^{'} = \Delta_r G^{'0} + RT \cdot \ln(\textstyle\prod c_{P_j}^{m_j} / \prod c_{S_i}^{n_i}) \tag{S27}$$

$$\Delta_r G^{'}_{FNR} = \Delta_r G^{'0}_{FNR} \cdot \frac{\mathrm{Fd^2_{red}} \cdot \mathrm{NADP^+}}{\mathrm{Fd^2_{ox}} \cdot \mathrm{NADPH}} \tag{S28}$$

With $\Delta_r G^{'}$ and $\Delta_r G^{'0}$ we can then calculate the reaction rate in Eq (1).

S1.5 Calculating pigment association

In addition to Phycobilisome (PBS), we calculate the fraction of light absorbed by chlorophyll and beta-carotene passed to the photosystems. For chlorophyll, the relationship is given as the ratio of their bound chlorophyll:

$$p_{Chl,I} = 1 - p_{Chl,II} = \frac{3c_I \cdot n_I^{Chl}}{3c_I \cdot n_I^{Chl} + 2c_{II} \cdot n_{II}^{Chl}} \tag{S29}$$

Similarly, we calculate the relative amounts of beta-carotene bound to either photosystem. However, as described by Fuente *et al.* [42], beta-carotene is also present outside of the photosystems. The photosystem stoichiometry requires a certain ratio of beta-carotene to chlorophyll.

$$f_{Chl:Car,stoich} = \frac{3c_I \cdot \frac{n_I^{Chl}}{n_I^{Car}} + 2c_{II} \cdot \frac{n_{II}^{Chl}}{n_{II}^{Car}}}{3c_I + 2c_{II}} \tag{S30}$$

We assume that any beta-carotene present in the cell above that ratio is not associated with the photosystems. Therefore we calculate the fraction beta-carotene absorption passed to photosystems f_{Car} as:

$$f_{Car} = \frac{f_{Chl:Car,stoich}}{f_{Chl:Car,meas}} \tag{S31}$$

$$p_{Chl,I} = \frac{3c_I \cdot n_I^{Car}}{3c_I \cdot n_I^{Car} + 2c_{II} \cdot n_{II}^{Car}} \cdot f_{Car} \tag{S32}$$

$$p_{Chl,II} = \frac{2c_{II} \cdot n_{II}^{Car}}{3c_I \cdot n_I^{Car} + 2c_{II} \cdot n_{II}^{Car}} \cdot f_{Car} \tag{S33}$$

where $f_{Chl:Car,meas}$ is the measured molar ratio of beta carotene to chlorophyll.

S1.6 Possible mechanisms of state transitions

We have implemented four possible mechanisms of state transitions.

PSII-quenching Model (default): In the presence of PQ_{red}, PSII reversibly enters a quenched state $PSII_q$. This increases the rate of overall excitation quenching as heat (k_H).

$$\frac{dPSII_q}{dt} = k_{Quench} \cdot (1 - PSII_q) \cdot PQ_{red} - \frac{k_{Unquench} \cdot PSII_q \cdot \ PQ_{red}^{\mathrm{n_{Unquench}}}}{\mathrm{K_{M,Unquench}^{n_{Unquench}}} + PQ_{red}^{\mathrm{n_{Unquench}}}} \tag{S34}$$

$$k_H = k_{H0} + k_{Hst} \cdot (PSII_q/\mathrm{PSII_{tot}}) \tag{S35}$$

Spillover: State 2 transition increases the fraction $spill(\leq spill_{max})$ of PSII excitations (E_{PSII}) passed to PSI.

$$\frac{dspill}{dt} = k_{spill} \cdot (spill_{max} - spill) \cdot PQ_{red} - k_{unspill} \cdot spill \cdot PQ_{ox} \tag{S36}$$

$$E_{PSII,spill} = E_{PSII} \cdot (1 - spill) \tag{S37}$$

$$E_{PSI,spill} = E_{PSI} + E_{PSII} \cdot spill \tag{S38}$$

PBS-Mobile: In state 2 transition, PBS move with rate v_{toPSI} from PSII ($PBS_{II} \geq PBS_{II,min}$) to PSI ($PBS_I \geq PBS_{I,min}$).

$$v_{toPSI} = k_{toPSI} \cdot (PBS_{II} - PBS_{II,min}) \cdot PQ_{red} - k_{toPSII} \cdot (PBS_I - PBS_{I,min}) \cdot PQ_{ox} \tag{S39}$$

$$\frac{dPBS_I}{dt} = -\frac{dPBS_{II}}{dt} = v_{toPSI} \tag{S40}$$

PBS-Detachment: In state 1 transition PBS detach equally from both photosystems (to $PBS_{free} \leq PBS_{free,max}$) with rate v_{detach}.

$$v_{detach} = k_{detach} \cdot (PBS_{free,max} - PBS_{free}) \cdot PQ_{ox} - k_{attach} \cdot PBS_{free} \cdot PQ_{red} \tag{S41}$$

$$\frac{dPBS_{free}}{dt} = -0.5\frac{dPBS_{II}}{dt} = -0.5\frac{dPBS_I}{dt} \tag{S42}$$

S1.7 Estimating pathway fluxes

We estimate the flux through the four main electron pathways from reactions that are preferably unique to the pathway. Because electrons from respiration also enter the Photosynthetic Electron Transport Chain (PETC), we scale the fluxes of LET and AEF, pathways that are defined beginning with PSII, with the fraction of PSII-derived electron flux

$$f_{PSII}^{influx} = \frac{2 \cdot vPSII}{2 \cdot vPSII + 2 \cdot vSDH + 2 \cdot vNDH2} \tag{S43}$$

All reaction fluxes are scaled by the number of involved electrons

$$vLET = 2 \cdot vFNR \cdot f_{PSII}^{influx} \tag{S44}$$

$$vCET = 2 \cdot vNDH1 \tag{S45}$$

$$vRET = 2 \cdot vSDH + 2 \cdot vNDH2 \tag{S46}$$

$$vAEF = (4 \cdot vFlv + 4 \cdot vCyd + 4 \cdot vCOX) \cdot f_{PSII}^{influx} \tag{S47}$$

We do not calculate these electron fluxes under very low light intensities ($< 10\,\mu mol(photons)\,m^{-2}\,s^{-1}$), where FNR flux can be reversed due to high NADPH concentrations stemming from respiration.

S1.8 Estimating fluorescence parameters and heat quenching

From the model, we calculated the fluorescence parameters Non-Photochemical Quenching (NPQ) and the effective quantum yield of PSII (Y(II)). Both parameters are estimated during a Saturation Pulse method (PAM-SP) experiment from the dark-adapted maximal fluorescence (F_m), the maximal fluorescence in the light (F'_m), and the steady-state fluorescence (F) as [43, 44]

$$NPQ = \frac{F_m - F'_m}{F'_m} \tag{S48}$$

$$Y(II) = \frac{F'_m - F}{F'_m} \tag{S49}$$

Since these quantities are defined on the basis of PSII chlorophyll fluorescence, we only consider the PSII fluorescence component F_{PSII} in these calculations. We simulate a saturation pulse (600 ms, 633 nm light, $15\,000\,\mu mol(photons)\,m^{-2}\,s^{-1}$) after oxidation of the PQ pool by 300 s of 700 nm monochromatic far-red light (yielding F_m) or following the steady-state simulation at a particular light intensity (F'_m).

S1.9 RuBisCO reactions

The cyanobacterial CBB is known to be redox-regulated, with thioredoxin being a possible regulator [32, 45]. As thioredoxin is reduced from the Fd pool, we defined a Fd-dependent CBB activity regulator CBB_a using Hill kinetics

$$\frac{dCBB_a}{dt} = \mathrm{k_{CBBa}} \cdot \left(Fd_{red}^{n_{CBB}} / (Fd_{red}^{n_{CBB}} + K_{Hill,CBB}^{n_{CBB}}) - CBB_a \right) \tag{S50}$$

Additionally, we modelled the CBB's dependency on ATP and NADPH with Michaelis-Menten (MM) kinetics. As CO_2 and O_2 compete for binding to RuBisCO, we modelled their influence as competitive inhibition.

$$v_{CBB} = v_{CBB,max} \cdot CBB_a \cdot \frac{ATP}{ATP + K_{M,ATP}} \cdot \frac{NADPH}{NADPH + K_{M,NADPH}} \cdot \frac{CO_2}{K_{M,CO_2}(1 + {O_2}/{K_{I,O_2}})} \tag{S51}$$

We model the O_2-dependent RuBisCO oxygenation reaction similarly. Since the oxygenation consumes ribulose-1,5-bisphosphate, the lumped stoichiometry requires CBB regeneration reactions. Therefore, we also impose the redox, ATP, and NADPH constraints. Additionally, we limit oxygenation at low concentration of organic carbon, i.e. 3PGA.

$$v_{Oxy} = v_{Oxy,max} \cdot CBB_a \cdot \frac{ATP}{ATP + K_{M,ATP}} \cdot \frac{NADPH}{NADPH + K_{M,NADPH}} \cdot \frac{O_2}{K_{M,O_2}(1 + {CO_2}/{K_{I,CO_2}})} \cdot \frac{3PGA}{3PGA + K_{M,3PGA}} \tag{S52}$$

S1.10 Flavodiiron proteins

Measurements of O_2 reduction have shown that Flv only reduces oxygen under high light intensities (Fig 2F). We replicate this behavior using Hill dependency on the likely limiting substrate, Fd:

$$v_{Flv} = \mathrm{k_{Flv}} \cdot O_2 \cdot H_O^+ \cdot Fd_{red}^{n_{Flv}} / (Fd_{red}^{n_{Flv}} + K_{Hill,Flv}^{n_{Flv}}) \tag{S53}$$

With H_O^+ being cytoplasmic protons used for O_2 reduction to water.

S1.11 The orange carotenoid protein

The Orange Carotenoid Protein (OCP) is known to be activated by its carotenoid cofactor absorbing light, which we calculate using the OCP absorption spectrum vector a_{OCP} [46]. We represent the inactivation of OCP by the fluorescence recovery protein through a MA kinetic. A maximum value OCP_{max} limits the amount of PBS excitations quenched.

$$\frac{dOCP}{dt} = (OCP_{max} - OCP) \cdot k_{OCPactivation} \cdot \text{simpson}(\text{diag}(I) \cdot a_{OCP}) \cdot lcf - OCP \cdot k_{OCPdeactivation} \quad \text{(S54)}$$

S1.12 Cellular import of CO_2

Cyanobacteria increase the carbon concentration around RuBisCO actively by the factor 100 - 1000 through the CCM and the construction of carboxysomes [5]. We model this by increasing the intracellular CO_2 partial pressure $p_{CO_2,in}$ by the factor $f_{CO_2,in}$:

$$p_{CO_2,in} = p_{CO_2,in} \cdot f_{CO_2,in} \quad \text{(S55)}$$

The concentration of CO_2 that can dissolve ($CO_{2,sol}$) in water is given by Henry's law [47]:

$$CO_{2,sol} = {}^{p_{CO_2,in}}/_{K_{CO_2}} \quad \text{(S56)}$$

$$\ln(K_{CO_2}) = -58.0931 + 90.5069\frac{100}{T} + 22.2940 \cdot \ln\left(\frac{T}{100}\right) + S\left(0.027766 - 0.025888 * \frac{T}{100} + 0.0050578(\frac{T}{100})^2\right) \quad \text{(S57)}$$

where the Henry constant K_{CO_2} depends on the temperature T and (intracellular) salinity S. Furthermore, CO_2 dissociates into ${HCO_3}^-$ and ${CO_3}^{2-}$ which are not substrates of RuBisCO. Therefore, we calculate the maximal usable CO_2 concentration $CO_{2,max}$ using the first dissociation constant K_1 [16]

$$CO_{2,max} = CO_{2,sol} \cdot \frac{H_O^+}{H_O^+ + K_1} \quad \text{(S58)}$$

$$pK_1 = -43.6977 - 0.0129037S + 1.364e - 4S^2 + \frac{2885.378}{T} + 7.045159 \cdot \ln(T) \quad \text{(S59)}$$

$$K_1 = 10^{-pK_1} \cdot \frac{1}{c_{Chl}} \quad \text{(S60)}$$

where K_1 is converted into $\text{mmol}\,\text{mol(chl)}^{-1}$. We describe the dynamic intracellular CO_2 concentration in fast equilibrium with $CO_{2,max}$:

$$\frac{dCO_2}{dt} = k_{CCM}(CO_{2,max} - CO_2) \quad \text{(S61)}$$

S2 Model parametrization

S2.1 Model parameters

A complete list of all parameters can be found in Tables A, D (strain-specific parameters), and E (for state transitions model).

The model depends on 98 parameters of which 37 were taken directly from literature (including 5 pigment absorption spectra), 9 rate parameters were estimated from direct or related literature rate measurements by determining an approximate rate through the pathway and dividing this rate by the assumed intracellular substrate concentrations, 6 parameters were physical quantities, 6 parameters were set according to the experimental irradiance, measuring light, O2 & CO2 condition, the concentration of cells, and temperature, 8 parameters were estimated from experimental data (PBS_PS2, PBS_PS1, PBS_free, PSItot, PSIItot, and pigment concentrations), 2 inhibition constants were estimated assuming they are equal to the Michaelis constants, the intracellular salinity S was assumed to be equal to seawater, intracellular buffering capacities of lumen and cytoplasm (bHo and bHi) were assumed constant [19], and 25 were fitted to different datasets or literature during model refinement:

- kUnquench, kQuench, KMUnquench, kOCPactivation, kOCPdeactivation, OCPmax, and PBS in fluo_influence were fitted to PAM-SP data [7] (Fig 3A),
- parameter vNQ_max was fit to 65% LET measured in cyanobacteria [48] (Fig 3E),
- parameter lcf was fit to 15 electrons PSI-1 s-1 of LET under 300 µmol m-2 s-1 light [48] (Fig 2A)
- parameter kCBBactivation was fit to activate the CBB within ca. one minute [27],
- parameter KMFdred was fit to have a low CBB activity in the dark but high activity at irradiances where respiration is inhibited (at irradiance above ca. 10 µmols m-2 s-1) [25],
- parameters KHillFdred and nHillFdred were fit to induce fast Flv activation at ca. 50 µmols m-2 s-1 white irradiance [25],
- parameter fCin was fit to CO2 assimilation data [49],
- parameters k_respiration and k_aa were fit to ca. 20% PQ reduction in dark [11],
- parameter k_Q was fit to a level allowing for PQ oxidation under blue irradiance [50],
- parameter KMPGA was fit to prohibit Photorespiration under very low concentration of CBB intermediates (represented by 3PGA),
- parameters KMNQ_Qox and KMNQ_Fdred were fit to produce continuous CET flow under high PQ reduction near light saturation [48],
- parameter kATPsynth was fit to produce sufficient ATP for supplying the CBB,
- parameter k_F1 was fit to provide a low fluorescence yield to PSI,
- parameter kNADHconsumption was fit to provide reasonable NADH/NAD+ ratios in darkness and light, and
- kATPconsumption and k_pass were fit to provide low capacity flows dissipating ATP and proton gradients.

The two weighing factors of PSI and PSII fluorescence in fluo_influence were fitted through the robustness analysis. The final model has been validated against the newly obtained data (added to Fig 3K and 3L, p. 9).

The parameter $fCin$, representing the intracellular to extracellular partial pressure ratio as increased by the carbon concentrating mechanism, was varied in the simulation between 100 and 1000 and has been set to 1000 based on the fit to the CO_2 fixation rates measured by Benschop *et al.* [49] (Fig 2H).

S2.2 Calculating model parameters from experimental data

In this work, we used measured pigment concentrations of the cyanobacterial samples as our input and we fit seven out of 23 parameters: Activation and inactivation rates of state transitions and OCP, the K_m of state transition activation by reduced PQ, and the fluorescence contribution of PBS, to PAM-SP measurements of our previous publication [7]. We estimated the photosystem concentrations and attachment of PBS from 77K fluorescence excitation-emission maps [7] (see Table D).

The 77K fluorescence ratio of PSI to PSII ($f_{I:II}^{Fluo}$) was found in the range of two to six, which is close to the physiological ratio [51]. Therefore we make the simplifying assumption that the fluorescence ratio is equal to the ratio of photosystem monomers. We first calculate the ratio of PSI to total photosystems (f_I^{PS}):

$$f_I^{PS} = \frac{1}{1 + {}^1/f_{I:II}^{Fluo}} \tag{S62}$$

We use this fraction to estimate how much cellular chlorophyll is bound in PSI (f_I^{Chl}) or PSII (f_{II}^{Chl}), assuming all chlorophyll is present in photosystems

$$f_I^{Chl} = 1 - f_{II}^{Chl} = \frac{f_I^{PS} \cdot n_I^{Chl}}{f_I^{PS} \cdot n_I^{Chl} + f_{II}^{PS} \cdot n_{II}^{Chl}} \tag{S63}$$

with the number of chlorophyll per PSI ($n_I^{Chl} = 96$) and PSII ($n_{II}^{Chl} = 35$). The inverse of the photosystems' chlorophyll content gives us the theoretical maximum chlorophyll-specific concentration. Thus we can calculate the concentration of PSI trimers (c_I) and PSII dimers (c_{II}) as:

$$\frac{1}{3}c_I = c_{I,mono} = f_I^{Chl} \cdot \frac{1000}{n_I^{Chl}} \tag{S64}$$

$$\frac{1}{2}c_{II} = c_{II,mono} = f_{II}^{Chl} \cdot \frac{1000}{n_{II}^{Chl}} \tag{S65}$$

To determine PBS association, we calculated the approximate 77K fluorescence signal from PBS associated with either PSI, PSII or free PBS (for details see [7]). We assumed that the signal of free PBS was ten times higher than those bound to photosystems (compare [52]).

S2.3 Calculating the mean light in a light absorbing culture

For simulations of experimental data where the culture density was provided, we further calculate the light encountered by a mean cell (I) for each wavelength according to an integrated Lambert-Beer function [53] accounting for the decreasing irradiance at various depths (L) due to cellular absorption. While simulating PAM-SP experiments we have assumed $L = 1$cm, corresponding to the diameter of the used measuring cuvette. We further estimate the pigment concentrations in the cuvette c_p. The resulting vector I has to take into account the culture's wavelength λ-specific absorption $a_{\lambda,tot}$ (normalized by volume), which we calculate from the absorption coefficients $a_{\lambda,p} \in A$ (normalized by chlorophyll, Eq (2)):

$$a_{\lambda,tot} = \sum_p a_{\lambda,p} \cdot c_p \tag{S66}$$

$$i_\lambda = \frac{1}{L}\int_0^L i_{\lambda,0} \cdot e^{l \cdot a_{\lambda,tot}} dl \quad \text{with } i_\lambda \in I,\ i_{\lambda,0} \in I_0 \tag{S67}$$

If no light attenuation is assumed $I = I_0$.

S2.4 Robustness analysis

To test the robustness of the model, we performed 10000 Monte Carlo simulations, where parameters were randomized around their default values. We calculated the residuals for each model and classified the models depending on the mean improvement of residuals and if all results could be improved simultaneously (S2 Fig). We additionally performed an automated fitting of the parameters to all residuals and compared the analysis results to those obtained with default parameters (S4 Fig).

S3 Additional model validation

S3.1 Dynamics of photoprotection

We have used our model to calculate fluorescence signal from cells grown under 633 nm. We have changed the input of the pigment composition and performed simulations using parameters fitted in Fig 3A and 3B.

S3.2 Internal PSII states

As discussed in the main text we calculated the fraction of open PSII for increasing light intensities and compared it to measurements [26] (S3 Fig). Although our response curve is less sensitive to increasing light, and PSII are still open for light intensities higher than reported 300 µmol(photons) m^{-2} s^{-1} we observe the expected exponential reduction of the fraction of open PSII with increasing light intensity.

S4 Additional analysis

S4.1 Mutant analysis

We extended our analysis of the impact of light intensity on the wild type and three mutants by additionally calculating their fraction of reduced pools, lumenal and stromal pH, and main fluxes (S5

Fig). We also simulated their NPQ and effective PSII quantum yield (S6 Fig).

S4.2 Activation of OCP in blue light

With our model analysis, we could identify the activation of OCP under blue light saturation pulses (S7 Fig).

S4.3 Metabolic Control Analysis (MCA)

Using metabolic control analysis (MCA), we quantified the control distribution in the system for different light intensities (e.g. S8, S9, and S10 Figs). We divided the set of all reactions into four non-overlapping sets: Light-driven electron flow: PSII, Cb_6f, PSI, NDH-1, ATPS, FNR; Respiration: Succinate Dehydrogenase (SDH), NAD(P)H Dehydrogenase complex 2 (NDH-2), "respiration" (sugar catabolism); RuBisCO reactions: CBB, Oxy; Terminal oxidases: Flv, Cyd (bb-type), COX (aa_3-type). For an example of MCA with differentiation of pathways, see Figs 4G and S9.

S4.4 Analysis of state transition models under light variation

We extended the analysis of state transition models by simulating the ensemble models under different light intensities (S11 Fig). We found that the alleviation of PQ reduction was associated with decreased carbon fixation under low light. Interestingly, the simulations of the spillover model at higher light intensities showed redox alleviation without slowing of the CBB. PSII quenching and PBS detachment did not affect PQ redox state or the CBB at high light while the PBS detachment model retained a wide range of steady-state redox states.

S4.5 Overexpression analysis

We reanalysed the MCA results and found that the flux control over the CBB by PSII, FNR, and Cb_6f was strongly dependent on light color and intensity (S12 Fig). Therefore, we analyzed the change in CBB rate in response to a two-fold increase ("overexpression") of the reaction's respective rate-determining parameter.

S4.6 Analysis of productivity by light-adapted cells

By substituting the pigment content of the default model with that of light-adapted cells, we could simulate their carbon fixation under various lights (S13 Fig). We saw that models adapted to red or blue light performed better than the default model (by default adapted to 633 nm light). Meanwhile, the performance in 450 to 550 nm light did not majorly improve.

References

[1] van Aalst M, Ebenhöh O, Matuszyńska A. Constructing and analysing dynamic models with modelbase v1.2.3: a software update. BMC Bioinformatics. 2021;22(1):1–15. doi:10.1186/s12859-021-04122-7.

[2] Moal G, Lagoutte B. Photo-induced electron transfer from photosystem I to NADP+: Characterization and tentative simulation of the in vivo environment. Biochimica et Biophysica Acta (BBA) - Bioenergetics. 2012;1817(9):1635–1645. doi:10.1016/J.BBABIO.2012.05.015.

[3] Keren N, Aurora R, Pakrasi HB. Critical Roles of Bacterioferritins in Iron Storage and Proliferation of Cyanobacteria. Plant Physiology. 2004;135(3):1666. doi:10.1104/PP.104.042770.

[4] Van De Meene AML, Sharp WP, McDaniel JH, Friedrich H, Vermaas WFJ, Roberson RW. Gross morphological changes in thylakoid membrane structure are associated with photosystem I deletion in Synechocystis sp. PCC 6803. Biochimica et Biophysica Acta (BBA) - Biomembranes. 2012;1818(5):1427–1434. doi:10.1016/J.BBAMEM.2012.01.019.

[5] Hagemann M, Song S, Brouwer EEM. Inorganic Carbon Assimilation in Cyanobacteria: Mechanisms, Regulation, and Engineering. Cyanobacteria Biotechnology. 2021; p. 1–31. doi:10.1002/9783527824908.CH1.

[6] Pogoryelov D, Reichen C, Klyszejko AL, Brunisholz R, Muller DJ, Dimroth P, et al. The oligomeric state of c rings from cyanobacterial F-ATP synthases varies from 13 to 15. Journal of bacteriology. 2007;189(16):5895–5902. doi:10.1128/JB.00581-07.

[7] Zavřel T, Segečová A, Kovács L, Lukeš M, Novák Z, Pohland AC, et al.. A comprehensive study of light quality acclimation in Synechocystis sp. PCC 6803; 2024. Available from: `https://www.biorxiv.org/content/10.1101/2023.06.08.544187v2`.

[8] Richardson AS. 2019 NRL Plasma Formulary. U.S. Naval Research Laboratory; 2019. Available from: `https://www.nrl.navy.mil/News-Media/Publications/NRL-Plasma-Formulary/`.

[9] Wieser ME. Atomic weights of the elements 2005 (IUPAC Technical Report). Pure and Applied Chemistry. 2006;78(11):2051–2066. doi:10.1351/pac200678112051.

[10] Matuszyńska A, Saadat NP, Ebenhöh O. Balancing energy supply during photosynthesis – a theoretical perspective. Physiologia Plantarum. 2019;166(1):392–402. doi:10.1111/PPL.12962.

[11] Khorobrykh S, Tsurumaki T, Tanaka K, Tyystjärvi T, Tyystjärvi E. Measurement of the redox state of the plastoquinone pool in cyanobacteria. FEBS Letters. 2020;594(2):367–375. doi:10.1002/1873-3468.13605.

[12] Zavřel T, Faizi M, Loureiro C, Poschmann G, Stühler K, Sinetova M, et al. Quantitative insights into the cyanobacterial cell economy. eLife. 2019;8. doi:10.7554/ELIFE.42508.

[13] Kauny J, Sétif P. NADPH fluorescence in the cyanobacterium Synechocystis sp. PCC 6803: A versatile probe for in vivo measurements of rates, yields and pools. Biochimica et Biophysica Acta (BBA) - Bioenergetics. 2014;1837(6):792–801. doi:10.1016/j.bbabio.2014.01.009.

[14] Tanaka K, Shimakawa G, Tabata H, Kusama S, Miyake C, Nakanishi S. Quantification of NAD(P)H in cyanobacterial cells by a phenol extraction method. Photosynthesis Research. 2021;148(1):57. doi:10.1007/S11120-021-00835-1.

[15] Doello S, Klotz A, Makowka A, Gutekunst K, Forchhammer K. A Specific Glycogen Mobilization Strategy Enables Rapid Awakening of Dormant Cyanobacteria from Chlorosis. Plant Physiology. 2018;177(2):594–603. doi:10.1104/PP.18.00297.

[16] Mojica Prieto FJ, Millero FJ. The values of pK1 + pK2 for the dissociation of carbonic acid in seawater. Geochimica et Cosmochimica Acta. 2002;66(14):2529–2540. doi:10.1016/S0016-7037(02)00855-4.

[17] Lewis CM, Flory JD, Moore TA, Moore AL, Rittmann BE, Vermaas WFJ, et al. Electrochemically Driven Photosynthetic Electron Transport in Cyanobacteria Lacking Photosystem II. Journal of the American Chemical Society. 2022;144(7):2933–2942. doi:10.1021/jacs.1c09291.

[18] Falkowski PG, Raven JA. Aquatic Photosynthesis. Princeton University Press; 2007. Available from: `https://www.degruyter.com/document/doi/10.1515/9781400849727/htmlhttps://bionumbers.hms.harvard.edu/bionumber.aspx?id=104567`.

[19] Ebenhöh O, Fucile G, Finazzi G, Rochaix JD, Goldschmidt-Clermont M. Short-term acclimation of the photosynthetic electron transfer chain to changing light: A mathematical model. Philosophical Transactions of the Royal Society B: Biological Sciences. 2014;369(1640):20130223. doi:10.1098/rstb.2013.0223.

[20] Bernát G, Waschewski N, Rögner M. Towards efficient hydrogen production: The impact of antenna size and external factors on electron transport dynamics in Synechocystis PCC 6803. Photosynthesis Research. 2009;99(3):205–216. doi:10.1007/s11120-008-9398-7.

[21] Sétif P, Shimakawa G, Krieger-Liszkay A, Miyake C. Identification of the electron donor to flavodiiron proteins in Synechocystis sp. PCC 6803 by in vivo spectroscopy. Biochimica et Biophysica Acta (BBA) - Bioenergetics. 2020;1861(10):148256. doi:10.1016/J.BBABIO.2020.148256.

[22] Cooley JW, Vermaas WFJ. Succinate Dehydrogenase and Other Respiratory Pathways in Thylakoid Membranes of Synechocystis sp. Strain PCC 6803: Capacity Comparisons and Physiological Function. Journal of Bacteriology. 2001;183(14):4251–4258. doi:10.1128/JB.183.14.4251-4258.2001.

[23] Ermakova M, Huokko T, Richaud P, Bersanini L, Howe CJ, Lea-Smith DJ, et al. Distinguishing the Roles of Thylakoid Respiratory Terminal Oxidases in the Cyanobacterium Synechocystis sp. PCC 6803. Plant Physiology. 2016;171(2):1307–1319. doi:10.1104/PP.16.00479.

[24] Kihara S, Hartzler DA, Savikhin S. Oxygen concentration inside a functioning photosynthetic cell. Biophysical Journal. 2014;106(9):1882–1889. doi:10.1016/j.bpj.2014.03.031.

[25] Schuurmans RM, van Alphen P, Schuurmans JM, Matthijs HCP, Hellingwerf KJ. Comparison of the Photosynthetic Yield of Cyanobacteria and Green Algae: Different Methods Give Different Answers. PLOS ONE. 2015;10(9):e0139061. doi:10.1371/journal.pone.0139061.

[26] Miller NT, Vaughn MD, Burnap RL. Electron flow through NDH-1 complexes is the major driver of cyclic electron flow-dependent proton pumping in cyanobacteria. Biochimica et Biophysica Acta - Bioenergetics. 2021;1862(3). doi:10.1016/j.bbabio.2020.148354.

[27] Nikkanen L, Santana Sánchez A, Ermakova M, Rögner M, Cournac L, Allahverdiyeva Y. Functional redundancy between flavodiiron proteins and NDH-1 in Synechocystis sp. PCC 6803. The Plant Journal. 2020;103(4):1460–1476. doi:10.1111/TPJ.14812.

[28] Tian L, van Stokkum IHM, Koehorst RBM, Jongerius A, Kirilovsky D, van Amerongen H. Site, Rate, and Mechanism of Photoprotective Quenching in Cyanobacteria. Journal of the American Chemical Society. 2011;133(45):18304–18311. doi:10.1021/ja206414m.

[29] Zavřel T, Očenášová P, Červený J. Phenotypic characterization of Synechocystis sp. PCC 6803 substrains reveals differences in sensitivity to abiotic stress. PLoS ONE. 2017;12(12). doi:10.1371/JOURNAL.PONE.0189130.

[30] Savir Y, Noor E, Milo R, Tlusty T. Cross-species analysis traces adaptation of Rubisco toward optimality in a low-dimensional landscape. Proceedings of the National Academy of Sciences. 2010;107(8):3475–3480. doi:10.1073/PNAS.0911663107.

[31] Wadano A, Nishikawa K, Hirahashi T, Satoh R, Iwaki T. Reaction mechanism of phosphoribulokinase from a cyanobacterium, Synechococcus PCC7942. Photosynthesis Research. 1998;56(1):27–33. doi:10.1023/A:1005979801741.

[32] Tsukamoto Y, Fukushima Y, Hara S, Hisabori T. Redox Control of the Activity of Phosphoglycerate Kinase in Synechocystis sp. PCC6803. Plant and Cell Physiology. 2013;54(4):484–491. doi:10.1093/PCP/PCT002.

[33] Koksharova O, Schubert M, Shestakov S, Cerff R. Genetic and biochemical evidence for distinct key functions of two highly divergent GAPDH genes in catabolic and anabolic carbon flow of the cyanobacterium Synechocystis sp. PCC 6803. Plant Molecular Biology. 1998;36:183.

[34] Huege J, Goetze J, Schwarz D, Bauwe H, Hagemann M, Kopka J. Modulation of the Major Paths of Carbon in Photorespiratory Mutants of Synechocystis. PLOS ONE. 2011;6(1):e16278. doi:10.1371/JOURNAL.PONE.0016278.

[35] Ranjbar Choubeh R, Wientjes E, Struik PC, Kirilovsky D, van Amerongen H. State transitions in the cyanobacterium Synechococcus elongatus 7942 involve reversible quenching of the photosystem II core. Biochimica et Biophysica Acta (BBA) - Bioenergetics. 2018;1859(10):1059–1066. doi:10.1016/J.BBABIO.2018.06.008.

[36] Schreiber U. Redox changes of ferredoxin, P700, and plastocyanin measured simultaneously in intact leaves. Photosynthesis Research. 2017;134(3):343–360. doi:10.1007/S11120-017-0394-7/FIGURES/11.

[37] Li YH, Tsui TF. The solubility of CO 2 in water and sea water . Journal of Geophysical Research. 1971;76(18):4203–4207. doi:10.1029/jc076i018p04203.

[38] Belkin S, Mehlhorn RJ, Packer L. Proton Gradients in Intact Cyanobacteria. Plant Physiology. 1987;84(1):25–30. doi:10.1104/pp.84.1.25.

[39] Rodrigues JS, Kovács L, Lukeš M, Höper R, Steuer R, Červený J, et al. Characterizing isoprene production in cyanobacteria – Insights into the effects of light, temperature, and isoprene on Synechocystis sp. PCC 6803. Bioresource Technology. 2023;380:129068. doi:10.1016/j.biortech.2023.129068.

[40] Liebermeister W, Uhlendorf J, Klipp E. Modular rate laws for enzymatic reactions: thermodynamics, elasticities and implementation. Bioinformatics. 2010;26(12):1528–1534. doi:10.1093/bioinformatics/btq141.

[41] Noor E, Flamholz A, Liebermeister W, Bar-Even A, Milo R. A note on the kinetics of enzyme action: A decomposition that highlights thermodynamic effects. FEBS Letters. 2013;587(17):2772–2777. doi:10.1016/j.febslet.2013.07.028.

[42] Fuente D, Lazar D, Oliver-Villanueva JV, Urchueguía JF. Reconstruction of the absorption spectrum of Synechocystis sp. PCC 6803 optical mutants from the in vivo signature of individual pigments. Photosynthesis Research. 2021;147(1):75–90. doi:10.1007/s11120-020-00799-8.

[43] Schreiber U. Pulse-Amplitude-Modulation (PAM) Fluorometry and Saturation Pulse Method: An Overview. In: Chlorophyll a Fluorescence. January 2004. Dordrecht: Springer Netherlands; 2004. p. 279–319. Available from: http://link.springer.com/10.1007/978-1-4020-3218-9_11.

[44] Schreiber U, Klughammer C, Schansker G. Rapidly Reversible Chlorophyll Fluorescence Quenching Induced by Pulses of Supersaturating Light in Vivo. Photosynthesis Research. 2019;142(1):35–50. doi:10.1007/s11120-019-00644-7.

[45] Michelet L, Zaffagnini M, Morisse S, Sparla F, Pérez-Pérez ME, Francia F, et al. Redox regulation of the Calvin–Benson cycle: something old, something new. Frontiers in Plant Science. 2013;4:470. doi:10.3389/fpls.2013.00470.

[46] Wilson A, Punginelli C, Gall A, Bonetti C, Alexandre M, Routaboul JM, et al. A photoactive carotenoid protein acting as light intensity sensor. Proceedings of the National Academy of Sciences. 2008;105(33):12075–12080. doi:10.1073/PNAS.0804636105.

[47] König M, Vaes J, Klemm E, Pant D. Solvents and Supporting Electrolytes in the Electrocatalytic Reduction of CO2. iScience. 2019;19:135–160. doi:10.1016/j.isci.2019.07.014.

[48] Theune ML, Hildebrandt S, Steffen-Heins A, Bilger W, Gutekunst K, Appel J. In-vivo quantification of electron flow through photosystem I – Cyclic electron transport makes up about 35% in a cyanobacterium. Biochimica et Biophysica Acta - Bioenergetics. 2021;1862(3):148353. doi:10.1016/j.bbabio.2020.148353.

[49] Benschop JJ, Badger MR, Dean Price G. Characterisation of CO2 and HCO3- uptake in the cyanobacterium Synechocystis sp. PCC6803. Photosynthesis Research. 2003;77(2):117–126. doi:10.1023/A:1025850230977.

[50] Kirilovsky D, Kaňa R, Prášil O. Mechanisms Modulating Energy Arriving at Reaction Centers in Cyanobacteria. In: Demmig-Adams B, Garab G, Adams III W, Govindjee, editors. Non-Photochemical Quenching and Energy Dissipation in Plants, Algae and Cyanobacteria. Dordrecht: Springer Netherlands; 2014. p. 471–501. Available from: https://link.springer.com/10.1007/978-94-017-9032-1_22.

[51] Antal TK, Kovalenko IB, Rubin AB, Tyystjärvi E. Photosynthesis-Related Quantities for Education and Modeling. Photosynthesis Research. 2013;117(1-3):1–30. doi:10.1007/s11120-013-9945-8.

[52] Acuña AM, Snellenburg JJ, Gwizdala M, Kirilovsky D, van Grondelle R, van Stokkum IHM. Resolving the contribution of the uncoupled phycobilisomes to cyanobacterial pulse-amplitude modulated (PAM) fluorometry signals. Photosynthesis Research. 2016;127(1):91–102. doi:10.1007/s11120-015-0141-x.

[53] Pfaffinger CE, Schöne D, Trunz S, Löwe H, Weuster-Botz D. Model-Based Optimization of Microalgae Areal Productivity in Flat-Plate Gas-Lift Photobioreactors. Algal Research. 2016;20:153–163. doi:10.1016/j.algal.2016.10.002.
