# Supplementary figures and images for: Shedding light on blue-green photosynthesis: A wavelength-dependent mathematical model of photosynthesis in Synechocystis sp. PCC 6803
Source: PLoS Comput Biol. 2024 Sep 12;20(9):e1012445. doi: 10.1371/journal.pcbi.1012445 (PMC11421815; doi:10.1371/journal.pcbi.1012445)

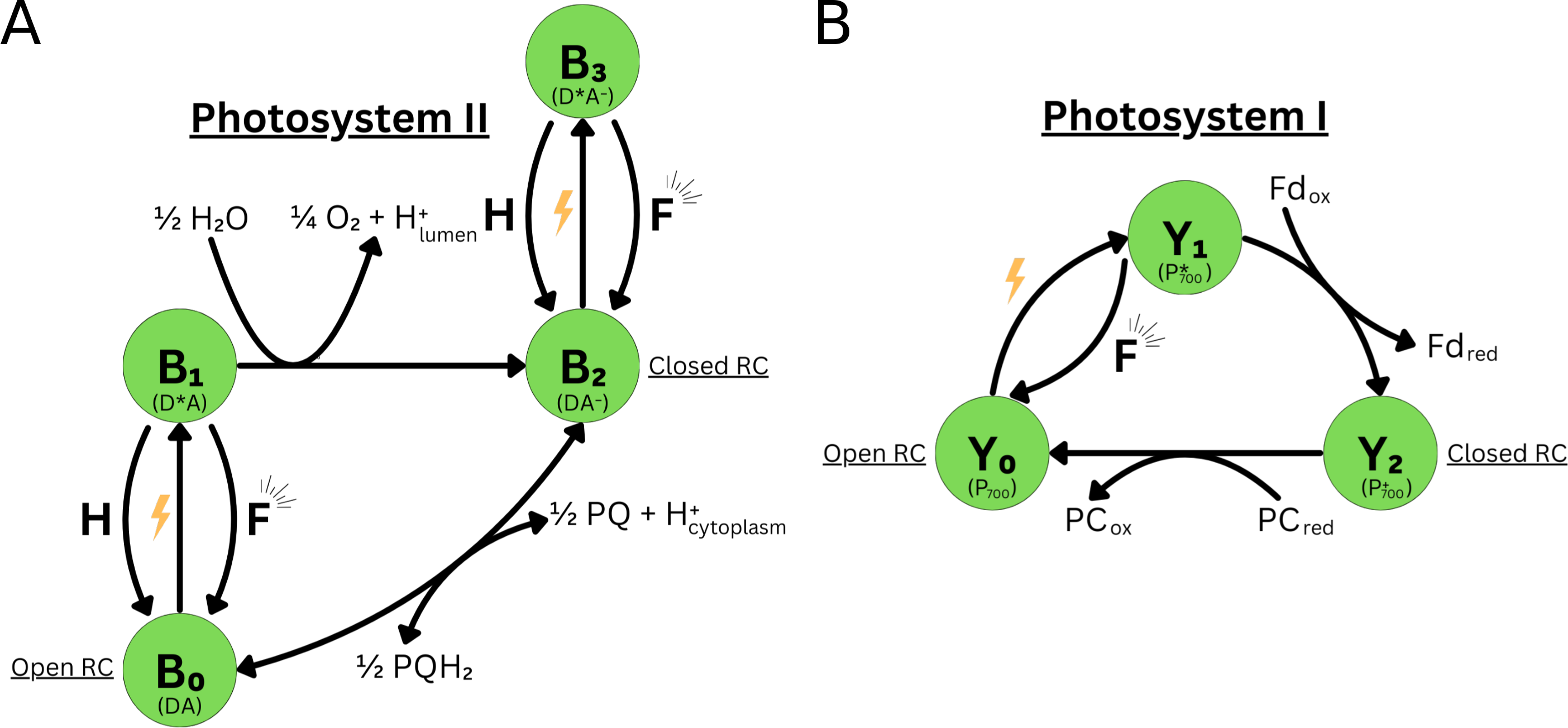

Supplement: S1 Fig — A: Photosystem II. The open reaction centers (RC) B0 are excited by light (yellow bolt). The excited state B1 can relax to B0 by heat (H) and fluorescence (F) emission or perform photochemistry. The latter promotes the RC to the closed state B2 and extracts one electron from water. Excitation of B2 can only be quenched as H or F. Lastly, B2 can reduce Plastoquinone (PQ) and enter the open state B0 again. Parentheses show the assumed state of the special pair chlorophyll P680 (D) and electron acceptor plastoquinone A (A): excited (*) and reduced(−). B: Photosystem I. Light excites the open reaction centers Y0. The excited Y1 state can perform photochemistry by reducing Ferredoxin (Fd) and becoming oxidized to Y2. We also consider a minor relaxation of Y1 to Y0 through F. The oxidized Y2 is reduced by Plastocyanin (PC). Parentheses show the assumed state of the reaction center P700. (TIF) [file pcbi.1012445.s002.tif]

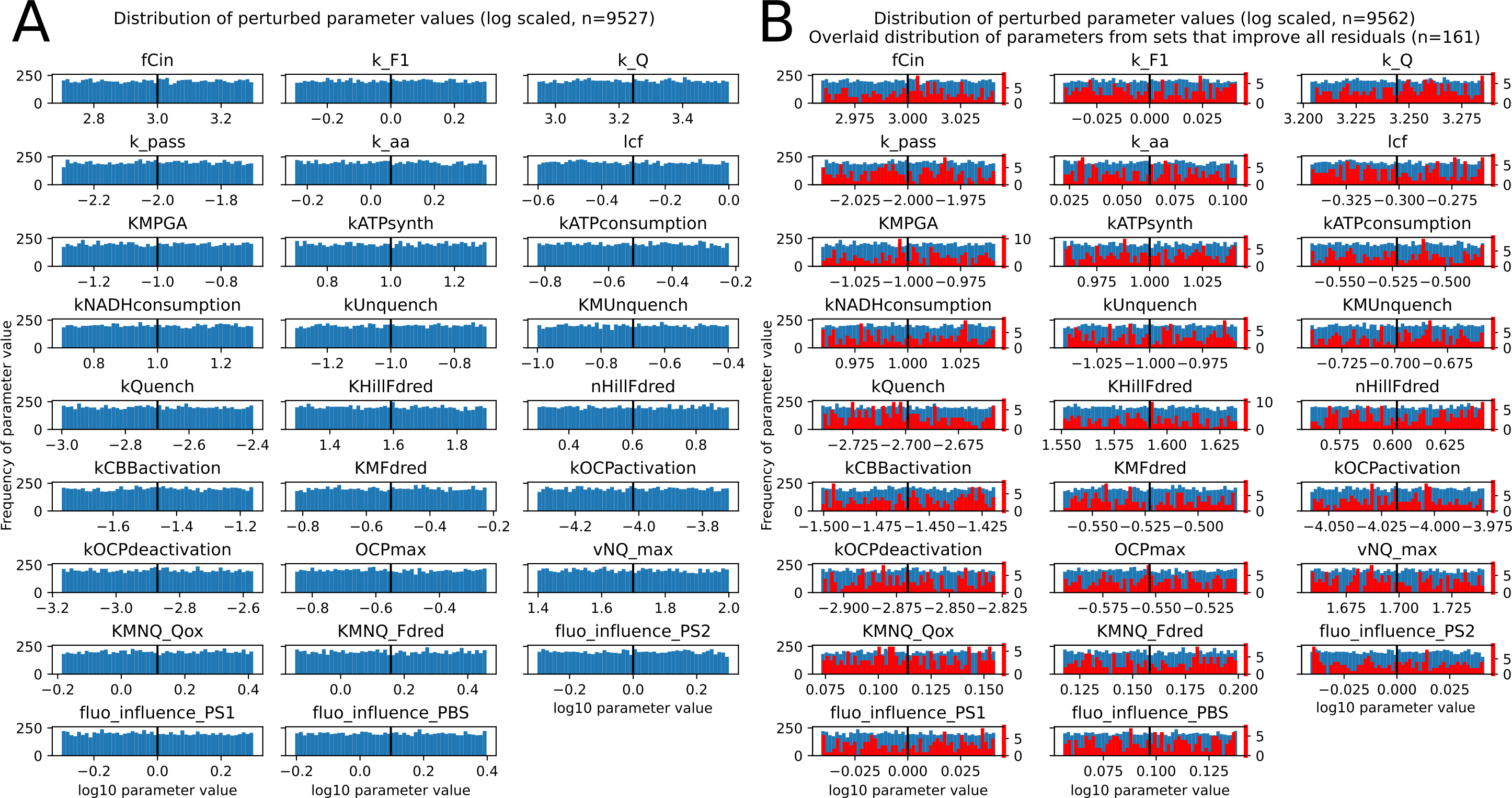

Supplement: S2 Fig — We performed 10,000 simulations of the model with a subset of parameters being randomized: The 24 parameters marked as “manually fitted” in Table A in S1 Appendix were randomly varied within ±factor 2 (A) or ±10% (B). We drew independent randomization factors for each varied parameter in each model from a log-uniform distribution. We show the distribution of log-transformed parameter values used in the Monte Carlo simulations. 1.6% of simulations in B showed an improvement in all residual functions and a distribution of their parameters is overlaid in red. The red histogram is rescaled for visibility. (TIF) [file pcbi.1012445.s003.tif]

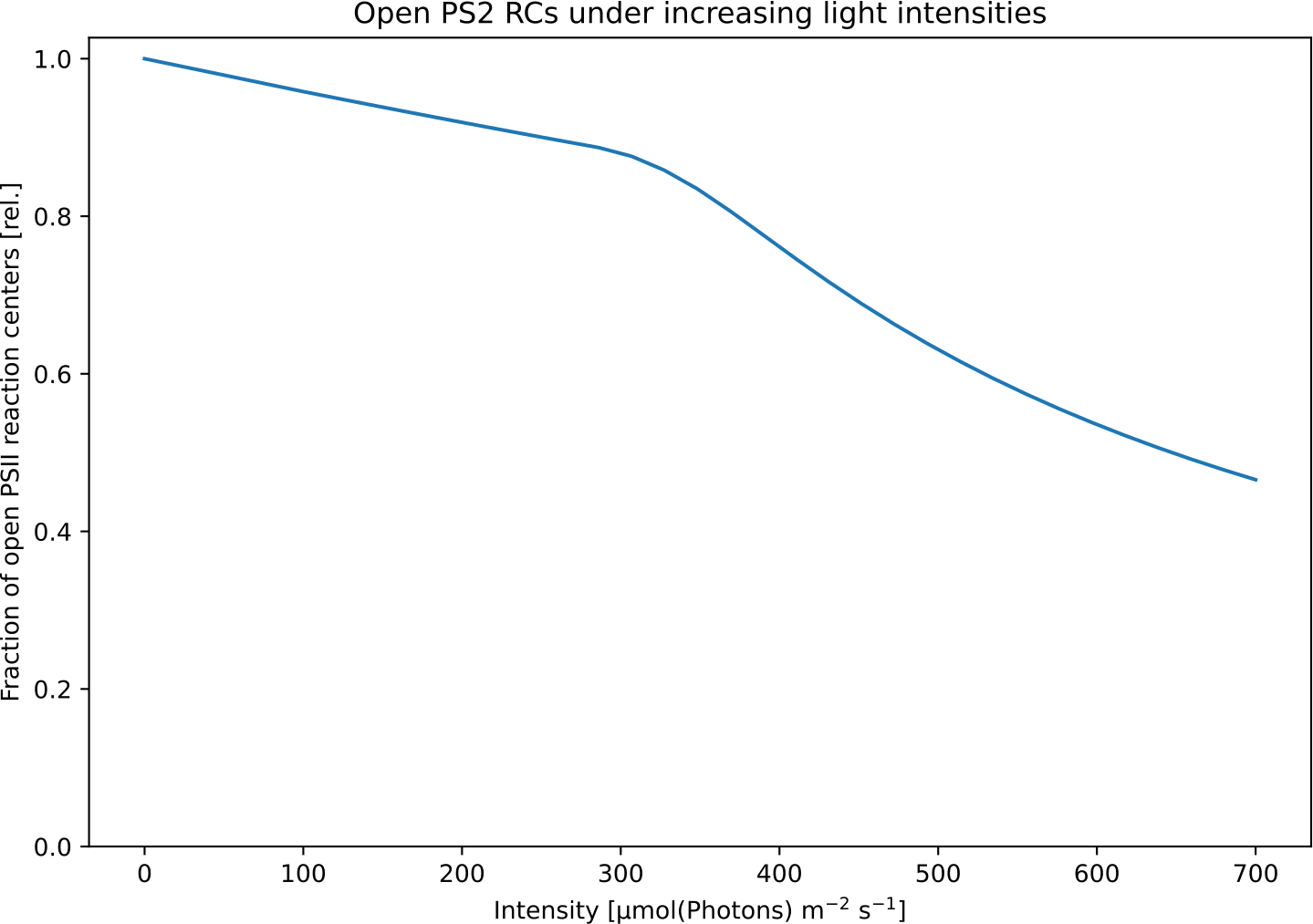

Supplement: S3 Fig — The model was simulated to steady-state under illumination with a fluorescence lamp spectrum at intensities between 0.1 and 700 μmol(photons) m−2 s−1. The open fraction was calculated as the fraction of PSII in non-reduced states B0 and B1 [50] (see S1 Fig). (TIF) [file pcbi.1012445.s004.tif]

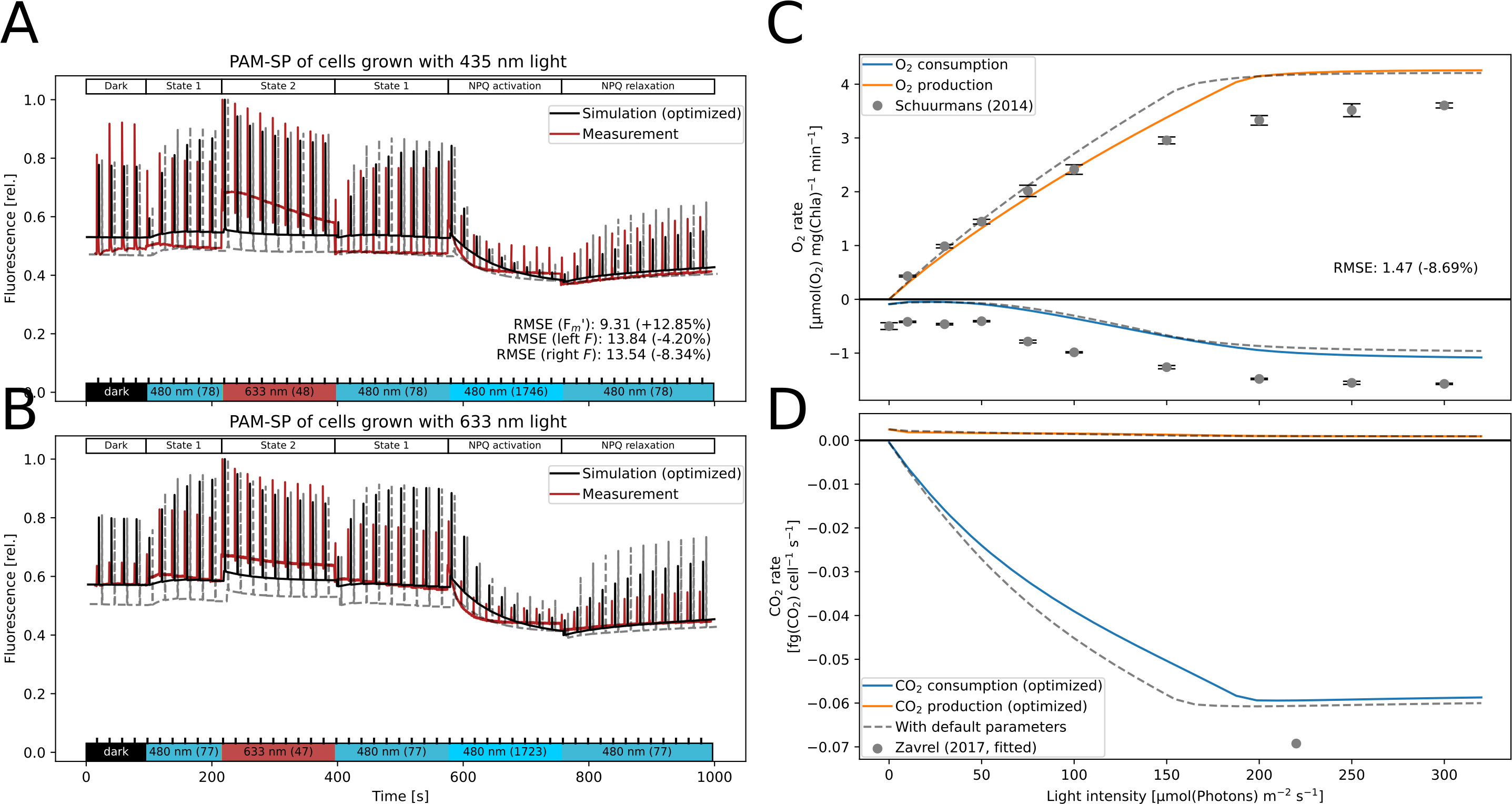

Supplement: S4 Fig — A,B: Repetition of Fig 3A and 3B. C,D: Repetition of Fig 2F and 2G. Solid lines show simulations using the optimized parameter set. For comparison, the default simulations are shown with dashed lines. We optimized the parameter set by minimizing the mean of all residual functions including validation data. Additionally, we penalized the optimization score if any residuals worsened compared to the simulation with default parameters. The simulations with optimized parameters moderately improved the fit of the data but did not show new behavior or features. RMSE quantifies the residuals of the respective simulation with optimized parameters. The difference to the residuals of a model with default parameters is in parentheses. (TIF) [file pcbi.1012445.s005.tif]

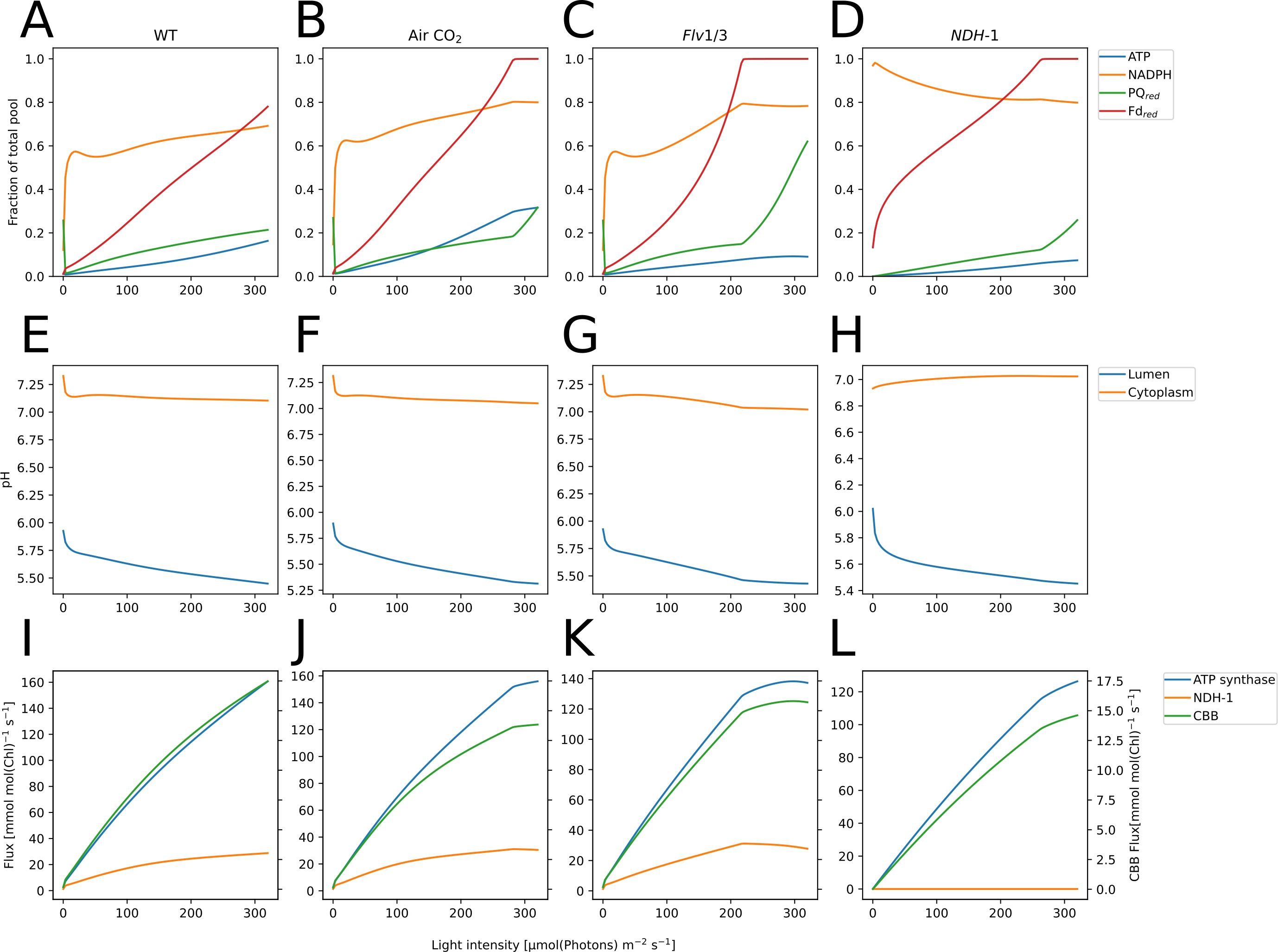

Supplement: S5 Fig — A-D: Per column, the models represent the wild type (WT) in saturating CO2 (A) and ambient air CO2 (400 ppm, B), a flavodiiron (Flv1/3) knockout mutant (C) and NAD(P)H Dehydrogenase-like complex 1 (NDH-1) knockout mutant (D). The levels and production fluxes of ATP and NADPH are shown as the primary output metabolites of photosynthesis, next to central redox carriers. The CBB flux in the last row is rescaled to the rightward axis for better visibility. (TIF) [file pcbi.1012445.s006.tif]

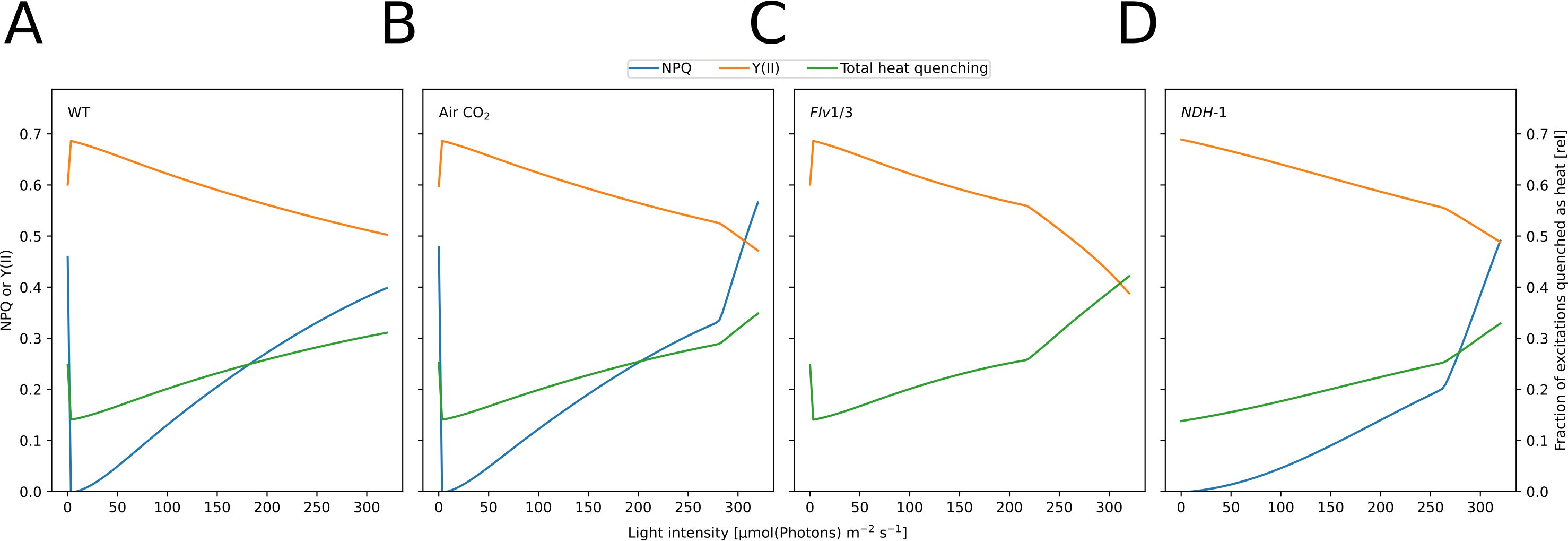

Supplement: S6 Fig — A-D: The models represent the wild type (WT) in saturating CO2 (A) and ambient air CO2 (400 ppm, B), a flavodiiron (Flv1/3) knockout mutant (C) and NAD(P)H Dehydrogenase-like complex 1 (NDH-1) knockout mutant (D). The electron fluxes were calculated according to section S1.7 in S1 Appendix. The models were simulated to steady state for light intensities between 0.1 μmol(photons) m−2 s−1 and 300 μmol(photons) m−2 s−1. Modeled conditions as in Fig 2A–2D. (TIF) [file pcbi.1012445.s007.tif]

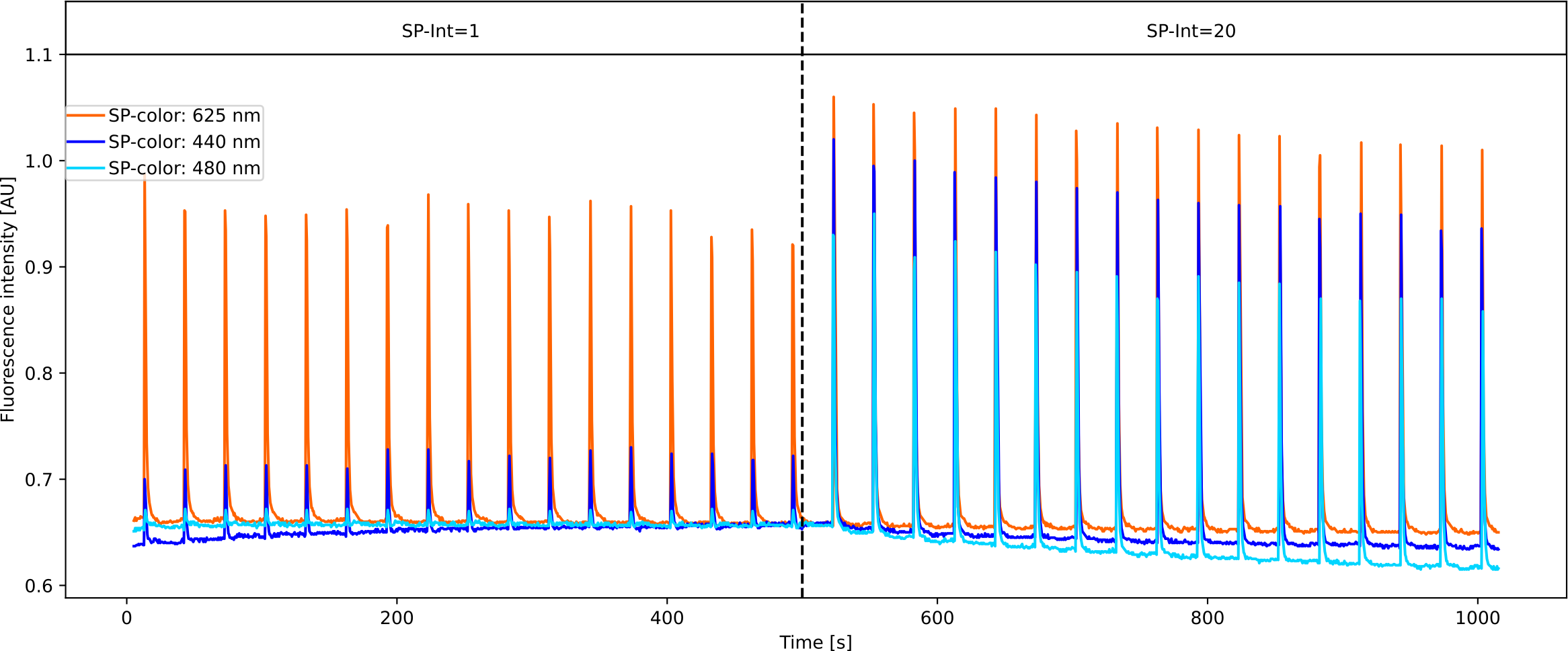

Supplement: S7 Fig — The measurements were performed with Multi-Color PAM (Walz, Effeltrich, Germany). Low-intensity pulses (SP-Int = 1) affect the steady-state fluorescence (F) only weakly. With each pulse of 440 nm and 480 nm light, however, the F level decreases stepwise, pointing at fluorescence quenching, possibly through Orange Carotenoid Protein (OCP). The culture of Synechocystis sp. PCC 6803 was pre-cultivated in a conical flask on a shaker under cool white light (30 μmol(photons) m−2 s−1) at 23°C to OD750 = 0.2 (measured with Shimadzu UV-Vis 2600 spectrophotometer, Shimadzu, Kyoto, Japan). For the measurement, 1.5 mL culture was transferred to a quartz cuvette and dark-acclimated for 5 min prior to each measurement. During the measurement, a custom-made protocol was used with the following settings: Analysis mode: SP analysis; AL off; SP-int = 1 (500 s) / 20 (500 s); SP-color = 440 nm / 480 nm / 625 nm; ML-color = 625 nm. (TIF) [file pcbi.1012445.s008.tif]

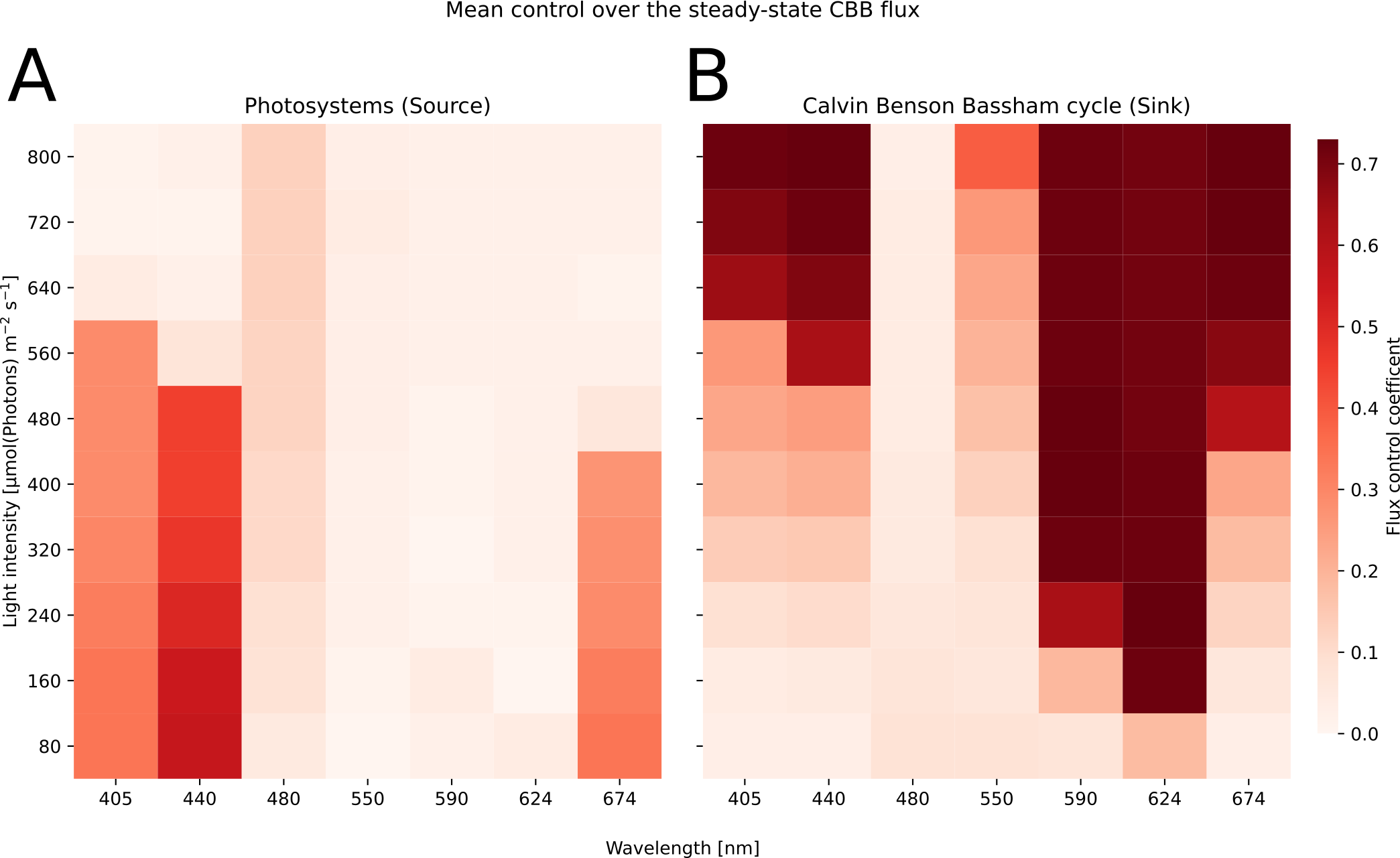

Supplement: S8 Fig — We simulated the model under the lights in Fig 5A with a range of intensities from 80 to 800 μmol(photons) m−2 s−1 to steady-state. By varying the photosystem concentrations (A) and the maximal rate of the CBB (B) by ± 1%, we quantified their control on the CBB flux. Plots show the absolute control coefficients. The left graph shows the mean of both photosystems. Higher values signify stronger pathway control. (TIF) [file pcbi.1012445.s009.tif]

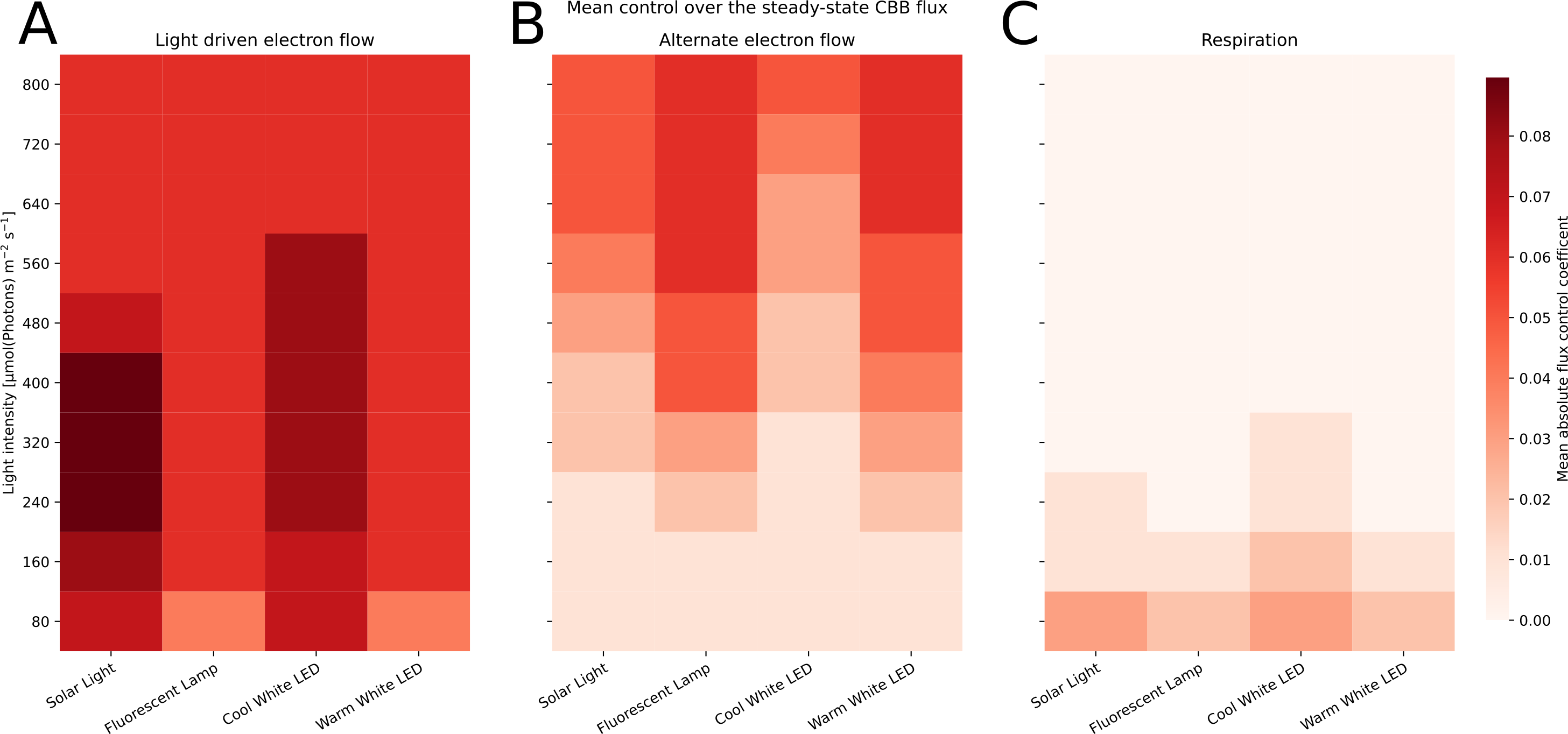

Supplement: S9 Fig — We simulated the model to steady-state with the lights in Fig 4A–4D at a range of intensities from 80 to 800 μmol(photons) m−2 s−1. By varying the protein concentration, maximal velocity, or rate constant of a reaction by ± 1%, we quantified their control on the CBB flux by calculating flux control coefficients. We display the absolutes of control coefficients as means within the following electron pathways: light-driven (A; PSI, PSII, Cytochrome b6f complex, NDH-1, FNR), alternate (B; Flv, Cytochrome bd quinol oxidase (Cyd), Cytochrome c oxidase), and respiration (C; lumped respiration, Succinate Dehydrogenase, NDH-2). Higher values signify stronger pathway control. (TIF) [file pcbi.1012445.s010.tif]

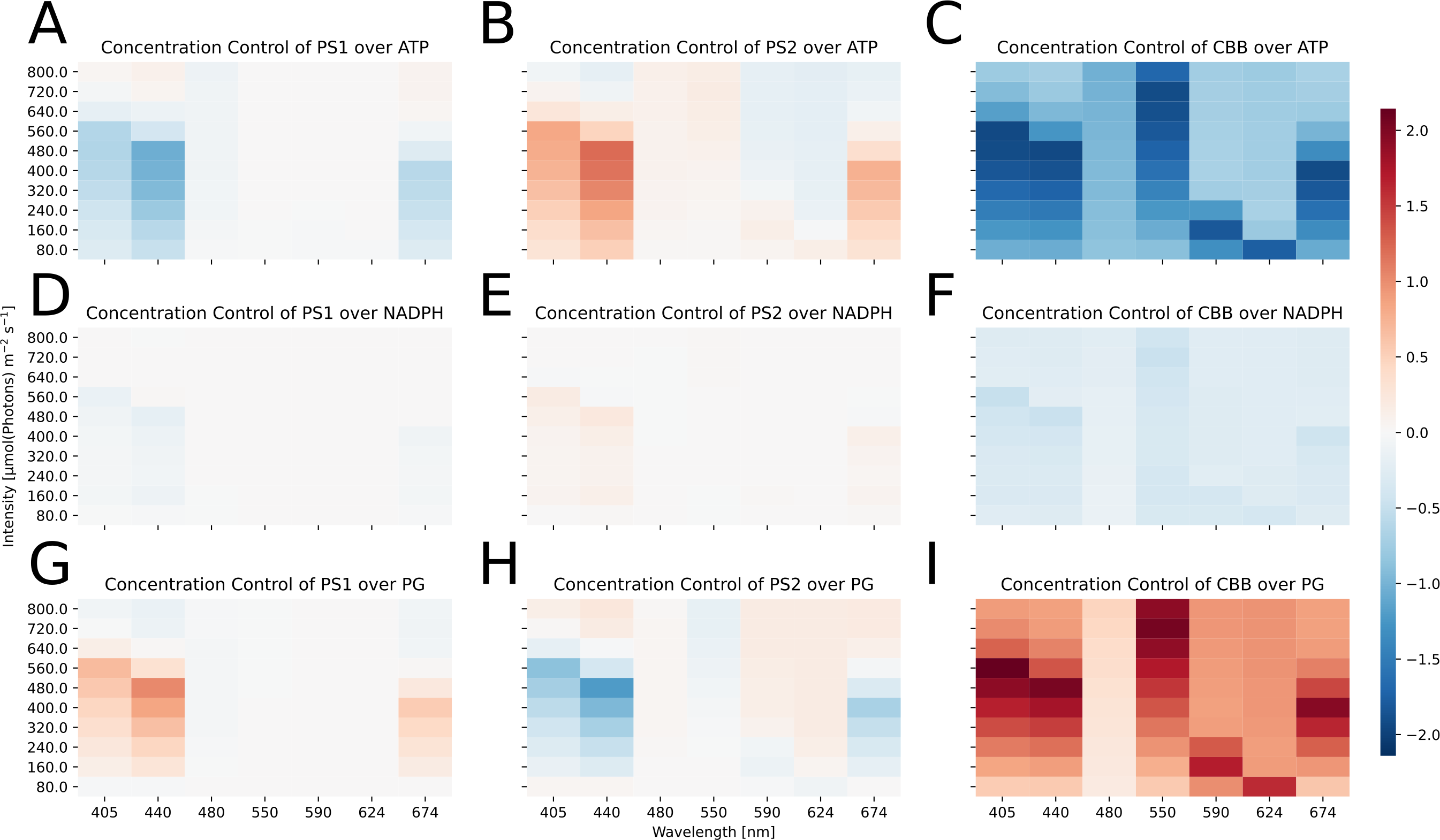

Supplement: S10 Fig — Per column, we investigated the control of PSI (A), PSII (B), and CBB (C). We simulated the model under the lights in Fig 5A with a range of intensities from 80 to 800 μmol(photons) m−2 s−1 to steady-state. By varying the photosystem concentrations and the maximal rate of the CBB by ± 1%, we quantified their control on the metabolite concentrations. More positive/negative values signify a stronger positive/negative control of the respective PETC component. The photosystems control is generally highest under illumination within the chlorophyll absorption spectrum (405 nm, 440 nm and 674 nm), with PSII also having control in the red spectrum. The CBB has a generally high control until a critical light intensity is reached. Increasing PSI or CBB flux generally lowers ATP and NADPH concentrations and increased 2PG, PSII has the opposite effect. At high light, some control relationships become inverted. (TIF) [file pcbi.1012445.s011.tif]

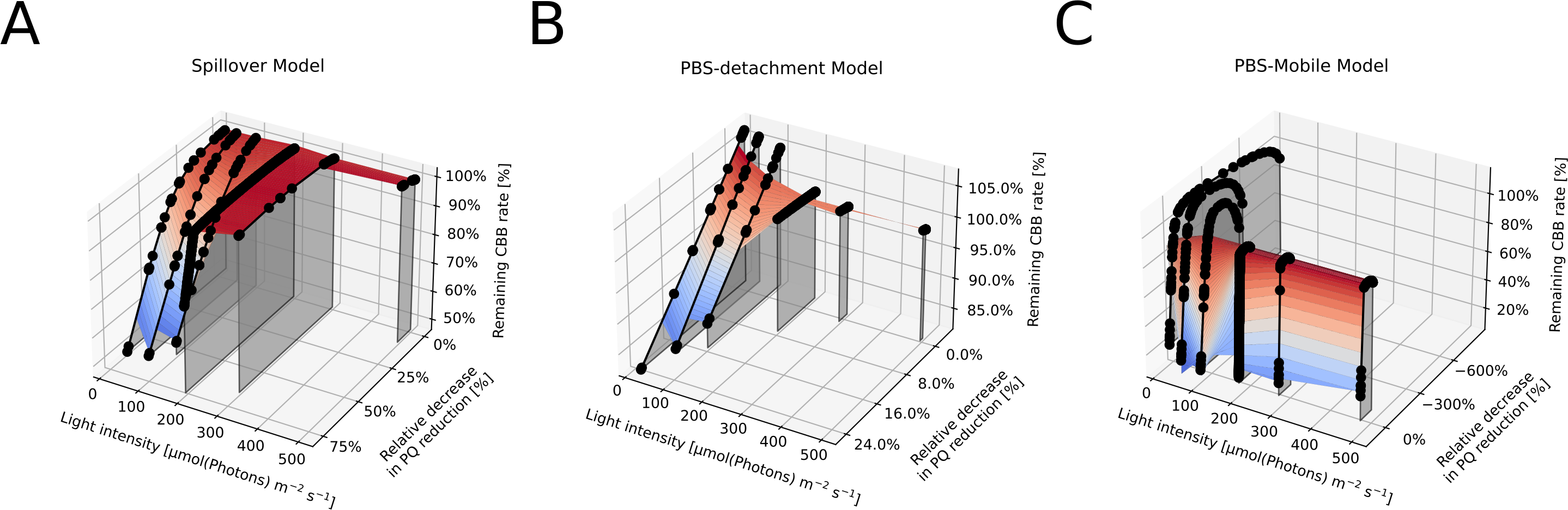

Supplement: S11 Fig — A-C: Simulations for the Spillover Model (A), PBS-detachment Model (B), and the PBS-Mobile model (C). Each point shows the result of a model run with varied parameters. The models were simulated for different light intensities (x-axis), and we calculated how the activation of the state transition mechanism affected the reduction of the PQ pool (y-axis) and the rate of the CBB (z-axis). For higher light intensities, the state transition has less effect on the PQ pool and CBB rate. Our implementations of the PBS detachment and mobile models can both lead to an additional reduction of the PQ pool. The rate of the CBB generally decreased with alleviation of the PQ redox state but also decreased under strong overreduction (see C). Only the PBS-mobile model simulates a decrease in PQ redox state under high light. (TIF) [file pcbi.1012445.s012.tif]

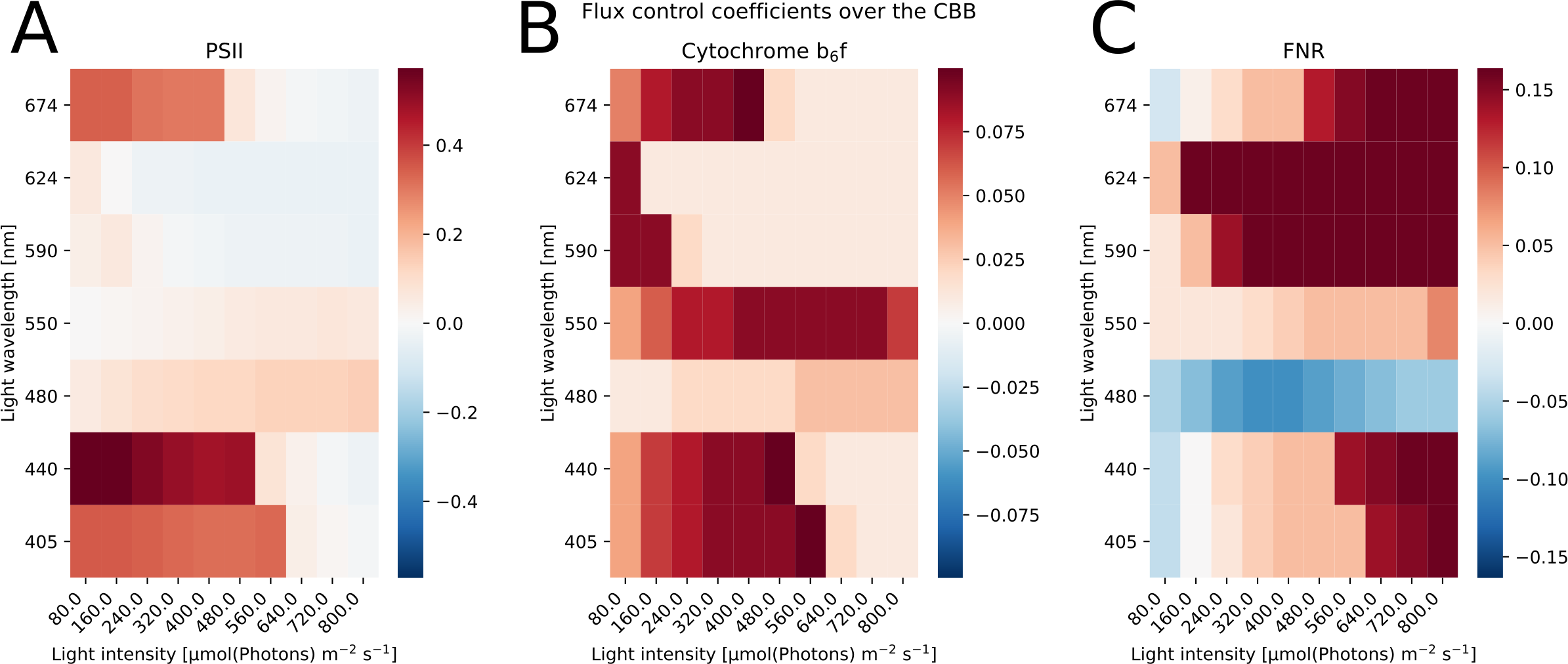

Supplement: S12 Fig — We show the flux control coefficients of PSII (A), FNR (B), and Cytochrome b6f complex (C) under light variation. We simulated the model to steady-state using the lights in Fig 5A with a range of intensities from 80 to 800 μmol(photons) m−2 s−1. By varying the protein concentration, maximal velocity, or rate constant of a reaction by ± 1%, we quantified their control on the CBB flux by calculating flux control coefficients. Values above zero show a positive effect of increasing the reaction rate and values below zero show a negative effect. We selected these reactions for analysis Fig 8 because their control coefficients strongly varied between light intensities and colors. (TIF) [file pcbi.1012445.s013.tif]

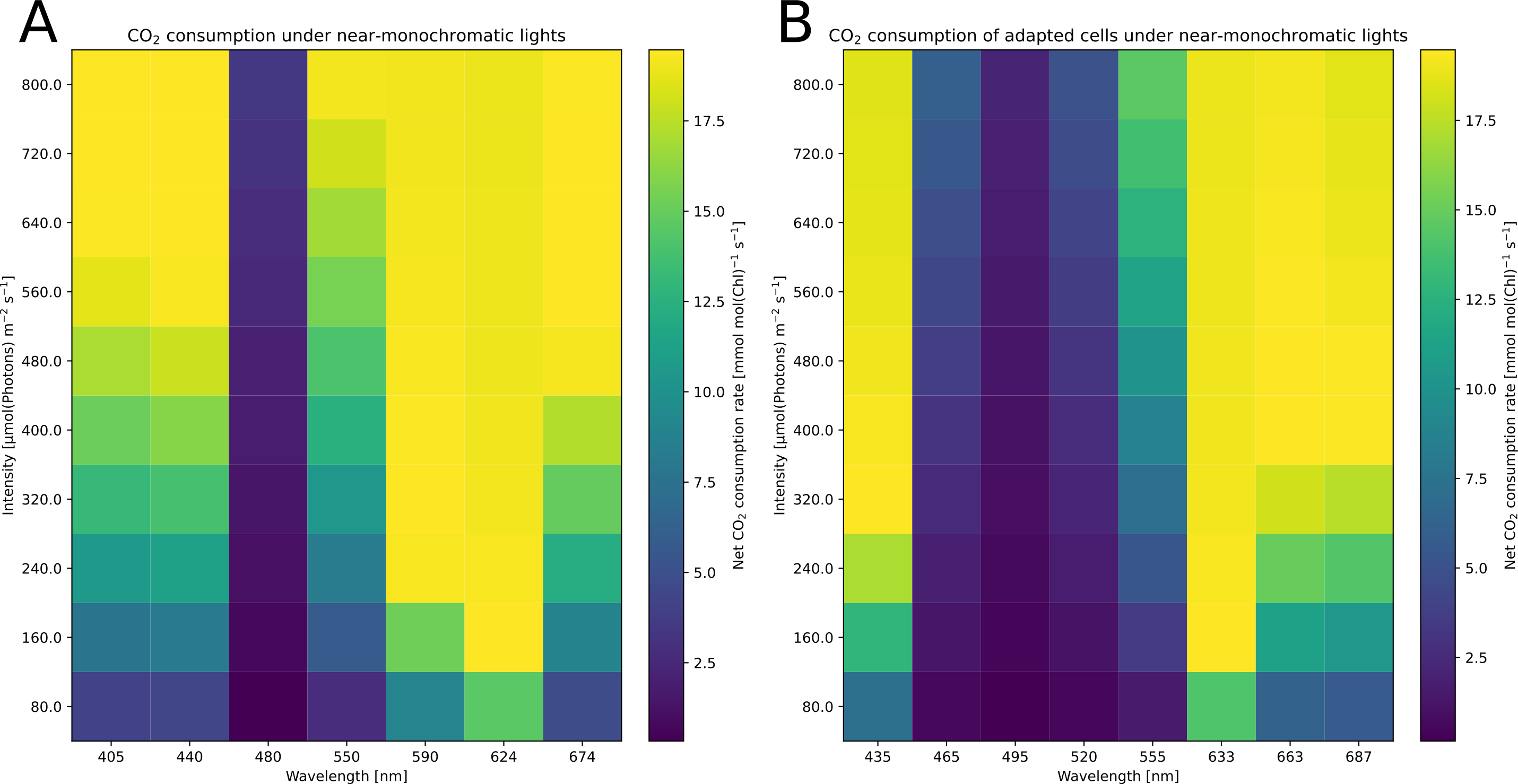

Supplement: S13 Fig — Simulations with default pigment composition (A) or with pigment compositions of Synechocystis sp. PCC 6803 grown under the respective light color (B). The adapted models were parameterized using pigment, photosystems, and PBS measurements. The models were then simulated to steady-state with the respective light condition, and the CO2 consumption is shown. The CO2 fixation rate is the lowest between 465 and 555 nm compared to all other tested conditions. Under 633 nm light, the highest CO2 fixation rate is reached at the lowest intensity. Compared to the unadapted simulations, the efficient usage of red light and inefficient usage of blue light is more pronounced. (TIF) [file pcbi.1012445.s014.tif]
