# Supplementary material for: Shedding light on blue-green photosynthesis: A wavelength-dependent mathematical model of photosynthesis in Synechocystis sp. PCC 6803
Source: PLoS Comput Biol. 2024 Sep 12;20(9):e1012445. doi: 10.1371/journal.pcbi.1012445 (PMC11421815; doi:10.1371/journal.pcbi.1012445)
Supplement: Attachment — Submitted filename: Resonse to the reviewers.pdf [file pcbi.1012445.s015.pdf]

Junior Professorship Computational Life Science | RWTH AACHEN UNIVERSITY

Tobias Pfennig
Doctoral Researcher
Computational Life Science | Department of Biology
RWTH Aachen University
Worringerweg 1, 52074 Aachen, Germany

11.03.2024

Dear Editor David Lea-Smith,

We would like to **resubmit** our manuscript, "Shedding light on blue-green photosynthesis: A wavelength-dependent mathematical model of photosynthesis in Synechocystis sp. PCC 6803." Our work provides a detailed, mechanistic description of the photosynthetic process in cyanobacteria and allows for systematic testing of existing hypotheses on not fully elucidated processes, such as state transitions. To the best of our knowledge, it is the **first kinetic model** of photosynthesis developed for cyanobacteria that can simulate its dynamics for various **light intensities and spectra**. It is developed based on well-understood principles from physics, chemistry, and physiology, and allows further testing of new assumptions about photosynthesis in cyanobacteria.

The first reviewer recognized the **relevance of such a kinetic model** but, as Reviewer 2, was not satisfied with its formal description. It has been requested that we improve the formalism, explicitly derive numerous equations, and make a clear distinction between model parametrization, validation, and predictions. In response to the reviewers' valid criticism of using different parameter sets for our analyses, we have defined a **new single parameter set** that all analyses are performed with, aside from experiments with light-adapted cells where we changed the pigment composition as input. Next, thanks to the reviewer's comments, we significantly improved the content of the manuscript by providing new Methods sections and expanding the SI with explicit derivations of our equations. On top of all rewritings, we have performed a new PAM-SP experiment to collect a new set of fluorescence data to **validate** our model (Fig. 2c). Finally, we have included a Jupyter Notebook with all additional simulations performed while responding to the reviewer's comments (sometimes explicitly requested, and sometimes as a consequence of in-depth further analysis). This file can be found together with the code on our Lab's GitHub repository:
https://github.com/Computational-Biology-Aachen/synechocystis-photosynthesis-2024

We disagree with Reviewer 2's statement that it is not a distinguished result itself to create a mathematical model that conforms reasonably to some available data nor that the results of our computational simulations shall be called ”results”. First of all, one of the fundamental points of creating mathematical models is to integrate evidence collected in a reductionist approach into one **comprehensive framework**, as a validation of the accuracy of our current understanding. Computational models allow researchers to simulate and study complex systems that are difficult to observe or manipulate in the real world. By capturing the interactions between different components of a system, models help scientists understand the underlying mechanisms driving the system behavior. We have accomplished that, by integrating data from numerous studies, using measurements collected under saturating and ambient CO_2 conditions, developing a generic model with validity for more than one condition, and avoiding overfitting to one particular dataset with advanced fitting routines. Secondly, prediction and forecasting are only one of many goals of computational modeling, next to enabling understanding of complex systems and hypothesis testing (in our case applied to state transitions). Finally, the biotechnological analysis of our model sets the ground for further exploration and indeed requires testing for validation. Nevertheless, it can systematically quantify the change in production potential for varying light conditions, and as it was pointed out to us after the discussions with the community present at CyanoWorld in Kassel in 2023, can therefore provide valuable insight into **redox imbalance limitations** during whole-cell biocatalysis.

Below, please find our detailed response to the Reviewers. We addressed all concerns and responded to all questions raised here, hoping this response will facilitate a faster decision regarding the acceptance of this work for publication in the PLOS Computational Biology.

On behalf of all authors
Tobias Pfennig & Anna Matuszyńska

Response to Reviewer 1

Reviewer #1: The model described and used in this manuscript is not perfect and it suffers from being too simple (my opinion). However, it is the **first kinetic ODE-based model** for cyanobacterial photosynthesis, as far I know. Thus, it should be published, but the manuscript needs to be improved to be accepted for publication, especially in description of the model.

Response: We would like to express our sincere gratitude for taking the time and effort to review our manuscript and for recognizing its worth in being shared with the scientific community. Your insightful feedback and constructive criticism have been invaluable in enhancing the quality and clarity of the model description. Some of the stated questions helped us realize ambiguity in our model descriptions, some led to repeated simulations.

General comment: It has been pointed out that we did not use the same set of parameters for all simulations, hence we repeated the simulations with the set used for the old Fig.3 With that set of parameters (summarized in Table S3) we observed an increase in the rate of cyclic electron flow under high light, hence we changed the description of NDH-1 mediated cyclic electron flow. Literature light response curves suggested that we overestimated the photosynthetic yield. Therefore, we have introduced a light conversion factor that describes the generation of excitations from 50% of absorbed photons. Predicted steady-state flux through CBB under various lights improved (Fig. 3b) as well as fluxes through various electron pathways (Fig. 1b). Accordingly, we have repeated our PAM-SP data fitting and used new parameter ranges for the alternative state transition models and provide now two PAM validation figures (Fig. 2a and Fig. S3, whereas for Fig. 2a we collected new experimental data and Fig. S3). Furthermore, we have repeated our analyses assuming saturating CO_2 conditions where appropriate and recalculated the assumed maximal CO_2 fixation rate. These results yield less conclusive results on the possible mechanism of state transitions than in our first submission, hence we significantly downplayed the worth of these findings. We have nevertheless decided to keep the section as a part of the main text, as one of the points of having a model is to create a framework for systematic testing of existing hypotheses and we find it noteworthy that considering the effect on PQ redox state, the currently favored mechanism of PSII quenching does not have a significantly greater effect on the oxidation of PQ (Fig. 4).

Your expertise and attention to detail have undoubtedly contributed to its improvement. Please find below the detailed answers to your comments and questions.

My detailed comments:

Introduction – The literature is not up-to-date. Following works should be cited:
Stirbet et al. (2019; Chlorophyll a fluorescence in cyanobacteria: relation to photosynthesis. In: Mishra A.K., Tiwari D.N., Rai A.N. (ed.): Cyanobacteria – From Basic Science to Applications. Pp. 79-130. Academic Press, London) – in places where cyanos and their ChlF is mentioned: Stirbet et al. (2020; Photosynthesis: basics, history and modelling, Annals of Botany 126, 511-537) – in places where modelling of photosynthesis and of ChlF is mentioned Lazar et al. (2022; Light quality, oxygenic photosynthesis and more,

Photosynthetica 60(SI), 23-56) – in places where light spectrum utilized by cyanos is mentioned

Response: Thank you for directing us to this literature. The works contain useful mechanistic explanations of cyanobacterial photosynthesis and a well-collected summary of mathematical plant models. They have been cited in appropriate places.

L150 – LET is not a circuit. A better wording should be used

Agreed. The wording has been changed to either “electron transport pathways” or “electron flows”.

Legend to Figure 1 – It is strange to consider Cyd, COX, and Flv as components of WWC. In your logic, also PSII and PSI should be parts of WWC. In fact, reactions starting by formation of superoxide from reduction of oxygen (the Mehler reaction, see review of Asada 2010, PCP) are the initial parts of the WWC. *Reactions of oxygen reduction, accompanied by usage of protons, to water, as occurring in Cyd, COX, and Flv are not parts of the WWC, as far I know*. The authors must check it.

Agreed. The term “Water-Water-cycle” is unfit to describe Flv, COX, and Cyd. This was an oversight. Instead, we decided to use the term “Alternate Electron Transport (AET)” as found in the literature (see e.g. https://doi.org/10.1016/j.bbabio.2015.10.007 or https://doi.org/10.1080/09168451.2014.882745).

There is no FQ in the figure, however, it is mentioned in the legend.

Agreed. The model we present does not include FQ and we mentioned it in the figure legend by mistake. We have removed it.

NDH-1 is described as NADPH dehydrogenase 1, however, it accepts electron from ferredoxin in the figure, thus, it cannot be NADPH-dehydrogenase but it is NADPH-dehydrogenase-like (NDH-like) complex, see related literature.

NDH-1 was given different names even in recent literature (https://doi.org/10.1016/j.plantsci.2020.110798, https://doi.org/10.1016/j.bbabio.2020.148354). We agree that “NAD(P)H dehydrogenase-like complex 1” best reflects the current knowledge body and we have made changes in the text and figure caption.

Figure 1 – I guess that in the case when Flv is not functional and flux from PSII is decreased due to lack of electron outflow to Flv, the decreased PSII flux is accompanied by increased NPQ or similar process. Is it right? If yes, it should be mentioned and shown.

This is correct. The decreased electron outflow in the simulated Flv mutant leads to a more reduced PQ pool and thus, an activation of internal PSII quenching. The quenching raises the heat dissipation of excitation energy as a form of NPQ. This effect is not visible under low light intensities, where Flv is normally not active, and under strong light saturation where all simulations show full reduction of the cells. We have added this analysis to the

manuscript plots (Fig. S7, p. 16), included an additional figure where the change in the redox state of electron carriers can be observed (Fig. S8, p. 16), and discussed the findings in the main text: l. 328.

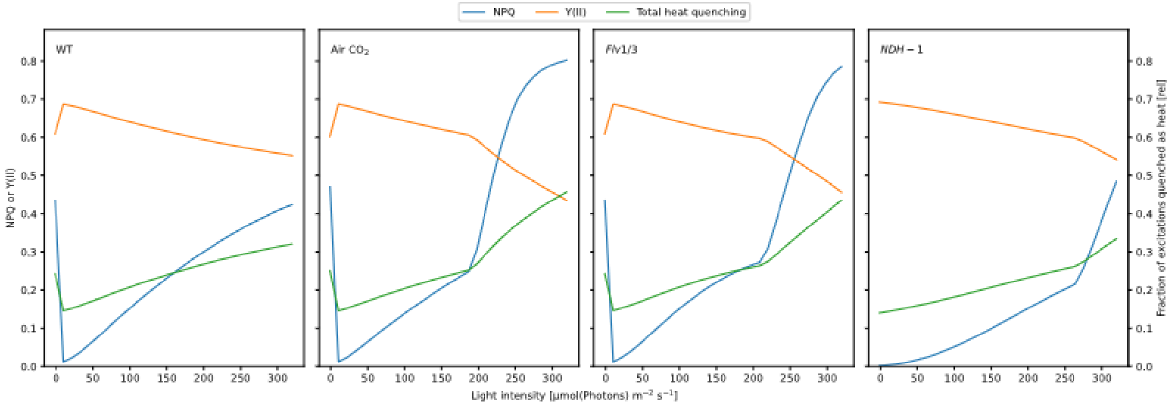

Fig. S5: **Calculated NPQ and photosynthetic yield (Y(II)) and the fraction of total excitations quenched as heat for four *in silico* lines** representing the wild type (WT) in saturating CO_2 and ambient air CO_2 level, a flavodiiron (*Flv*1/3) and NAD(P)H Dehydrogenase-like complex 1 (NDH-1) knockout mutant. The models were simulated to steady state for light intensities between 0.1 µmol(photons) $m^{-2}\,s^{-1}$ and 300 µmol(photons) $m^{-2}\,s^{-1}$. Modeled conditions as in Fig. 1.

L153 – Change “RuBisCO oxygenation” to “RuBisCO oxygenation function”

We agree that the wording is ambiguous. It has been changed to “Functions describing CBB and RuBisCO oxygenation…”.

L162 – Even if it is not important, you should mention why you set the maximal differences differently. What is the reason for this?

Agreed. When testing if a steady state was reached, we originally set a higher threshold for compounds with higher concentrations to have the test be similarly stringent on compounds differing in their concentration order of magnitude. We have now changed our approach to checking the Euclidean norm of *relative* concentration changes between the last two integration steps. Because the three regulatory processes, CBB redox regulation, state transitions, and OCP, have a much slower rate of change than the metabolic compounds, we set a more stringent threshold for them instead.

L168-171 – If by directionality you mean that the reaction is only forward or reversible reaction (as implied from the sentences) than, the reaction can be only forward or reversible no matter if the Gibbs free energy difference is positive or negative. Thus, the reasoning of this sentences seems to be wrong and further, nowhere in the text or in the supplement any calculation of the Gibbs free energy difference is shown. Thus, you should rephrase it. And if you uses this approach, you should mention that it was used in your previous papers, not in the modelling papers generally.

Agreed. The word “directionality” was wrong in this context. Indeed, when determining if a reaction would be implemented as reversible, we calculated Gibbs free energies under a range of reactant and pH values as an indicator. We have changed the sentence to “For each reaction, we calculated the Gibbs free energy ($\Delta rG'$, see supplemental information) [54,

55]. Only reactions with ΔrG′ close to 0 (> -5RT) under physiological conditions were described with reversible kinetics.” We have also added an explanation and example of the ΔrG′ calculation to the supplement**.**

L193-194 – How can reduced PQ pool molecules quench ChlF? As far I know, only oxidized PQ molecules were reported to quench ChlF. You probably mean that reduced PQ pool causes a switch of PSII to a state of PSII, which quenches energy by heat dissipation. It should be clarified and/or more described.

We have carefully revised the section in question and admit this was an uncareful shortcut in wording. We provided now explicit equations to elucidate the derivation process of all four possible mechanisms of state transitions (S1.6, p. 5-6). We believe these additions significantly enhance the clarity of our work. We appreciate your attention to detail and are confident that the revised version addresses the ambiguity raised in your comments.

Equation 2 – Exact definition of FPSI, FPSII, and FPBS must be provided. What model variables (states of the model) were considered to emit florescence and with what quantum yields or what are values of related rate constants (for fluorescence emission)?

Agreed. We have calculated fluorescence based on the photosystems' quasi-steady-state models and the fraction of free phycobilisomes. We have added a complete description of our fluorescence calculation to the main text p. 6-7 (subsection “Calculating the fluorescence signal”).

General comment to the model description in the supplement: the model is not described adequately well. For example, why there are symbols “-.” or “!-.” in table S3 instead of standard arrows. Further, the equations should be written by standard symbols and the reactants by their standard names, not by the symbols used in the model code – in this way you are kidding with the readers, since not all readers are familiar with such type of description. You must simply pay more time to a proper model description so that it can be unambiguously understood and reproduced even by a beginner in the field.

This comment is in line with several comments from the second reviewer and we have substantially rewritten the methods section, provided the model functions in mathematical description for clarity, and revised the parameter and reaction tables.

L222 and other places – The same curves as named by you as “PAM curves” can be measured by a non-PAM fluorometer. People usually mismatch the denotations and meaning. PAM fluorescence is a method for detection of any ChlF signal (with measuring flashes or/and with actinic light or/and with saturation pulse). But what is unique in your experimental data, is application of the saturation pulses, which enables to calculate related quantum yields, which you did not calculated, unfortunately. Thus, the correct name of the method is the saturation pulse method (see some review papers/chapters by Uli Schreiber, inventor of PAM).

Thank you for the comment regarding the terminology used for the fluorescence curves. We completely agree with your observation and admit we have been using the PAM term interchangeably with what we meant to be a saturation pulse method. We now refer to the method as the "saturation pulse method". We appreciate your clarification on this matter and

have ensured consistency with the terminology used in relevant literature, including works by Uli Schreiber. When discussing fluorescence in our work we almost exclusively refer to the measured fluorescence as the sum of PSII, PSI and PBS fluorescence. Therefore, we have refrained from using the term "ChlF".

L225 – Change "Our simulated steady-state …" to "Changes in our simulated steady-state …".

Due to the model changes we have made in response to the review (e.g. the implementation of a lower light to excitation conversion), the qualitative fit to data has been strongly improved. Therefore the sentence has been changed to "Changes in our simulated steady-state O_2 evolution rates are in quantitative agreement with the experimental data during low light and exceed measured rates under light saturation by ca. 20 % (Fig. S4)."

L229 – What is Bo and B1 in the legend of Fig. S2? It is related to my general comment on a poor model description.

Following our previous models that we have referred to in the main text, we describe the internal electron transport of PSII at a quasi-steady-state, as they are much faster than the model's other reactions. In this implementation, PSII has four states B_0 to B_3. We model PSI similarly in a three-state model (Y_0 - Y_3). We have added a short explanation in the main text (Activation of photosystems, p. 6) and a detailed description with schematic representation in the supplementary material (S1.3, p. 3-4).

L242 – Yes, "LET stagnates", but it would be better to write "CET stagnates"

We agree that the reduction in CET under high light is the more noticeable effect and could interest the reader. Because we have changed the implementation of NDH-1 in the revision process, the decrease in CET is less abrupt in the new simulations. Still, we discuss the observation in the main text. "For example, it can be seen that a reduced PQ poolunder high light leads to reduced CET mediated by NDH-1 and, in turn, a decreased CBB flux due to insufficient provision of ATP."

L255 – What "measuring protocol discrepancies" yare writing about? Authors of the referred paper are authors of this manuscript, so there should be no discrepancies.

We agree that our wording was too ambiguous. We meant to describe that not all saturation pulses reached could saturate and close the photosystems. This observation has been addressed in the experiment's publication. We have changed our wording to "By systematically comparing our simulation results and experimental data, we have revealed that the experimentally used saturation pulses were non-saturating in 480 nm actinic light and induced fluorescence quenching, as confirmed by follow-up experiments (see Fig. S14 in [55] and Fig. S6 in this work)."

L260 – Why do you use a new term "Dark Peak Fluorescence", when the Fm term defines exactly what it is?

We agree that common terminology should be used and therefore now use terminology as in Stirbet 2019: "dark-adapted minimal (F_0) and maximal fluorescence (F_m), the maximal

fluorescence in the light (F'_m), and the constantly measured steady-state fluorescence (F)".

L261 – What is QSS? It must be defined.

Agreed. We added the following explanation: In the photosystems model according to Ebenhöh *et al*. (2014): "we approximate the photosystems' internal electron transport at Quasi-Steady-State (QSS) as it is much faster than other described processes."

L263-264 – When you know the reason, have you checked the opposite scenario? Why not?

Due to the model changes, we made in response to the reviewer's questions and suggestions, our fluorescence fit under high blue light (OCP activation) has improved, see Fig. 3a. Unfortunately, our calculated F_m' are now consistently higher than the measured signal under other light colors, although the qualitative trend has been kept (e.g., increase of F_m' between State 1 and State 2, increase of F_m' in the NPQ relaxation).
We have tested alternative activation functions for the CBB and still observed activation of state transitions in the high blue light phase, so we rejected the hypothesis that the slow CBB activation might be responsible for lowering fluorescence signals. Instead, the results of our simulation indicated that the high intensities (photon flux of 1800 µmol m^{-2} s^{-1}) of 480 nm light are sufficient to partially light saturate the cells, leading to PQ reduction and the activation of state transitions. Measurements by Luimstra (2018) suggest that, under sufficient intensity, also blue light can become saturating.
There are different explanations for the remaining difference between our simulated and measured fluorescence. Importantly, our simulated cellular redox state should be validated further. However, a comparison with data is hindered by the, to our knowledge, limited availability of redox measurements. Further, the redox state of the simulated cell is dependent on the light input and all internal redox reactions. Thus, the reason for deviance under a specific condition might not be obvious. This is one of the current limitations of our model and will require the integration of new processes into the model.

L274 – What do you mean by "We slightly perturbed …" How much is slightly? What was maximal relative change of the model parameter (rate constant) to calculate the control coefficients? The are some rules in MCA to do it and you should mention it.

This is important information. We have improved the section on Metabolic Control Analysis and specified the perturbation (±1%).

L277 – "usually"? I would say "always".

Agreed. We deleted the word "usually".

L283 – Is reference to Fig. 3c correct? Should not be here also reference to Fig. 3b?

Agreed. We have corrected the wrong figure reference (note that we switched figures 3b and 3c) "Accordingly, the maximal simulated CO_2 consumption is reached at lower light intensities for these spectra (Fig. 3b)."

L294 – Change "more reduced PQ pool" to "more reduced PQ pool in state 2"

We agree that the sentence is unclear about the PQ-pool relation to state transitions. We changed the sentence to “Therein, a higher fraction of PSII excitations is lost as heat in state 2” and added “We model the transition to state 2 under a reduced PQ pool while oxidized PQ promotes state 1.”

L296-297 – This description of PBS-mobile model is wrong. When there is set state 1 (state 2), PBS is attached to PSII (PSI).
And
L297 – There should be “PBS detach from the photosystems II” instead of “PBS detach from the photosystems”.
Further, according to your statements and schemes in Fig. 4A, you assumed attachment of PBS to PSI in states 2 in the PSII quenching model, spillover model, and in PBD-detachment model, which was not assumed in the referenced review (30).
Since these are significant, mistakes/differences from original referenced suggestions, the authors must check, if these mistakes are only in the text and the simulations were done correctly or not. If simulations are wrong, they must be corrected, including related conclusions.

We assume a constant attachment of PBS to PSI in all state transition models but the PBS mobile model. This value has been experimentally obtained, and the review by Calzadilla and Kirilovsky (2020) also indicates that PBS are not exclusively attached to PSII (“Mutations in the PBS terminal emitters affect the energy transfer to one or both photosystems in different cyanobacterial strains.”). Therefore, we chose not to alter this attachment in models where this is not necessary. We also checked the implementation of the state transition models and confirmed that they comply with the state transition models in the review.
However, we agree that L296-297 described the PBS-mobile model wrongly and have corrected it to “... the PBS-mobile model, where PBS attach preferentially to PSI or PSII in states 1 and 2, respectively”. To avoid ambiguity, we have added the definition of all state transition models to the SI (S1.6, p. 5-6).
Lastly, we have repeated the analysis of state transition models using our revised model. Unfortunately, the results are inconclusive, and we have rewritten our discussion accordingly. When simulating the PBS-detachment model with detachment from only PSII, most parameter-varied models show a more oxidized PQ redox state, likely due to a lower attachment of PBS to PSII in general and an increase of F_m’ under state 2. Therefore, this

alteration of the PBS-detachment model doesn't show improved behavior.

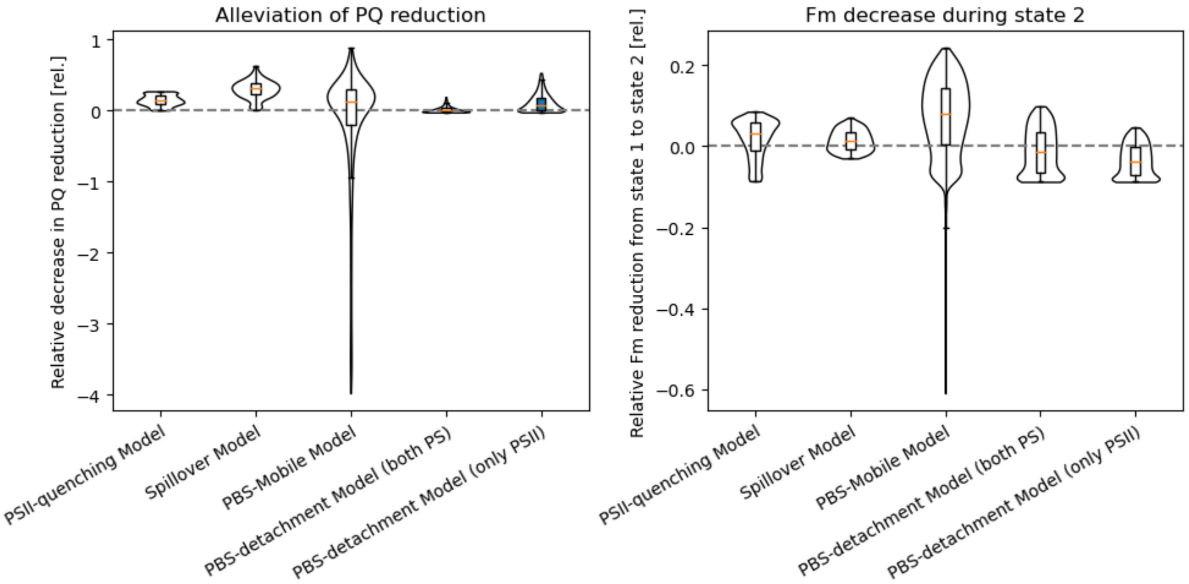

L300 – Why do you write in “Dark Steady-State Fluorescence” high initial letters?

We have corrected this spelling.

L302 – Why do you write in “Peak Fluorescence” high initial letters?

We have corrected this spelling.

Sections started at L269 and L313 explore effects of different excitation wavelengths on model outputs but in fact, nowhere is mentioned, how (mathematic formulas) different spectral excitations affect the system function. This must be clearly described in the text or in the supplement. In connection to that, for example, the legend to Table S2 writes about “light attenuation” but it is nowhere described how it is treated mathematically, which, however, must be done.

Agreed. As this is an important part of the model we have added a manuscript section to explain how we model light absorption by pigments and the estimation of photosystem excitation rates (p. 5+6). Additionally, when simulating experimental data with a known culture density, we try to estimate how light absorption by the culture will attenuate the light. We estimate light attenuation using the Lambert-Beer law as described in the SI (S2.3, p. 13). Using the law, we calculated the mean light intensity and spectrum in the culture as the effective light for simulation.

Table S1 – Is the “standard electrode potential” really the correct term? What about midpoint redox potential? And further, the redox potentials are always determined for redox couple, for example, for Qa/Qa- couple the midpoint redox potential is -140 mV. Thus, it must be corrected. Further, in section “kinetic constants” the constants are termed as rates, which is wrong, since they are rate constants not rates. Rate has the same meaning is flux through a reaction.

Yes, to calculate Gibbs free energies we use standard electrode potentials, which can be derived from midpoint potentials. We have added a section on Gibbs calculations in the SI (S1.4, p. 4+5). We collected all standard electrode potentials from Lewis *et al.* (2022), who also provide the mentioned Qa/Qa- potential of -140 mV. We do agree that the term “rate” for parameters is incorrect and have renamed the affected descriptions to “rate constant of…”.

L374-375 – A role of Ribisco is tressed on the basis of caclulation of the control coefficient of Rubico, which might be misleading, since sedoheptolose bisphosphatase has been found to have the highest control for CO_2 assimilation rate (papers by Poolman in 2000 and 2001).

Our specific mention of RuBisCO as the controlling factor was an oversight in the discussion. Indeed, our model’s lumped CBB reaction cannot distinguish between the control of different included reactions. In our revised model we have changed the control analysis to only compare photosystems and, explicitly, the whole CBB as the sink for energy equivalents. As such, control of the CBB could very well be due to sedoheptulose bisphosphatase.

A last question. How does the model consider the high PSI/PSII ratio known for cyanos when PSI as such is not considered in the model?

We do include the PC to Fd electron transport by PSI in the model. We estimate the light absorption by PSI and simulate the internal states under a quasi-steady-state assumption, similar to PSII. We take the high PSI:PSII ratio into account by 1) having a high PSI-trimer concentration (PSItot), which corresponds to a monomer ratio of ca 2:1 by default, and 2) by assuming that chlorophyll excitations for light absorbed by chlorophyll a are transferred to the photosystems according to the photosystems’ ratio and their content of chlorophyll. We have improved our description of light absorption by providing a new section on photosystem activation (p. 6) and a full description of photosystems in the SI, including the schematic representation of processes considered within PSI (S1.3, p. 3+4).

Response to Reviewer 2

Reviewer #2: Comments on "Shedding light on blue-green photosynthesis: A wavelength-dependent mathematical model of photosynthesis in Synechocystis sp. PCC 6803" by Tobias Pfennig et al. submitted to PLoS Computational Biology.

The authors present an ODE-based model of photosynthesis of the cyanobacterium Synechocystis sp. PCC 6803, primarily focussed on the electron transport chain but also including (simplified) adjacent reactions (ATP usage, carbon fixation). As a (nonlinear) ODE-based model the model requires knowledge of a substantial amount of kinetic parameters. The model follows traditional methods of kinetic modelling, in particular parameters are primarily based on literature values.
Models such as the one presented here are suitable to advance understanding of the cyanobacterial electron transport chain, including its dynamic properties. The latter puts kinetic models apart from stoichiometric constraint-based models that typically require far fewer parameters, but are also far more limited in their scope.

While I recognize the importance models such as the one presented here, I think the present model (and its description) can be substantially improved. In particular, I think the two main shortcomings of the manuscript are (1) the results should clearly demonstrate novelty and biological significance. Currently, the main achievements are that the model conforms reasonably to some available data (which in itself is not a distinguished result) and the results beyond that are inconclusive (state transitions) or very speculative (biotechnology). Secondly (2), model construction, the underlying assumptions, and in particular parameter estimation are poorly described.

Response: We want to extend our appreciation for your meticulous review of the manuscript and our model's (incomplete) description. Your attention to detail and insistence on precision in our descriptions have been immensely beneficial in refining the Method section and Supplementary Information. Your critical comments have prompted us to reconsider our language choices and to provide a more accurate and detailed description of our model construction in both the Method section of the main text, as well as in the Supplement. Some of the questions here forced us to perform additional analysis on the model which led to three changes to the model:

- We repeated all simulations once again with the set of parameters fitted to PAM-SP obtained for strain grown under 430 nm (Fig. 2a)
- Because our model previously simulated a sudden decrease of cyclic electron flow under high light which was not consistent with literature growth curves we changed the description of NDH-1 mediated cyclic electron flow.
- As literature light response curves suggested that we overestimated the photosynthetic yield, we have introduced a light conversion factor that describes the generation of excitations from 50% of absorbed photons.

Accordingly, we have repeated our PAM-SP data fitting and used new parameter ranges for the alternative state transition models. After these changes our analysis of the possible state transitions model is less conclusive than before, hence we only mention the possibility of testing such processes with the model but do not make the strong claim about providing

evidence for some of the mechanisms. Before answering each question in detail we would like to highlight that our model development and refinement did follow the traditional steps of initial formalism, data integration, and parameter estimation based on the collected data, prediction, and validation. We have not been careful enough to distinguish between simulation results and predictions, for which we first of all apologize. Your efforts are deeply appreciated, and we are confident that the model description has been substantially improved. A section on parametrization, with clear reference to the literature used for parameter estimation or fitting, has been added to the Methods section (Table S3). On top of all rewriting, we have performed a new PAM-SP experiment to collect a new set of fluorescence data to compare our model against to (Fig. 2c).

Finally, we would like to address your claim, that is not a distinguished result itself to create a mathematical model that conforms reasonably to some available data. One of the fundamental points of creating mathematical models is to integrate evidence collected in a reductionist approach into one comprehensive framework, as a validation of the accuracy of our current understanding. Computational models allow researchers to simulate and study complex systems that are difficult to observe or manipulate in the real world. By capturing the interactions between different components of a system, models help scientists understand the underlying mechanisms driving system behavior. We have accomplished that by integrating data from numerous studies, using measurements collected under saturating and ambient CO_2 conditions, developing a generic model with validity for more than one condition, and avoiding overfitting to one particular dataset with advanced fitting routines. Secondly, prediction and forecasting are only one of many goals of computational modeling, next to enabling the understanding of complex systems (MCA, theoretical production capacity, impact of light) and hypothesis testing (in our case applied to state transitions).

Estimating kinetic parameters is a major challenge for kinetic models, and the authors will surely be familiar with Von Neumann's elephant: "With four parameters I can fit an elephant, and with five I can make him wiggle his trunk". Here, the trunk certainly wiggles, but we do not know how the parameters were determined. More specifically: the text makes several strong claims for the achievements of the model "Our model accurately predicts the partitioning of electrons through four main pathways, O_2 evolution, and the rate of carbon fixation. Additionally, it successfully captures chlorophyll fluorescence..." (in abstract). As far as I can see, these strong claims are not substantiated by data.

We agree that we overused the word “prediction” for most of the simulation results, without distinguishing between results obtained during parametrisation vs validation vs actual predictions. This would indeed mislead or confuse readers. We have carefully corrected that. However, we disagree with the claim that the model does not capture (chlorophyll fluorescence) dynamics properly. We have captured the trend in both steady-state fluorescence and F_m levels, switching between various light regimes. For the first iteration of such a model (we are not aware of any other existing dynamic models of photosynthesis developed for cyanobacteria that consider both light intensity and spectrum) we can reproduce a number of phenomena such as the activation and relaxation of acclimation processes

Moreover, some of the claims are contradictory (and likely incorrect, see below for further examples). For example, in the caption of Figure 1: "Our model correctly predicts the

proportion oflinear electron flow (blue) to four other pathways to be around 60% [39] in wild type (WT)". Later, it is stated that (line 231): "We parameterised the flux through the CET to yield approximately 35 % of the total PSI electron flux [39]." Was the model parameterized according to the data from ref [39] or is it a prediction? According to which criteria/data was the statement "Our model accurately predicts the partitioning of electrons through four main pathways, ..." evaluated?

Thank you for bringing that to our attention. Upon reviewing it, we see that you're absolutely right. The word “predict” should not be used. Instead, we have changed it to “reproduced”. We have used the data from [39] to parameterize two free parameters, the light conversion factor “lcf” and the parameter “vNQ_max”, which is the maximal rate of PQ reduction by NDH-1.

In line 253 we are informed that "... parameters controlling the fluorescence composition, state transitions, NPQ, and CET were *updated* to get the best agreement between our simulated trace and the original data 255 (Fig. 2a)". (emphasis by me).
What does "updated" mean exactly? According to which methods (adjusting values manually, using a fitting algorithm)?
Is the model overfitted? (i.e. many potential updated parameters would give an equally good fit)?
If yes, how were the final parameters chosen? These examples are not exhaustive, but rather illustrate the kind of problems that exist with respect to the handling of parameters. I consider such issues to be of high importance when evaluating a kinetic model that claims predictive value.

Absolutely right and we pledge to share our work according to FAIR principles. We have rewritten a substantial part of the method sections to provide a clear description of how we redefined our model, which is well visible in the manuscript with tracked changes. We are very much interested in reading your feedback after this round of review, as we aimed to clarify all ambiguities.

Additionally we would like to justify here our choice for manual parameterization. In the context of biochemical kinetic modeling such as this one, the justification for manual parameterization over sophisticated algorithms lies in the nuanced understanding and expert knowledge of the system under investigation. While fitting algorithms may offer automation and potentially expedite the parameterization process, they often lack the interpretability and domain-specific insight that manual parameterization provides. By manually tuning model parameters, we could leverage their understanding of biochemical pathways, enzyme kinetics, and regulatory mechanisms to ensure that the model accurately reflects the underlying biology. This approach allowed us to incorporate experimental data, empirical observations, and prior knowledge into the parameterization process, resulting in models that are not only robust (we did not aim at overfitting to a particular experiment) but also biologically meaningful. Additionally, manual parameterization offers flexibility in addressing model complexities and adapting to unique experimental conditions or system variations that may not be adequately captured by automated algorithms.

We also want to highlight that we are continuously improving our methods and aimed at streamlining the parametrization process. After initially fitting the parameters to the PAM-SP

data manually, we attempted an automatic fitting using least-squares methods. However, the particular data provided a challenge because of the complexity of the fluorescence data. Fitting the thousands of measuring points in a PAM-SP dataset is unrealistic and selecting single points for fitting (such as F_0, F_m', or F_v/F_m) can introduce a bias in the fit. Furthermore complex simulations require significant amounts of time, which hinders the usage of fitting algorithms that perform large numbers of simulations. We have considered deriving and fitting to fluorescence parameters like NPQ but some important characteristics visible only in the steady state fluorescence signal would not be considered then. Because of this, we decided to remain with the manual fitting with a focus on qualitative reproduction of the data.

It is further noted that the remaining section (Fig 3 onwards) are largely computational/speculative and not based on any further measurements or available data.

We do believe that computational results can be treated as valuable results. Indeed, the results of MCA and time courses simulated for monochromatic light are results of our theoretical analysis and require further testing, but they set the ground for such testing and narrow down the conditions that our experimental partner may decide to test.

My overall suggestion is that the "Methods" must have a dedicated section that describes the parameterization of the model. Vague statements, such as "We derived kinetic parameters from literature measurements, e.g. in vitro kinetics, fluorescence, or gas exchange rates" (line 139) are *not* sufficient.

Absolutely. It was not our intention to hide any details of our work and we have made sure that with this revision we are more careful with wording and providing clear descriptions in the text. We have added the requested section of model parametrization (p. 4+5).

In general, model description should be expanded. It is a fairly straightforward model (this, in itself, is not a bad thing), almost all processes involved have been described in far more detail (in particular core pETC, here PSII and PSI are described by one mass-action equation, respectively).

This is a misunderstanding, stemming from our initial poor description of the methods. Our model also includes more sophisticated descriptions of both photosystems: Quasi-steady-state systems describing the internal states of PSII (four-state model) and PSI (three-state model) to reflect steps of both light absorption and charge separation. Otherwise, we would not be able to simulate the saturating pulse protocols to calculate fluorescence signals. We have i) improved the scheme of the model to include processes inside of photosystems (Fig. 1a, p. 4), ii) added a section on light activation of photosystems to the Methods section (p. 6), and iii) expanded the description in the supplement, additionally providing schematic representation of processes (S1.3 p.3+4).

Also some descriptions sound more elaborate that the model actually is (L154, "we also considered a simple Carbon Concentrating Mechanism (CCM) implementation by ...", not described incorrectly in the main text, but the "simple CCM" is really just multiplying the concentration by a factor 1000. Why 1000 exactly? The cited Ref is far less specific).

We agree that our model does not include an elaborate kinetic representation of the CCM. The model was designed to investigate electron flow dynamics considering the important physiological features of cyanobacteria. The CCM was one such feature as it strongly influences the carboxylation efficiency of RuBisCO and, thus, the rate of carbon fixation as the major electron sink. However, describing the exact mechanism of carbon concentration was not necessary to consider its effects. Thus we decided to use the simplest implementation yielding reasonable results. We have compared our model simulations to experimental measurements of oxygen production under variation in carbon availability (Fig. S2). While our maximal simulated O_2 production rate lays far below those of Benshop *et al.* (2013) (but above those measured by Schuurmans (2014)), we could see that an increase of the intracellular partial pressure by factor 1000 could reproduce the dynamics of O_2 evolution for ambient CO_2 concentrations and above. Therefore, we believe that our implementation, while simple, is reasonable for its intended purpose. We recognize, however, that CCM using NDH-1 subforms would be a reasonable addition to the model, as it directly involves electron transport, and we discuss this possibility in the manuscript.

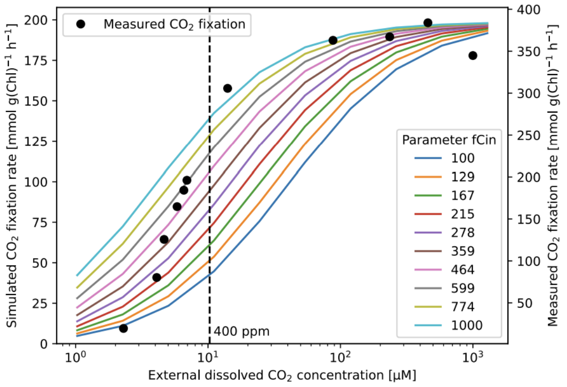

Fig. S2: **O_2 production under light intensity variation *in vivo* and *in vitro*.** The parameter $fCin$, representing the intracellular to extracellular partial pressure ratio as increased by the carbon concentrating mechanism, was varied in the simulation between 100 and 1000. Benschop *et al.* [120] measured oxygen evolution with 800 µmol(photons) $m^{-2} s^{-1}$ light of *Synechocystis* sp. PCC 6803 grown under 20 ppm CO_2 and varied the dissolved CO_2 in the medium (C_i). The simulation used a cool white LED. Our simulated O_2 evolution rates are ca. half of the measured rates. However, the rate dynamics in ambient air (400 ppm) or above are well reproduced with $fCin = 1000$. The model overestimates oxygen evolution at very low C_i concentrations.

The Method section would generally benefit from a clearer description of the model, e.g. how many reactions, how many parameters, etc ...? Even when looking at the Supplemental Text it is difficult to extract this information (not helped by miniscule font in Table S1 and terms like "visually fitted", whatever that may mean).

We have added such a summary sentence to the Methods section. Additionally, we have improved the parameter table (Tab S3) to be more readable and understandable, clearly stating which parameters were fitted to which data. See below a comprehensive summary of the model fitting process:

The model consists of 17 ODEs, describing 27 reactions or processes. It depends on 95 parameters of which 37 were taken directly from literature (including 5 pigment absorption spectra), 9 rate parameters were estimated from direct or related literature rate measurements by determining an approximate rate through the pathway and dividing this rate by the assumed intracellular substrate concentrations, 6 parameters were physical quantities, 6 parameters were set according to the experimental irradiance, measuring light, O_2 & CO_2 condition, concentration of cells, and temperature, 8 parameters were estimated from experimental data (PBS_PS2, PBS_PS1, PBS_free, PSItot, PSIItot, and pigment

concentrations), 2 inhibition constants were estimated assuming they are equal to the Michaelis constants, the intracellular salinity S was assumed to be equal to seawater, intracellular buffering capacities of lumen and cytoplasm (bHo and bHi) were assumed constant (Ebenhöh, 2014), and 25 were fitted to different datasets or literature during model refinement:

- kUnquench, kQuench, KMUnquench, kOCPactivation, kOCPdeactivation, OCPmax, and PBS in fluo_influence were fitted to PAM-SP data (Zavrel, 2023) (Fig. 2a),
- parameter vNQ_max was fit to 30% CET measured in cyanobacteria (Theune, 2021) (Fig 1b),
- parameter lcf was fit to 15 electrons PSI^{-1} s^{-1} of LET under 300 µmol m^{-2} s^{-1} light (Theune, 2021) (Fig 1b)
- parameter kCBBactivation was fit to activate the CBB within ca. one minute (Nikkanen, 2020),
- parameter KMFdred was fit to have a low CBB activity in the dark but high activity at irradiances where respiration is inhibited (at irradiance above ca. 10 µmols m-2 s-1) (Schuurmans, 2015),
- parameters KHillFdred and nHillFdred were fit to induce fast Flv activation at ca. 50 µmols m^{-2} s^{-1} white irradiance (Schuurmans, 2015),
- parameter fCin was fit to CO2 assimilation data (Benschop, 2003),
- parameters k_respiration and k_aa were fit to ca. 20% PQ reduction in dark (Khorobrykh, 2020),
- parameter k_Q was fit to a level allowing for PQ oxidation under blue irradiance (Kirilovsky, 2014),
- parameter KMPGA was fit to prohibit Photorespiration under very low concentration of CBB intermediates (represented by 3PGA),
- parameters KMNQ_Qox and KMNQ_Fdred were fit to produce continuous CET flow under high PQ reduction near light saturation (Theune, 2021),
- parameter kATPsynth was fit to produce sufficient ATP for supplying the CBB,
- parameter k_F1 was fit to provide a low fluorescence yield to PSI,
- parameter kNADHconsumption was fit to provide reasonable NADH/NAD+ ratios in darkness and light, and
- kATPconsumption and k_pass were fit to provide low capacity flows consuming ATP and dissipating proton gradients.

The final model has been validated against the newly obtained data (added to Fig. 2c, p. 9).

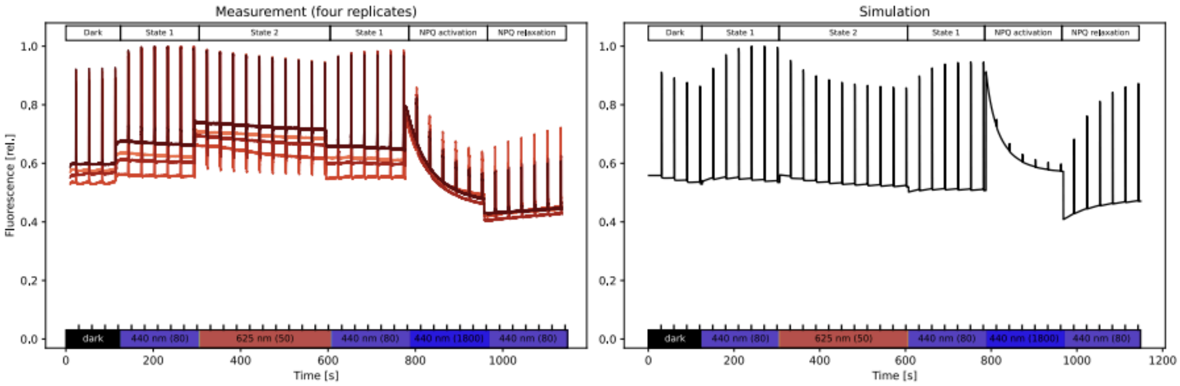

(c) Model validation comparing PAM-SP fluorescence traces *in vivo* and *in silico*. Four experimental replicates are shown. Simulations assume 1 % CO_2 supplementation and use the default parameters (see a)) and pigment set. The model reproduces the fluorescence dynamics during most of the experiment except for overestimating steady-state fluorescence during the strong blue light phase. The lower bar depicts the light wavelength and intensity (in parentheses, in µmol(photons) m^{-2} s^{-1}) (lights used: 440 nm at 80 µmol(photons) m^{-2} s^{-1} and 1800 µmol(photons) m^{-2} s^{-1} and 625 nm at 50 µmol(photons) m^{-2} s^{-1}, saturating pulse: 600 ms cool white LED at 15 000 µmol(photons) m^{-2} s^{-1}). The measurements were performed with a Multi-Color PAM (Walz, Effeltrich, Germany). Cultures of *Synechocystis* sp. PCC 6803 were grown under bubbling with 1 % CO_2 and 25 µmol(photons) m^{-2} s^{-1} of 615 nm light for ca. 24 h. For the measurement, 1.5 mL culture was transferred to a quartz cuvette and dark-acclimated for 15 min prior to each measurement.

Furthermore: whenever there are statements that claim "the model accurately predicts", or "model can accurately capture" (Cap. Fig S1) or "The model was validated against published measurements ...", it must be made clear how the correctness of the prediction was established, whether the data has been used in model parameterization. And if yes, to what extent the model is overfitted. (and what does "accurately capture" mean? The curve is either fitted, i.e., parameters were adjusted to match the curves, or it is a prediction, and the parameters were sourced elsewhere. I do not know what "capture" refers to in this context).

Thank you for this comment. We agree that we have failed to accurately report our modeling process and findings in our previous submission. We believe that the careful corrections we made now will allow future readers to clearly distinguish between parameterization, validation and predictions. We do not provide quantitative measures to assess the quality of fits as PAM curves were fitted manually. This is an often used procedure in developing kinetic models for biological processes where even technical replicates vary consistently between each other (see e.g., the PAM curves of our validation experiment, Fig 2c) and we are foremost interested in capturing the qualitative trend (e.g., decrease of steady fluorescence levels, increase of NPQ during the switch between light phases). Finally, our aim was to create a general model of photosynthesis in cyanobacteria and using sophisticated fitting algorithms possessed a higher risk of overfitting it to a particular data set (found in the literature or produced by us).

L16: "iit is crucial to investigate their intricate interplay with the environment in a particular light". (intensity? quality?)

We agree that this phrasing is ambiguous We have changed the sentence to: "... interplay with the environment including the light intensity and spectrum."

L19: Spelling: "an ordinary differential equation-based model for cyanobacterium Synechocystis sp. PCC 6803"

We have corrected the spelling to: “... an ordinary differential equation-based model for the cyanobacterium…”.

L20: "Our model accurately predicts the partitioning ...". Substantiate claim!

As explained previously, we tried in this revised manuscript of the model to make more modest claims. The phase in question has been changed to “Our model can reproduce a variety of physiologically measured quantities, e.g. experimentally reported partitioning of electrons through four main pathways…”

L21: "Additionally, it successfully captures chlorophyll fluorescence signals ...": what does it mean that a model "successfully captures" something? How is "success" defined in this context?

We agree that the wording is ambiguous. The fitted model shows the expected qualitative changes to the level of F_m’ and often F under state transitions and high-blue light adaption by OCP. Because “success” is, indeed, a vague term, the phasing has been changed to “Our model qualitatively reproduces fluorescence emitted under various light regimes, replicating Pulse-amplitude modulation (PAM) fluorometry experiments with saturating pulses.”

L29: Keywords: Avoid keywords that are already in the title

Thank you for this advice. We have exchanged “mathematical model” and “photosynthesis” with “biocatalysis” and “electron transport”.

L49 and following: A lot of space is devoted to explaining that prokaryotes lack organelles that are found in plant cells. On the other hand, methods like PAM are used without almost any explanation. It is clear that (most) of the authors come from the plant field, but the introduction should be aimed at a broad readership. (The section here reads a bit like an undergraduate term paper: "cyanobacteria can perform photosynthesis and respiration, allowing for auto-, hetero-, and mixotrophic lifestyles [15]. But, due to the lack of mitochondria, cyanobacteria ...". Also everything is very plant-centric).

This is true that this section was written in a plant-centric way. Our motivation was that there are a number of excellent dynamic models of photosynthesis developed for plants, and for readers of the PLOS Comp Biol we wanted to highlight the differences between the organisms to justify the development of a new dynamic model, specifically for the cyanobacteria. We have significantly shortened the introduction on organelles and added an explanation of the importance of PAM (in particular PAM-SP) measurements. A paragraph on the importance of light quality (color/spectrum) has been shifted to the end of the Introduction to further highlight the importance of its inclusion into the model.

L136: "A comprehensive list of all the model parameters utilised in this study, ..., is provided in Table S1 and Table S2". This section is insufficient. Model description should be in the main text. How many variables, how many parameters, etc …

We have included a quantitative description of the model in the manuscript's main text (p.3) and have significantly expanded the Method section that now contains additional subsections on: code implementation, model parametrization, reaction kinetics,

implementation of monochromatic and polychromatic light sources, activation of photosystems, calculating the fluorescence signal, mechanisms of state transitions, MCA, and the analysis of the production capacity.

L143: Providing a github is very good, but the main text and Supplement should also be sufficient to fully understand the model. For example, a list of reaction rates in the Supplement would be helpful (currently Table S3 in the pdf seems to be garbled and is very difficult to read)

Fully agree. We have substantially improved both the Methods section of the main text, (see our responses above) as well as the Supplement. You will now find a more detailed explanation of our simplified kinetics and of all reaction kinetics that we modelled using different descriptions in the supplement. Additionally, we have reworked the tables of parameters and reactions following your comments and hope that you will find them significantly more transparent now.

L165: Why was mass action kinetic linked to (known) regulatory properties? Whether MA is a good/poor approximation is not only determined by whether the reaction is regulated. How were rate equations motivated in general, given that many elements in the pETC have been modelled in great (biophysical) detail. Statements like "because parsimony" are too vague.

When creating this model we intended to create a parsimonious model that describes all processes necessary to simulate electron transport in a general cyanobacterium. We also aimed at using preferentially easy-to-understand, low-complexity functions for the kinetic descriptions. This was to allow for a better understandability by scientists trying to employ our model. As one of the first kinetic models developed specifically for cyanobacteria, we also intend our model to be used as a scaffold where more complex descriptions of processes can be plugged into (as done in previous models for plants). Therefore we decided to describe reactions using MA kinetics (modified for simplification) unless a different description was necessary to reproduce desired behaviour. Even then, we tried to use established kinetics such as Michaelis Menten or Hill, to keep a high level of transparency. Due to our pour model description before it might have indicated that this model was not based on well-understood principles from physics, chemistry, and physiology, which we hope with the updated version will become clear.

L171, Eq (1): what happened to the stoichiometric coefficients here?

In our simplified MA kinetics, the order of reaction does not depend on the stoichiometric coefficients corresponding to each species in the balanced reaction. This step was motivated by the inclusion of the proton exchange, which has to be considered in the mechanistic model of photosynthesis to account for the creation of the proton motive force that drives the ATP synthase. As variations of the intracellular pH translate to 10-fold concentration changes, protons therefore have a massive influence on MA kinetics, especially for stoichiometries larger than one. Generally, in order to determine the reaction order, the power-law form of the rate equation is used. But in some reactions (e.g., cytb6f) four protons are pumped through the thylakoid membrane. Eq. 1 describes the simplified MA kinetics that we employ in the model. With the simplified MA kinetics we tried to circumvent inflating the control of protons in the system.

L175: I do not understand how that the equilibrium equality can be violated by anything described here. Please explain.

Indeed the statement in our first submission was wrong, thank you for pointing it out. Instead, we wanted to clarify that the rate constants of forward and reverse reactions are not equal due to our simplification. By simplifying the MA kinetics and omitting the stoichiometric coefficients, we make two approximations:

$k \cdot \prod c_{S_i}^{n_i} \approx k^+ \cdot \prod c_{S_i}^{n_i} / \prod c_{S_i}^{n_i - 1} \quad \text{and} \quad k \cdot \prod c_{P_j}^{m_j} \approx k^- \cdot \prod c_{P_j}^{m_j} / \prod c_{P_j}^{m_j - 1}$. For any stoichiometric coefficient larger than one this leads to $k^+ \neq k^-$, which is the correct statement. We have added the kinetics derivation and the explanation of the inequality of rate constants to the supplemental information (S1.2, p. 2+3)

L178 "This implementation enabled us to simulate various light-adapted cells by updating the parameters corresponding to measured pigment composition and ...". The description is too vague. Also the Supplement is not very helpful. Implementing different spectra is one of the main "selling points" of the model, the description should be far more specific.

We were happy to include detailed descriptions in the main text under the sections Implementation of monochromatic and polychromatic light sources (p. 5+6) and Calculating pigment association (S1.5, p. 5).

L219ff, Eq (3): I am not convinced that summing of absolute values is a good idea (it is not commonly used, for good reasons). Processes with more elements will get higher values (you primarily count elements/subprocesses). Why did you not use the median or mean?

We agree that this is a better approach and calculate the mean of absolute values instead. We also decided to streamline our results and display now only mean control over the CBB flux by photosystems that correspond to the source of energy and the CBB cycle as the main sink (See Fig. 3c).

L225: "Our simulated steady-state O_2 rates are in qualitative agreement with the experimental data (Fig. S1).". I do not find Fig. S1 very convincing. Do either the data or the simulations have error bars (e.g. the latter due to parameter uncertainty). I see a light dependent increase in O_2 evolution, as expected, but almost any model would predict that, and the simulations are not very close to the data.

With the final refinement of the model, we have improved the fit, especially in the lower light intensities range, as seen in the figure below (Fig S4). The original experimental data (copied below) has error bars, but as seen (i) aside from light intensity 100 µmol m^{-2} s^{-1} they are small (yes, we know, it is a relative term), (ii) as we did not receive the original raw data and extracted the values from the graphics, we did not find a way to include them. Our results do not include error bars as the results were run with one set of final parameters that we ended up with after months of model refinement and calibration to yield qualitative agreement with a number of available experimental data. While photosynthesis-irradiance curves are the general output of a lot of photosynthesis models, please keep in mind that the calculated O_2 in the model is a result of integrating a system of differential equations with complex light absorption function and detailed electron flow description, not a statistical

model created to fit this particular result. We believe that it is still important to show that our model can reproduce fundamental measures of photosynthesis such as a PI curve.

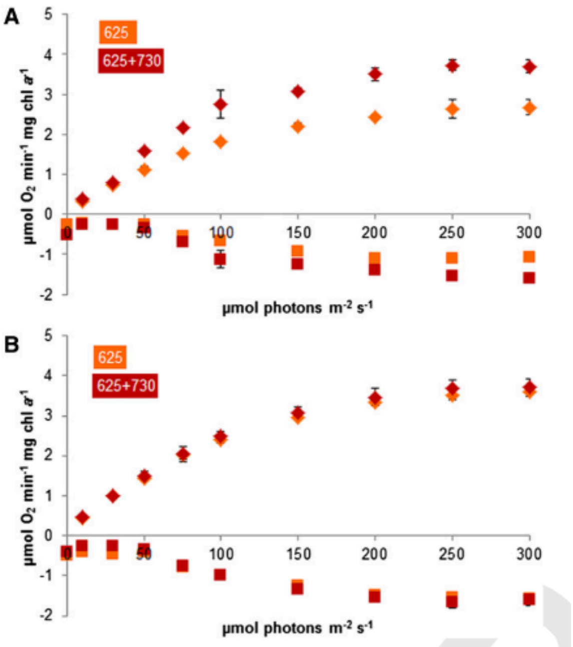

Figure 6. Oxygen evolution rates of *Synechocystis* sp. strain PCC 6803 cultures in increasing intensities of 625-nm light, recorded with MIMS. $^{18}O_2$ was added at the start of the experiment to monitor oxygen consumption in the light. Depicted are net photosynthesis (which is the sum of the measured oxygen production and the consumption [diamonds]) and respiration (squares). A, BG-11 with 0.5 mM $NaHCO_3$. B, BG-11 with 50 mM $NaHCO_3$. Orange symbols, 625-nm light only; red symbols, 625-nm light + 25 μmol photons m^{-2} s^{-1} 730-nm light. Data shown are averages of biological duplicates with SD. [See online article for color version of this figure.]

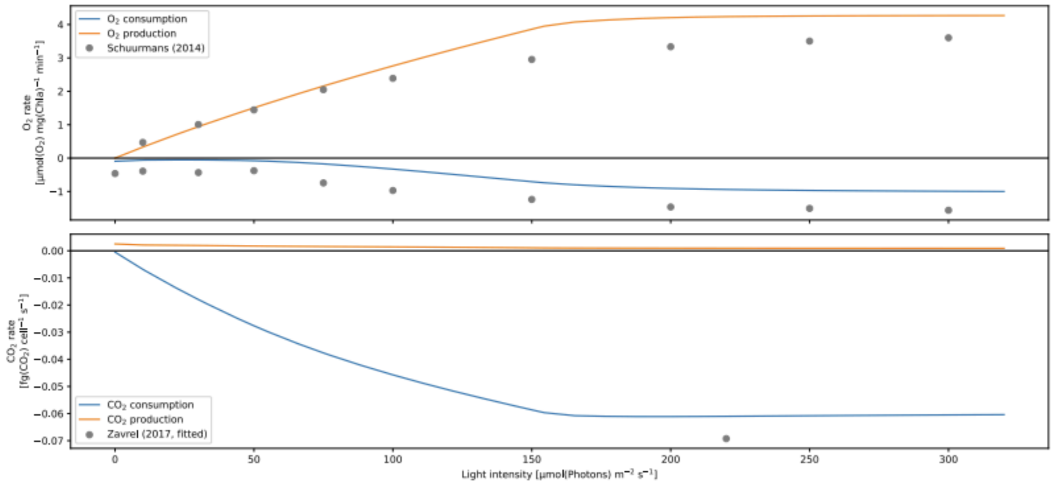

Fig. S4: **Rates of change in O_2 and CO_2.** Our model reproduces oxygen production and consumption (respiration + three terminal oxidases) rates for increasing light intensities, as compared to measured rates digitized from graphs by Schuurmans *et al.* (2014) [64]. Data points are from measurements of cells with 625 nm illumination with 50 mmol L^{-1} $NaHCO_3$. Small variations in the data and the lack of error bars are due to the digitization process. The simulated carbon fixation rates are displayed with the measurement used for parameterization [133]. Simulations were run using a 625 nm light (gaussian LED, $\sigma = 10$ nm). We calculated light attenuation in the culture using Equation S67 with default pigment concentrations and a constant 2 mg L^{-1} sample chlorophyll concentration.

L323: "We parameterised the flux through the CET to yield approximately 35 % of the total PSI electron flux [39].". How was this done, which parameters? ("Flux" is not a parameter). Fig 2 Caption: "The simulations were adapted to the given strain by changing the parameters corresponding to pigment composition". How were simulations "**adapted**"? Were parameters changed? Which parameters? By which method/criteria/algorithm?

This is a recurring criticism of our work and we have responded already above to some extent. Again, to parameterize the model and yield the appropriate CET flux, we fitted the maximal rate of NDH-1 mediated CET ("vNQ_max"). In nearly every case where we used the word "adapted" we meant "fitted" parameters. We carefully corrected the wording to not cause any ambiguity. We fit this parameter and the introduced light conversion factor ("lcf") manually, so our model can simulate flux of the linear electron flow of approximately 15 electrons PSI^{-1} s^{-1} and, moreover, that flux should correspond to ~60 % of overall electron flux under saturating CO_2 conditions, as reported by Theune *et al.* (2021).

L253: "and parameters controlling the fluorescence composition, state transitions,254 NPQ, and CET were updated to get the best agreement between our simulated trace and the original data255 (Fig. 2a).". There needs to be significantly more detail what terms like "updated" actually mean, and which information was used to parameterize the model and what is a "prediction".

Please see our previous explanations on how we improved the description of parametrization, the general description of the model, and the differentiation between parametrization, validation, and prediction. We hope our improvements reach your desired level of detail.

L258 "Our simulation predicted especially well the transition to state 1 in blue light and the NPQ action and relaxation". Was that after the parameters were "updated"?

Indeed, this observation was after we fitted parameters to the PAM-SP data. Therefore, "predicted" was the wrong word to use, as we wanted to point out that the model can *reproduce* the expected behavior if fitted. We hope you will find no such ambiguity in our reworked description of parametrization.

Fig3: Labeling is a bit of a mess. There are capital letters for subplots, but also lower case letters. More importantly, Fig 3c (?) shows CO_2 consumption as a function of light intensity. Net CO_2 consumption should correspond to growth rate (in the absence of excretion of products), but **the curve does not look like any growth rate ever reported.** I note that the rate is measured relative to g(Chl), but g(Chl) is assumed to be constant in the simulations. How do the authors reconcile the statement that the model "correctly (!) predicts the rate of carbon fixation" with the fact that no measured rate of carbon fixation I am aware of looks even qualitatively like this curve.

We have investigated the reason for the criticized shape of our simulated CO_2 light response curves and find that it is due to the high reduction of the PQ pool under saturating light. Our previous implementation of NDH-1 mediated cyclic electron flow used simplified MA kinetics and, therefore, the rate linearly decreased with with availability of oxidized PQ. In response, CO_2 fixation decreased under high light due to the lowered ATP synthesis. As pointed out, we did not find literature evidence for our simulated decrease in CO_2 fixation in response to

high light. Therefore, we decided to model NDH-1 using Michaelis Menten kinetics with a low K_m for oxidized PQ in our revised model. We chose MM kinetics as the simplest implementation that could explain why NDH-1 flux is kept up under the decreasing levels of oxidised PQ in high light. Miller *et al.* (2021) found a saturation of NDH-1-mediated proton flow under low light intensities, matching the behavior of a MM kinetic. While this implementation does not prevent the eventual limitation of NDH-1 by PQ, it retains NDH-1 flux for much higher light intensities. Simulations using this new function show a gradual decrease of CO_2 fixation under high light and the shape compares well to e.g. O_2 evolution rates measured by Luimstra *et al.* (2018). We deem this implementation reasonable for the purpose of our model, however, for research focused on NDH-1, a more complex implementation could be attempted. For example, Zhang et al. (2020) found, that NDH-1 flux is actively upregulated under high light in addition to transcriptional upregulation.
We believe that our revised model simulations of O_2 evolution are in agreement with literature measurements such as Luimstra *et al.* (2018), who also measured O_2 evolution in unadapted strains (i.e. at constant chlorophyll content). We agree that our model cannot predict changes in pigment concentrations (or other model parameters) due to cellular adaption and discuss this fact in our manuscript.

L331ff: The entire section is very speculative, with little grounding in data.

The light quality effect on the cyanobacterial production of compounds is far from being fully explored. We are aware of several works investigating growth and compound production under light variation, e.g. saxiotoxin and neosaxiotoxin (Carneiro, 2009), cryptophycin-1 (Polyzois, 2020), isoprene, glycogen, and lipids (Rodrigues, 2023). Generally, these studies found the cyanobacterial growth to be highest under red or white light and diminished under blue. Compound production on the other hand was often increased under red light but showed considerably more variation, with some compounds being induced by blue illumination. Especially the production of pigments like phycobilins (Mishra, 2012; Hotos, 2022), carotenoids (Pagels, 2021), or compounds derived from pigment synthesis pathways like 2-methylisoborneol (Su, 2023) showed strong induction under certain light colors due to chromatic acclimation. As such, light color is highly important for optimizing production. But finding optimal experimental conditions may, for example, be hindered by the active regulation of pigment synthesis - processes that could be overcome by cellular engineering. We hope that you agree that a large advantage of modeling is that it can simulate the theoretical optima of biological systems and guide experiments towards them. We agree that the results of our MCA or simulated compound production have not been validated yet. However, we think that simulated results can still provide a direction to science and hope that this work will spark more interest in performing such experiments.

L335: "In this work, we present our mathematical ODE-based photosynthesis model as a quantitative in silico tool with high predictive power and customisability." The "high predictive power" has to be substantiated. Currently it is not.

We believe this statement will change after reading the updated version of the manuscript where we explain better the calibration process and provide a special validation figure. Nevertheless we incorporated the recurring feedback that we are making too strong claims and have used more humble descriptions.

L340 "our model can simulate illumination within the full visible spectrum (400 nm to 700 nm), also accounting for the pigment composition of the strain". But pigment composition and g(Chl) per cell will change under different spectra and intensities. Can the model predict that? If not, how can the model make predictions about, e.g. optimal conditions for biotechnology? If the pigment composition has to be measured before any simulation, the predictive values is limited.

To the best of our knowledge, these pigment adaptations are not occurring within the time frame of simulated experiments performed with our model (e.g. SP-PAM simulations). Secondly, pigment composition is a parameter in our model. Hence, we see a possibility of creating a new pigment adaptation model as an input to this model to simulate the change in pigment composition in response to time and light. We did not attempt to create such a function in this work, but we are currently establishing a new collaboration for performing pigment analysis and are interested in further work in this direction.

L347: "The model was validated against published measurements of gas exchange rates and electron pathway348 fluxes (Fig. S1). The good estimation of oxygen production rates supports ..." Do we look at the same Fig S1? I do not find the figure very convincing. How was the "validation" performed exactly?

Please see our previous answer regarding Fig. S1.

L351: "Our simulations show the dominance of LET (ca. 60 %; [39]), ..." Compare with: "We parameterised the flux through the CET to yield approximately 35 % of the total PSI electron flux [39]." The constant obfuscation about what is a prediction and what was fitted is a MAJOR problem of this manuscript.

Again, we are sorry for the confusion our poor and incorrect description caused. We hope that with the updated manuscript it will become clear that our fault was in writing and not model construction and with all necessary corrections this model will be accepted to be shared with the readers of PLOS Computational Biology. The model has been parametrized to yield approximately 15 electrons PSI^{-1} s^{-1} linear electron flow (blue) for a fraction of 60 % under saturating CO_2 conditions, as measured in wild type (WT) (Theune *et al,* 2021)

L361: "Our simulations support a PSII quenching model [62] rather than PBS detachment and spill-over, as suggested previously [30]". The simulations are inconclusive, but the PSII quenching is also widely accepted. And [30] is broad discussion and does not argue that the PSII quenching model is wrong.

Our result became less conclusive after the refinement and we have significantly downplayed the significance of this analysis. We have nevertheless decided to keep the section as a part of the main text, as one of the points of having a model is to create a framework for systematic testing of existing hypotheses. We agree that the PSII quenching is widely accepted as the most probable mechanism and this is also the reason why we kept that mechanism as the default one for all other analyses. We found it interesting to show how the model can quantify the possible contribution of each mechanism to alleviating the redox state of PQ, an important effect of state transitions, but we do not observe conclusive differences between all four mechanisms.

L400: "Our model considers the CBB as the main sink for energy equivalents which might not be the case for cells with higher growth rates, where the production of other biomass components can become more significant (e.g. glycogen [84])." How do you assume "production of other biomass component" happens without the CBB (or with a reduced CBB)?

It was not our intention to imply that other biomass components are produced without CBB action. These downstream pathways could become significant sinks of energy equivalents under the given circumstances and would then warrant inclusion in the model, thus changing the overall consumption of ATP, NADPH, and others. We have clarified this by changing the sentence to "However, reactions downstream of the CBB, such as glycogen production [84]), could pose additional significant sinks depending on the cell's metabolic state." (p. 13)

L433: Several authors are not listed in the "Authors contributions".

All authors participated in vital and extensive discussions, which we initially summarised as "all authors discussed, read, and approved the manuscript." Following this comment, we have now adapted the Author Contribution list based on the journal guidelines and CRediT Taxonomy (https://journals.plos.org/ploscompbiol/s/authorship#loc-author-contributions).

Overall summary: Any claims of predictive power have to be substantiated. The part on biotechnology is too preliminary to be considered a "result".

Thank you for your feedback. We have addressed your concern by clarifying our parametrization process and providing additional validation curves to substantiate any claims of predictive power. We have also simulated isoprene production (as a sink with the correct ATP, NADPH, and Fd consumption; see the new analysis of biotechnological production) using pigment concentrations measured in Rodrigues *et al.* (2023). Our estimated production seems to mostly follow the experimentally measured growth rate with the exception of an underestimation of the 550 nm, low light condition. Indeed, Rodrigues *et al.* discuss their observation that isoprene production is not directly coupled to cellular growth. Comparing the stoichiometric model simulations performed in that work, we can see that they propose a similar order of preferential lighting for isoprene production - except for a stronger penalization of blue light. Therefore, we believe that the cellular isoprene production is affected by processes not captured by either model, such as the physiological impact of isoprene on the cell, reducing the yield.

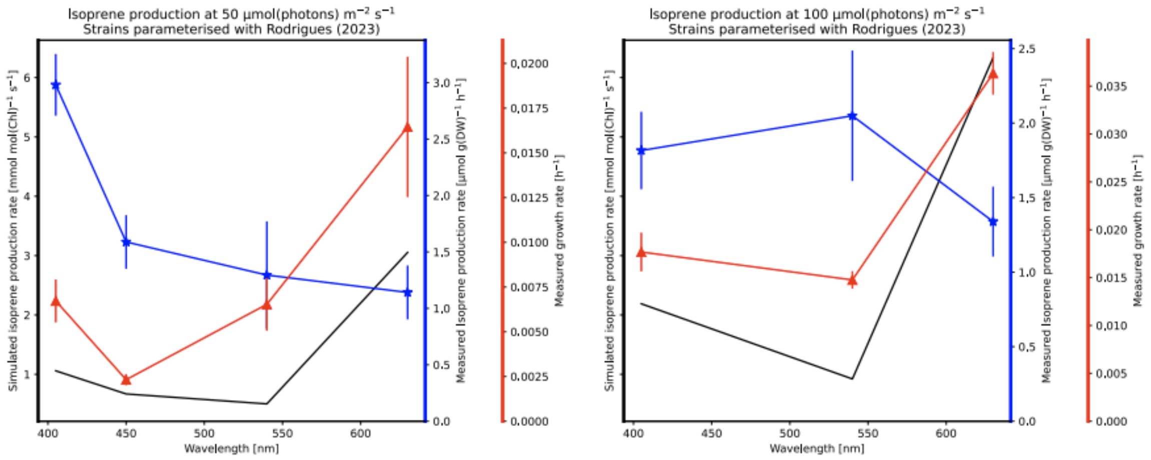

Fig. S13: **Comparison of measured isoprene production and simulated production capacity under light variation**. We show the growth and isoprene production rates measured under monochromatic illuminations (405 nm, 450 nm, 540 nm and 630 nm at 50 or 100 µmol(photons) $m^{-2}\,s^{-1}$) by Rodrigues *et al.* [29]. The model was adapted to the measured pigment composition (see Table S4) and simulated to steady-state under the respective monochromatic lights. We added a sink reaction to the model, consuming energy carriers in the ratio corresponding to isoprene (19 ATP, 11 NADPH, 4 Fd_{red}), including the cost of carbon fixation. We disabled the CBB reaction and limited ATP and NADPH concentrations to 95 % of their total pools. Simulations used a Gaussian LED (σ =10 nm).

As discussed before, we believe that also simulation results are valid results as they give a theoretical direction that can be further explored.
