# Supplementary material for: Shedding light on blue-green photosynthesis: A wavelength-dependent mathematical model of photosynthesis in Synechocystis sp. PCC 6803
Source: PLoS Comput Biol. 2024 Sep 12;20(9):e1012445. doi: 10.1371/journal.pcbi.1012445 (PMC11421815; doi:10.1371/journal.pcbi.1012445)
Supplement: Attachment — Submitted filename: Response_to_reviewers.pdf [file pcbi.1012445.s016.pdf]

Junior Professorship Computational Life Science | RWTH AACHEN UNIVERSITY

Tobias Pfennig
Doctoral Researcher
Computational Life Science | Department of Biology
RWTH Aachen University
Worringerweg 1, 52074 Aachen, Germany

12.06.2024

Dear Editor David Lea-Smith,

We would like to **resubmit** a revised version of our manuscript, “Shedding light on blue-green photosynthesis: A wavelength-dependent mathematical model of photosynthesis in Synechocystis sp. PCC 6803.”

We are delighted to see that the changes made to the first version satisfied **all comments** of Reviewer 1. Regarding nine remaining comments of Reviewer 2, we have now performed a comprehensive analysis utilising Monte Carlo methods to evaluate the robustness of our selected parameters. This extensive analysis has significantly enhanced our confidence in the model.

During the revision step, we have gained a deeper understanding of the underlying dynamics and parameter sensitivities within our model. The **Monte Carlo simulations** allowed us to systematically explore the parameter space, ensuring that our model's predictions are reliable across a wide range of possible parameter values. This additional layer of validation, prompted by the reviewer's feedback, has greatly strengthened the overall quality of our work. We acknowledge that there is a plethora of kinetic models available in the field; however, many of these models do not provide such detailed robustness analysis. We are pleased that the reviewer encouraged us to undertake this rigorous validation step, as it has provided us with even greater confidence in the robustness and applicability of our model to the study of photosynthesis.

Additionally, in response to the reviewer's concerns regarding the previous contribution to the field of photosynthesis, we conducted further analysis using a model **ensemble approach** for the state transitions mechanism. A model ensemble involves generating and analysing multiple models, testing various possible structures, to capture the range of possible behaviours of the system. Previously, we employed this approach at a single light intensity to analyse the mechanism of the state transition. In response to Reviewer #2’s advice to provide more conclusive results, we now extended our analysis to scan a wide light intensity range and systematically test the effect and importance of state transitions under various light regimes. Our results highlight how these transitions significantly impact the efficiency and regulation of the photosynthetic process. With increasing light intensity, the hypothesised mechanism involving phycobilisome (PBS) detachment failed to provide alleviation of redox stress. The other mechanisms showed alleviation under all tested lights. While higher light intensities seemed to reduce the effectiveness of the state transitions, the PBS-mobile model (some PBSs move from one photosystem to the other changing the antenna size of both photosystems)

retains the highest effect. We further found that the transition to state 2 could lower carbon fixation as a tradeoff for lower redox stress.

In summary, we are grateful for the reviewer's suggestions, which have led to substantial improvements in our manuscript. The incorporation of Monte Carlo methods and model ensemble analysis has provided a more **robust** and **comprehensive** validation of our model. We believe that these enhancements significantly strengthen our contribution to the field of photosynthesis research and we hope that our work will be now accepted for a publication in the PLOS Computational Biology.

On behalf of all authors
Tobias Pfennig & Anna Matuszyńska

Response to Reviewer 1

Reviewer #1: The authors answered all my questions/comments and revised the manuscript accordingly.

Response: We are delighted to read that the changes made to the manuscript were well accepted. Thank you for your time and efforts in reviewing our work.

Response to Reviewer 2

Reviewer #2: Review: Shedding light on blue-green photosynthesis: A wavelength-dependent mathematical model of photosynthesis in *Synechocystis* sp. PCC 6803 by Pfennig et al.

The authors have made extensive changes to manuscript, as required. The manuscript has clearly improved. The current version would have been a suitable first submission (as the actual first submission was defective on several levels).

I have evaluated the manuscript carefully and have a number of comments, also taking into account the response letter of the authors.

Response: Thank you for acknowledging the improvement of this manuscript. Please find our detailed answer to your comments below. We would like to thank you for the time you have devoted to this work and for thought provoking comments. We surely enjoyed the conversation (and the dictatorship joke). During the revision step, we have gained a deeper understanding of the underlying dynamics and parameter sensitivities within our model. We performed Monte Carlo simulations to systematically test the robustness of parameters initially selected. This additional layer of validation, prompted by your direct feedback, has greatly strengthened the overall quality of our work. We acknowledge that there is a plethora of kinetic models available in the field; however, many of these models do not provide such robustness/sensitivity analysis. We are pleased that your comments encouraged us to undertake this rigorous validation step, as it has provided us with even greater confidence in the robustness and applicability of our model to the study of photosynthesis.

Comment #1: I am glad to see that claims about predictive power have been removed. "Upon reviewing it, we see that you're absolutely right. The word “predict” should not be used" and "... we incorporated the recurring feedback that we are making too strong claims and have used more humble descriptions".

I want to emphasize that my criticism was not about a lack of "humbleness" in the descriptions, but about (a lack of) scientific truthfulness/correctness.

One example: I noted the inclusion of a (very primitive) carbon concentration mechanism by multiplying the value of CO_2 by 1000. The value was cited as derived from the literature. Now I learn that you actually tested different values and "we could see that an increase of the intracellular partial pressure by factor 1000 could reproduce the dynamics of O_2 evolution ...".

I do not recall that this was noted anywhere in the original manuscript. Instead you claimed to quantitatively predict O_2 evolution. Such claims are not acceptable. I note that the issue is (almost) resolved in the current version, but I still hope that the senior authors discuss with the junior researchers how to present scientific results and how to properly back up claims about "predictive power".

This is no minor issue. Readers must be able to trust researchers not to make misleading claims about predictive power of models, in particular in interdisciplinary work (the first submission failed in this respect).

Response: This information has been added into the manuscript precisely as a response to your concerns in the first round of the revision. We had extensive discussions regarding testing, validating and predicting and we want to reassure you that all additional analysis that you have read now in the revised version are the direct result of these conversations. We agree that this is not a minor issue and we are confident that these claims were fully resolved.

Comment #2: The authors write: "We disagree with Reviewer 2's statement that it is not a distinguished result itself to create a mathematical model that conforms reasonably to some available data nor ...."

My sentence was actually part of a wider quote (that I deleted to shorten the review), taken from James Bailey [Mathematical Modeling and Analysis in Biochemical Engineering.... Biotechnol Progress, 14: 8-20 (1998). https://doi.org/10.1021/bp9701269]: "There is a zoo of mathematical models in the biochemical engineering and mathematical biology literature. Many of these appear, particularly to the naive reader (and sometimes to the sophisticated one), to have little purpose other than calculating numbers which conform reasonably to experimental data. This is, in itself, not a distinguished endeavor; it is not particularly difficult, and it teaches little." The quote closely ties to von Neumann: "With four parameters I can fit an elephant, and with five I can make him wiggle his trunk". Your model is indeed an example of this phenomenon: the model goes through a series of ad hoc adjustments that make it conform to data and suit your particular data. We already saw the factor 1000 above (fitted to give the best possible result).

Further: "As literature light response curves suggested that we overestimated the photosynthetic yield, we have introduced a light conversion factor that describes the generation of excitations from 50% of absorbed photons" ... which is yet another ad hoc factor.

Further: "Because our model previously simulated a sudden decrease of cyclic electron flow under high light which was not consistent with literature growth curves we changed the description of NDH-1 mediated cyclic electron flow".

And so on ...

While you can do all that, you should also be aware that such adjustments to fit the present data have severe implications for the general validity and for the the predictive power of any model. What makes you certain that the "light conversion factor" remains the same across conditions? And if it is not constant, what does this mean for the model to be used to explore different conditions (as explicitely claimed as a goal in the manuscript), short of re-parameterizing for each condition (such a requirement would again severely limit the utility of the model)? I note that you also already envision further adjustments if needed, i.e., from the response letter: "Therefore we decided to describe reactions using MA kinetics (modified for simplification) unless a different description was necessary to reproduce desired behaviour."

Response: Great article! Thank you for sharing. Especially with the following sentence of the mentioned citation "... *I believe that one reason mathematical biology receives so little respect from biological scientists, and is generally not recognized as a credible research tool in biological science and in biotechnology discovery, is failure to communicate clearly and persuasively the reason for making the model*" we understand now that many of your questions were motivators for us to communicate better what does the model bring new. We hope that you will see that with the additional ensemble modelling of state transitions we could shine more light on the plausibility of some hypothesised mechanisms. Now, on the site of ad hoc adjustments: again, changes made to the model were far from random and we adhered to the standards by separating testing and validation data, moreover many of the changes were motivated by experimental evidence. We agree that a model should not be

subject to arbitrary changes. However, as evidenced in the updated, corrected version, we followed the scientific method while developing the model. We performed stepwise refinements, in which we targeted various phenomenological features of the cyanobacteria. E.g, you pointed out previously that our simulated net CO2 consumption should correspond to growth rate, but our curve "does not look like any growth rate ever reported". In response we have investigated the reason for the criticised shape of our simulated CO2 light response curves and find that it is due to the high reduction of the PQ pool under saturating light. Any further changes into the model structure or parameterization were hence motivated with experimental evidence and needed to capture the phenomenon. Performed changes did not negatively affect the results of simulating previously well captured phenomena (we were not individually cherry picking parameter sets but developing a model that can reproduce all phenomena with one parameter set). We have now additionally performed 10 000 simulations with perturbed parameter sets to check the sensitivity and we calculate residues to provide quantitative measures for the accuracy of the fits. In this context we checked that our manually fitted model was close to (for +/-10%) and on (for two-fold) the Paretto front. We additionally performed simple optimisation to minimise the residues of our manually fitted parameters and quantify the difference between the new, optimised set and previous, manually fitted.

Comment #3: The quote above continues to highlight roles of models beyond fitting, as you also point out ("Secondly, prediction and forecasting are only one of many goals of computational modeling, next to enabling understanding of complex systems and hypothesis testing ..").

I fully agree with that. Most models (and some of the best ones) are not about predictions but are, for example, about explaining/understanding phenomena. However, it was your choice in the initial submission to claim predictive power. I would encourage the authors to think carefully about what can be learned from the model in its current stage. That is there a profound new biological insights the model brings, other than being a "platform" or that models are useful in general? Is there any genuine biological insight that can be drawn from the model in its present state? What do I learn about the biology of cyanobacteria that I was not able to learn without the model? This is a serious question and all modellers should ask this question during writing. In the present case, I note that many of the potential genuine (and specific) results are rather speculative or inconclusive (state transitions, biotechnology).

Response: We thank the reviewer for reconsidering the presentation of the biological insight that the model can provide. We have revisited the results of the model for possible state transitions, as in the initial submission, we considered insight into their mechanism as one of the most interesting results. We have now repeated the initial analyses for various light intensities, testing the impact of state transitions under moderate and high light. We used the model ensemble approach for all four models of state transitions perturbing their parameters 1000 - 10000 times. We observed that our modelled state transitions had generally the highest effects under low light. The PBS-mobile model had the highest dynamic range of PQ redox states under all light intensities and would therefore be a beneficial mechanism to cyanobacteria. As suggested in our original submission, the detachment of PBS could not produce the expected oxidation of PQ under medium to high light and is therefore unlikely. While theoretical, these results still provide novel insights and support novel hypotheses. We also found that the simulated alleviation of PQ redox state was accompanied by a lowered carbon fixation. Therefore, state transitions could be a target for biotechnological modulation to maximise productivity.

Secondly, we found that the control over the CBB flux by FNR and Cytochrome b6f differed between light intensities or monochromatic light colours. We saw that a simulated overexpression of Cyt B6f was most effective under low intensity orange light or high blue

light. On the other hand, the model simulated an up to 5% decrease in carbon fixation under low blue light, Generally, FNR overexpression was most beneficial under high light. We therefore provide novel targets for biotechnological optimisation of cyanobacteria.

Overall, there are several points on the biology of cyanobacteria that can be learned from the model. First, it provides understanding of the dependency of individual electron fluxes on the light intensity (Fig. 1b). Second, it provides insight into the mechanism of state transitions (Fig. 4) that still remains an open question. With the push toward more statistical analysis we provide more evidence for exclusion of one previously assumed mechanism of operation under assumption that its main benefit is to maintain the redox state of the cell (p. 14, Fig. 4). Third, by probing fluxes and synthesis rates of metabolites or products of interest under various light sources (Fig. 5b, Fig. S8-S12), a complex understanding of the wavelength dependency and limitations of cyanobacteria metabolism can be obtained. Light quality is one of the factors that drive global cyanobacteria distribution (Holtrop et al., 2021) and it has a substantial effect on cyanobacteria physiology. However, despite the recent advances in physiological studies on the light quality acclimation in cyanobacteria (Bernát et al., 2021; Zavřel et al., 2024), wavelength dependency of many pathways remains unknown. In this sense, this model has become very useful, since it allows to predict values that would be difficult to measure experimentally, such as pH of lumen or cytoplasm, fraction of Fd-red or PQ-red (Fig. S8). In addition, the model allows testing the effect of individual wavelengths (or a combination of wavelengths, simulating various light sources) on various aspects of cellular energetics (Fig. S10-S12).

The model allows us to perform virtual experiments, hence our choice of wording for "computational platform". The power of this feature is demonstrated nicely in Fig. S9 where the comparison of CO2 consumption in Synechocystis cultures fully acclimated to monochromatic lights or with a fixed pigment composition is tested. The results of this simulation allow us to gain novel insight into the effect of short-term exposure to monochromatic light, being relevant for both natural populations and controlled cultivations. Last but not least, since the model can be adjusted to other organisms than Synechocystis, it can be expected to provide insight into light quality acclimation in other types of chromatic acclimators in the future. Synechocystis is type 2 chromatic acclimator, currently there are seven types of chromatic acclimation known (Sanfilippo et al., 2019).

Bernát, G., Zavřel, T., Kotabová, E., Kovács, L., Steinbach, G., Vörös, L., Prášil, O., Somogyi, B., Tóth, V.R., 2021. Photomorphogenesis in the Picocyanobacterium Cyanobium gracile Includes Increased Phycobilisome Abundance Under Blue Light, Phycobilisome Decoupling Under Near Far-Red Light, and Wavelength-Specific Photoprotective Strategies. Front. Plant Sci. 12, 1–16. https://doi.org/10.3389/fpls.2021.612302

Holtrop, T., Huisman, J., Stomp, M., Biersteker, L., Aerts, J., Grébert, T., Partensky, F., Garczarek, L., Woerd, H.J. van der, 2021. Vibrational modes of water predict spectral niches for photosynthesis in lakes and oceans. Nat. Ecol. Evol. 5, 55–66. https://doi.org/10.1038/s41559-020-01330-x

Sanfilippo, J.E., Garczarek, L., Partensky, F., Kehoe, D.M., 2019. Chromatic acclimation in cyanobacteria: A diverse and widespread process for optimizing photosynthesis. Annu. Rev. Microbiol. 73, 407–433. https://doi.org/10.1146/annurev-micro-020518-115738

Zavřel, T., Segečová, A., Kovács, L., Lukeš, M., Novák, Z., Pohland, A.-C., Szabó, M., Somogyi, B., Prášil, O., Červený, J., Bernát, G., 2024. A comprehensive study of light quality acclimation in Synechocystis sp. PCC 6803. bioRxiv 2023.06.08.544187. https://doi.org/10.1101/2023.06.08.544187

Comment #4: I do have a major issue with the claim that "In the context of biochemical kinetic modeling such as this one, the justification for manual parameterization over sophisticated algorithms lies in the nuanced understanding and expert knowledge of the system under investigation."

I am fairly familiar with parameter estimation. Algorithms for parameters estimation do indeed allow for prior information ("nuanced understanding and expert knowledge"), they provide a clear understanding about the errors involved. Importantly they also provide information about "identifiability", i.e., which set of parameters are actually constrained by the data. If you claim (as currently in the main text, line 161) that "To avoid overfitting the parameters to a particular experimental set-up, we avoided using sophisticated fitting algorithms and instead ...", and "While fitting algorithms may offer automation and potentially expedite the parameterization process, they often lack the interpretability and domain-specific insight that manual parameterization provides", then please provide appropriate benchmark studies (as a reference) that show shortcomings of such "sophisticated fitting algorithms" over manual fits, and that manual fits avoid overfitting (as opposed to algorithms). I do not know of any such study (and know of plenty that demonstrate the contrary). Also: "Additionally, manual parameterization offers flexibility in addressing model complexities and adapting to unique experimental conditions or system variations that may not be adequately captured by automated algorithms." Which specific "model complexity" and "unique experimental conditions" are you referring to?

Generally: The introduction of ad hoc parameters, such as "we have introduced a light conversion factor" and others, are *textbook examples* of overfitting to suit a particular dataset.

Response: Due to your comment, we wanted to ensure that our now chosen parameters are robust to small perturbations but while allowing for adapting the model to other experimental conditions. Therefore we have conducted the Monte Carlo simulations allowing for factor two perturbations, and we have never found a better set of parameters (see Fig. S14A) that would simultaneously improve our multiobjective: minimal residuals whilst comparing to experimental results from Fig. 1B, Fig. 1C, Fig. S2, Fig. S4 and Fig. 2A and Fig. 2C. Detailed description of the method is now added to the main text of the manuscript (p.9-10). In this analysis we varied the 24 parameters denoted as "manually fitted" in the parameter table (Tab. S2) randomly within a factor two up or down.

We then repeated 10,000 Monte Carlo simulations with a variation factor of 10%, where only 4% of models improved all residuals (Fig. S14B). Therefore, we see that our parametrisation was robust even if our model did not lay directly on the Pareto front.
Furthermore, to test if our manual parameter fitting would compare well against automated methods, we additionally employed a fitting algorithm to minimise the mean over all residuals (including validation data). Simulation with that selected parameter set reproduced the same features and characteristics as our original parameter set (Fig. S15). We conclude that our manual fitting procedure was sufficient to fit the model to the available data.

However, as models with overall better residuals (i.e. closer to the Pareo front) were found, we decided employ such a parameter set as the new set for the model. The set was chosen as the one with the minimal mean residuals to non-validation data (73% of the previous default set). Validation data was not used in the seleciton. We refrained from using the automatically optimised parameter set as the optimization used also validation data. Instead, we selected the parameter set from the Monte Carlo simulations that minimzed only the residuals to non-validation data.

Comment #5: In my opinion any claim about *quantitative* agreement necessarily requires the use of appropriate measures to assess agreement. None of your analysis contains any such analysis of errors (neither with respect to the model, nor with respect to the data). Yet you frequently claim that

the model is quantitative. There are rather oxymoronic sentences close to each other, such as "By harnessing the power of mathematical modelling, we seek to provide a quantitative framework to test further hypothesis ..." and "We do not provide quantitative measures to assess the quality of fits as PAM curves were fitted manually".

This again also applies to figure S3: "Using the same fitted parameters, we can also reproduce the quantitative behavior of cells grown under 633 nm monochromatic light (S3 Figure) .. " Qualitative yes, quantitative: no. If you want to claim quantitative agreement, you must put in the required quantitative analysis. (and I still don't find Fig S3 very convincing, even from a qualitative perspective, let alone quantitative).

Response: Thank you for the push towards a more explicit and quantitative assessment of the quality of our simulations. Indeed where we make claims of quantitative agreement, these claims have to be objectively evaluated. Therefore, we have defined residual function to measure the deviance of our simulations to the data used in the manuscript (Fig 1B & C, Fig 2A & C, S2 Figure, S4 Figure). We did not weight the contribution of each objective within our objective function, considering fit equally important whether it was capturing of electron fluxes, or maximal fluorescence emission.

We have further used these residuals to follow your other previous suggestions and 1) perform automated parameter fitting and 2) show that our parameter set is not arbitrary. We also apologize for misusing “qualitative” and “quantitative” in the cases you named. Considering the strongly different implications of both words, such mistakes are serious and we have given extra care to check that the newest manuscript version is free from any such errors.

Comment #6: Overall figure quality is poor. I don't know if this is because of the downloaded pdf (figures have rather poor resolution in the pdf). Also fonts are too small and difficult to read (see e.g. Fig 5B).

In general, there are only few figures in the main text. The text often refers to Suppl. Figures for key results, e.g. S3. Some of these results should be shown in the main text, if they provide relevant information.

Response: We suspect that the problem occurred while generating the joint PDF by the production and converting our submitted .tiff files. For comparison see below the original quality that we have submitted. We made sure now that the resolutions are kept and we increased the font sizes. If this problem persists with this submission you can download figures separately to check the actual quality. Regarding number of figures: we have included more figures into the main text

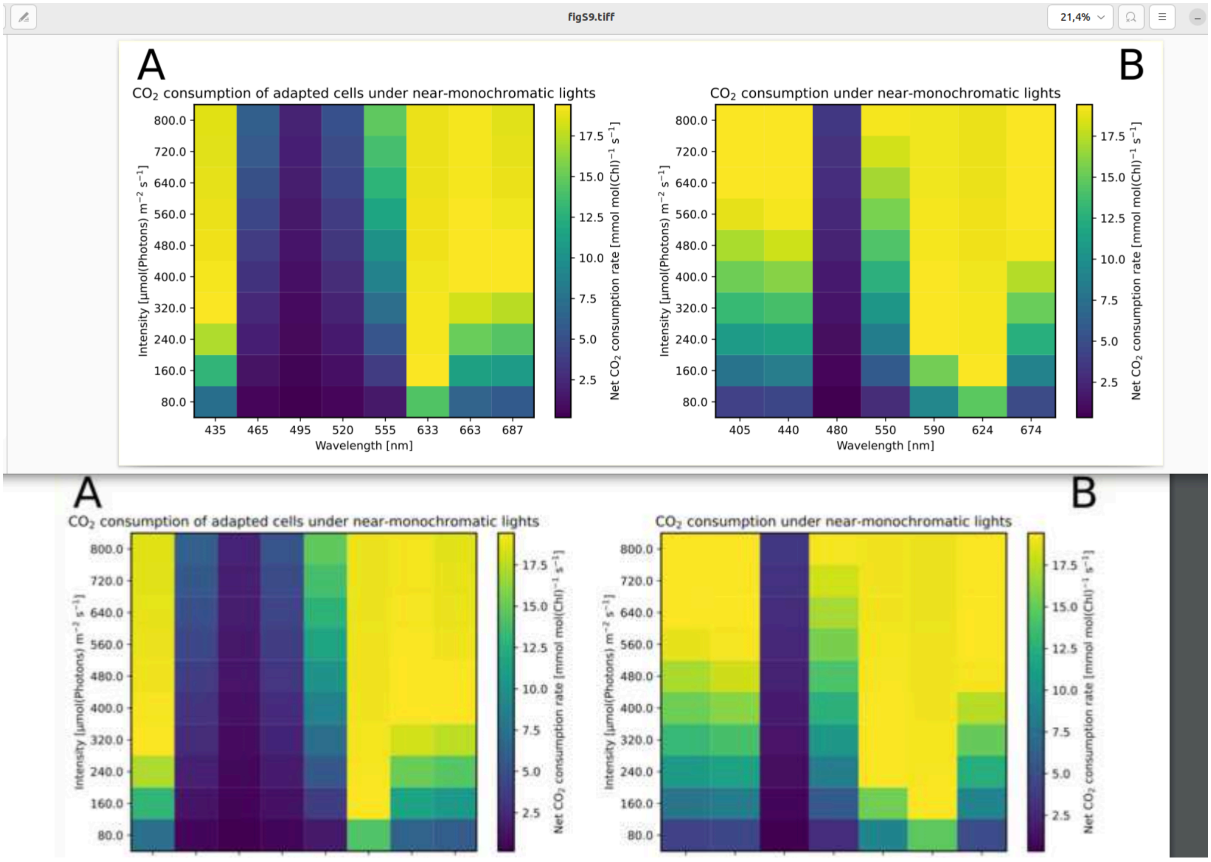

Comment #6: Further on figures. Line 321ff: "Changes in our simulated steady-state O2 evolution rates are in quantitative agreement with the experimental data, during low light and exceed measured rates under light saturation by ca. 20 % (Fig 2C)".

Where is any "quantitative agreement" shown? How does Figure 2C relate to this statement.

Same for: "The model was validated against published measurements of gas exchange rates (Fig 2C) and ...."

Response: Thank you for catching this obvious mistake. These references were supposed to point to Fig. S4, where we simulate the oxygen production and consumption in the dataset by Schuurmans et al. We have corrected incorrect references.

Comment #7: I don't know what Figure S3 shows. The caption says: "S2 Figure. O2 production under light intensity variation in vivo and in vitro."The axis says something else. What is shown there? CO2 or O2? Something else?

Also: It is noted that the simulations and the measured values do not even *qualitatively* agree. The shapes are entirely different. You then define a threshold (ambient CO2) and claim "the intracellular partial pressure by factor 1000 could reproduce the dynamics of O2 evolution for ambient CO2 concentrations and above".

This collides with your claim to "we seek to provide a quantitative framework to test further hypothesis" and is exactly the kind of ad hoc choice described above. Such descriptions make me very sceptical about the general quantitative power of the model. You pick and choose to suit your narrative.

What would stop you to claim "validation" if the points had matched over a wider interval? And If a different dashed line would have been necessary, why not choose a different threshold above which the model fits the data?

If one aims to answer a specific question or hypothesis, such choices may (sometimes) be acceptable, if clearly discussed and justified. But I do not think such an approach can lead to a general quantitative framework, as claimed here ("a comprehensive framework, as a validation of the accuracy of ourcurrent understanding" ... "developing a generic model with validity for more than one condition").

Response: The axis annotation and caption of the S2 figure contain errors. We apologize for this mistake. The plot shows the steady-state production rate of *oxygen* in response to different external CO_2 concentrations. The labelling has been corrected. However, our selection of 400 ppm as the lower bound of the subset of data we try to fit was not set arbitrarily. As you point out, the qualitative behaviour of simulated and measured data differs strongly for CO_2 concentrations below those in ambient air (400 ppm). However, for the current model version, we only envisioned usage cases where cells would be grown under ambient air or supplemented with additional CO_2. Therefore, we manually fit the parameter fCin to the *qualitative* behaviour of the data under those CO_2 concentrations. We agree that for simulations over a wider range of external CO_2 concentrations, the current description of carbon uptake is not sufficient. Quantitatively fitting the data is hindered by the very different oxygen measurements by Benschop *et al.* (ca. 400 mmol mg(Chl)-1 s-1) and Schuurmans *et al.* (ca. 180 mmol mg(Chl)-1 s-1) under similar conditions (high light, 800 and 300 µmol(photons) m-2 s-1, respectively), which cannot be met simultaneously.
Importantly, we fit our model to the dataset by Benschop *et al.*, therefore we do not claim validation - a better fit would only allow for a more accurate parameter prediction.

We understand your reservation for using statements like "general, quantitative framework" and hence we tried to provide a more precise description of our interpretation of what this model provides. Foremost, we provide a modular model that can be used to investigate different cyanobacteria and different phenomena and that can be further detailed if the focus of research shifts and/or new experimental data becomes available.

Comment #8: I assume equation S49 is incorrect, right?

Response: We thank you that you took the time to check the supplementary material. We have checked the equation S49 which describes the effective quantum yield of PSII. According to Schreiber (2019) our description of ΦPSII = (Fm' – Ft) / Fm' is correct. However we see that it might be unclear from the text that we use the effective quantum yield instead of the "optimal". Therefore, we have adapted the manuscript to reflect this.

Comment #9: The model has 27 reactions, the table only lists 22. What are the remaining reactions?

Response: We thank you for catching this mistake. We indeed miscounted the reactions in the model. Additionally, we missed adding two of the model reactions to Table S2: The relaxation of PSII quenched by state transitions and the activation of OCP by light. We have corrected the table and text to represent the correct number of 24 reactions.

Further comments (in no particular order):

- Line 453: "In line with the recent work by Rodrigues et al. (2023) [29], our simulated isoprene also follows the measured cellular growth rate as predicted by their stoichiometric model analysis (S13

Figure)."It follows the growth rate, but not the actual production rate (which is not surprising, since the equation for production is similar to the rate equation for growth).

Response: We agree that our model predictions do not reproduce the measured production rates of isoprene. Instead, the rates predicted by our model compare better to the growth rates. As we noted in the cited text, the GSM model analysis conducted by Rodriues et al. yielded similar results. We interpret this observation such that the optimal isoprene production rate might be proportional to the growth rate. However, regulatory or limiting steps in the production of isoprene, which are not described by either model, might lower the production under certain light conditions. . We are unfortunately not sure how to interpret the comment in the parentheses.

- We have at least one russian reaction: "the reaction doesn’t involve free elections, ..." (line 1351)

Response: While we would appreciate free elections everywhere, this sentence was of course referring to “free electrons”. We have corrected the sentence.

- "However, we disagree with the claim that the model does not capture (chlorophyll fluorescence) dynamics properly. We have captured the trend in both steady-state fluorescence and Fm levels, ..." I never claimed that the model does not "capture properly". I mostly noted that I do not know what "capture properly" means, given the lack of any quantitative analysis of any deviations between data and model.

Response: We hope that our calculation of residuals and the analyses based on them closes this gap and provides a satisfactory quantitative analysis.

- "Because of this, we decided to remain with the manual fitting with a focus on qualitative reproduction of the data."Your text actually claims the opposite. You claim a general quantitative framework.

Response: We have now performed systematic tests to verify accuracy of our manual fits and provided quantifications of how well we reproduce the experimental results to provide evidence for the quantitative power.
