# Supplementary material for: Shedding light on blue-green photosynthesis: A wavelength-dependent mathematical model of photosynthesis in Synechocystis sp. PCC 6803
Source: PLoS Comput Biol. 2024 Sep 12;20(9):e1012445. doi: 10.1371/journal.pcbi.1012445 (PMC11421815; doi:10.1371/journal.pcbi.1012445)
Supplement: Attachment — Submitted filename: Response_to_reviewers.pdf [file pcbi.1012445.s017.pdf]

Junior Professorship Computational Life Science

RWTH AACHEN UNIVERSITY

Tobias Pfennig
Doctoral Researcher
Computational Life Science | Department of Biology
RWTH Aachen University
Worringerweg 1, 52074 Aachen, Germany

12.08.2024

Dear Editor David Lea-Smith,

We would like to express our sincere gratitude for the opportunity to revise and **resubmit** our final version of the manuscript entitled "Shedding light on blue-green photosynthesis: A wavelength-dependent mathematical model of photosynthesis in Synechocystis sp. PCC 6803."

We are thankful that you and reviewer #2 have seen the improvement in our manuscript since its first version. We are happy to address the remaining minor comments.

We hope that the revisions satisfactorily address all concerns and that the manuscript is now suitable for publication. We look forward to your favorable decision and are eager to see our work published in PLOS Computational Biology.

Thank you once again for your time and consideration.

On behalf of all authors
Tobias Pfennig & Anna Matuszyńska

Response to Reviewer 2

Reviewer #2: I only have a few further comments.

1. I am glad to see that the claim that manual fitting is superior to a rigorous computational approach is no longer maintained. This claim did not hold much water.

Answer: We are glad that the improvements we have added into the manuscript have been recognized and accepted.

2. I would still prefer to see an analysis of error (error bars) in Fig. 2F as quantitative agreement is claimed. Error bars are a core of the scientific method and are not an unnecessary inconvenience (at least when quantitative agreement is claimed).

Initially, we could not confidently extract the error bars from the publication of Schuurmans et al. (2014) using an automated graph analyzer (see orange points in the left graph below). Therefore, we have obtained the experimental data from the publication's authors and added the error bars to our graph (see the right graph below). We agree that this analysis of errors improves the credibility of our validation.

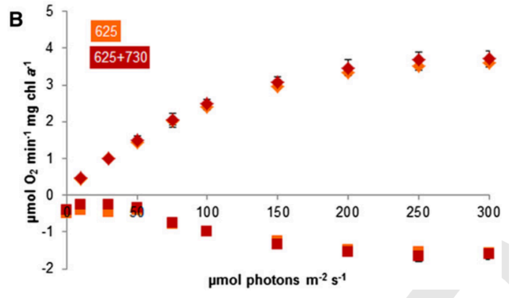

Figure 6. Oxygen evolution rates of *Synechocystis* sp. strain PCC 6803 cultures in increasing intensities of 625-nm light, recorded with MIMS. $^{18}O_2$ was added at the start of the experiment to monitor oxygen consumption in the light. Depicted are net photosynthesis (which is the sum of the measured oxygen production and the consumption [diamonds]) and respiration (squares). A, BG-11 with 0.5 mM $NaHCO_3$. B, BG-11 with 50 mM $NaHCO_3$. Orange symbols, 625-nm light only; red symbols, 625-nm light + 25 μmol photons m^{-2} s^{-1} 730-nm light. Data shown are averages of biological duplicates with SD. [See online article for color version of this figure.]

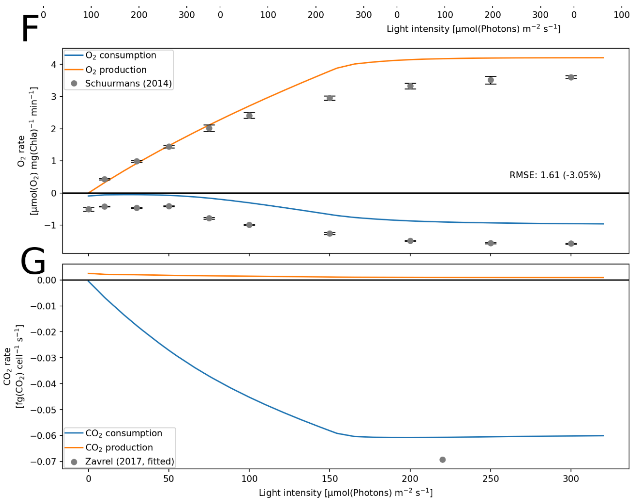

2b. I am actually slightly suspicious about Fig 2F. In a model that is not over-parameterized it is rare that a curve lines exactly on the measured points. I suspect that because you fit electron flow (Fig 2A) and electron flow is stoichiometrically related to O2 evolution (LET minus AEF) you implicitly also fit the O2 evolution (with a deviation at higher light intensities). I do not recall the original publication, but often also experimental works calculate the values this way (that is, the O2 and the electron flow might not have been independently measured in the original publication. Might be worth checking).

Answer: We confirm that the concerned data shown in Fig. 2A (Electron transport) and 2F (gas exchange rates) were measured independently in two different publications and work groups, Theune et al. (2021) and Schuurmans et al. (2014), respectively.

3. The MC sampling is not a replacement for parameter fitting. In particular, when using large intervals (e.g. factor 2 or more) you essentially run the model with random parameters. It is indeed unlikely that those improve upon the original parameterization (as you indeed found out). Small deviations are far more likely to find (local) improvements (that is why step size is important in, for example, evolutionary algorithms, large steps just randomize everything).

Answer: We agree that MC sampling cannot replace data fitting. We performed the MC simulations with large parameter ranges (factor 2) to investigate the parameter space outside of a possible local minimum. Additionally, we performed parameter fitting using a minimization algorithm, as described in section Robustness Analysis (l. 432 - 446).

4. Line 171. You check if the parameterization is close to the Pareto front. But how do you know the Pareto front if you do not have the "best" parameterization (also a brief description in the Materials and Methods would help, not everyone will be familiar with what you actually ("checking if the model is on the Pareto front"), and I am also more guessing that knowing it from the description.

Answer: We consider our parametrization close to the Pareto front based on the Monte Carlo simulations and multi-objective minimization. Both methods showed only minor simultaneous improvement of all objectives and with parameters in close proximity to the original set. We also appended an introduction to Pareto fronts to the beginning of the modeling section (l. 172 - 175):
"We simulated the model with many randomized parameter sets, testing if any set produced simulations with lower residuals to all datasets the model was compared to. If no change to the parameters can improve all of these objectives at once, the parameter set is considered to be on the Pareto front [111]."

5. Fig 1 caption: "the model includes ... four protein complexes (PSII, PSI, cb6f, ATPase)". But the Table includes more (e.g. terminal oxidase, RuBisCO, are these not protein complexes?)

Answer: Changed to *"The model includes descriptions of protein complexes (e.g. PSII, PSI, Cb6f and ATP synthase) and electron carriers in the photosynthetic electron transport chain and the reactions through them, enabling simulation [...]".*

6. Line 89: "Readouts include all intermediate metabolites". Better add "shown in Fig 1", because the model does certainly not include "all intermediate metabolites" (e.g. CBB is lumped into a single reaction)

Answer: As suggested, we changed the sentence to *"Readouts include the intermediate metabolites and carriers shown in Fig. 1, [...]."*

7. Line 404: "On the other hand, our simulated rates lay a factor two below the O_2 production measurements which were used to fit the dynamics of carbon uptake (Fig 2H)". The sentence is confusing. How were O_2 production measurements used to fit the dynamics of carbon uptake? (also weren't O_2 production measurements predicted, and not used to fit?)

Answer: We used the oxygen production measurements by Benschop et al. (2003) to qualitatively fit the dependency of our CO2 uptake function of the external CO2 partial pressure. The magnitude of these measurements is ca. 2 times larger than our simulation results and the measurements by Schuurmans (2014, Fig 2F), therefore only a qualitative fit was possible. The data by Schuurmans was not used for parametrization. We have rephrased the related paragraph to avoid this misunderstanding (l. 408 - 417):
"Our model also includes a description of carbon uptake which was parameterized by fitting simulation results to oxygen production measurements by Benschop et al. [49]. As the maximum of the reported and simulated values differed quantitatively (400 and 200 mmol g(chl)−1 h −1 , respectively) we aimed for a qualitative fit of the dynamics (Fig 2H). Unfortunately, the exact culture conditions (e.g. density) used in the reference work [49] are not known. Hence, factors affecting the estimated oxygen production, such as the pigment composition, may differ, and validation against an independent dataset was necessary. In comparison to measurements by Schuurmans et al. [47], we achieved quantitative agreement under low light and exceeded the measured rates under light saturation by only ca. 20 % (Fig 2F)."

8. From the previous response: "I assume equation S49 is incorrect, right?" I meant equation S50 (or the numbering shifted). It is an ODE for CBBa, the term on the right side is positive (so CBBa would go to infinity). I assume it is not an ODE.

Answer: The activation of the CBB by reduced Ferredoxin (Eq. S50) was indeed implemented as an ODE. Thank you for spotting that the negative term was missing.. The corrected equation is: $\frac{dCBB_a}{dt} = \mathrm{k_{CBBa}} \cdot \left(Fd_{red}^{n_{CBB}} / (Fd_{red}^{n_{CBB}} + K_{Hill,CBB}^{n_{CBB}}) - CBB_a \right)$.
